# Understanding and tuning the electronic structure of pentalenides†‡

**DOI:** 10.1039/d3sc04622b

**Published:** 2024-07-05

**Authors:** Niko A. Jenek, Andreas Helbig, Stuart M. Boyt, Mandeep Kaur, Hugh J. Sanderson, Shaun B. Reeksting, Gabriele Kociok-Köhn, Holger Helten, Ulrich Hintermair

**Affiliations:** a Department of Chemistry, University of Bath Claverton Down Bath BA2 7AY UK u.hintermair@bath.ac.uk; b Institute of Inorganic Chemistry, Institute for Sustainable Chemistry & Catalysis with Boron (ICB), Julius-Maximilians-Universität Würzburg Am Hubland D-97074 Würzburg Germany holger.helten@uni-wuerzburg.de; c Chemical Characterisation Facility, University of Bath Claverton Down Bath BA2 7AY UK

## Abstract

Here we report the first example of systematic tuning of the electronic properties of dianionic pentalenides through a straightforward synthetic protocol which allows the controlled variation of substituents in the 1,3,4,6-positions to produce nine new compounds, representing the largest pentalenide study to date. Both electron-withdrawing as well as electron-donating aromatics have been incorporated to achieve different polarisations of the bicyclic 10π aromatic core as indicated by characteristic ^1^H and ^13^C NMR shifts and evaluated by DFT calculations including nucleus-independent chemical shift (NICS) scans, anisotropy of the induced current density (ACID) calculations, and natural bond orbital (NBO) charge distribution analysis. The introduction of methyl substituents to the pentalenide core required positional control in the dihydropentalene precursor to avoid exocyclic deprotonation during the metalation. Frontier orbital analyses showed arylated pentalenides to be slightly weaker donors but much better acceptor ligands than unsubstituted pentalenide. The coordination chemistry potential of our new ligands has been exemplified by the straightforward synthesis of a polarised *anti*-dirhodium(i) complex.

## Abstract 2

We report the first systematic study of substituent effects on the electronic structure of pentalenides, giving insight into charge distribution, aromaticity and polarisation effects.

## Graphical abstract

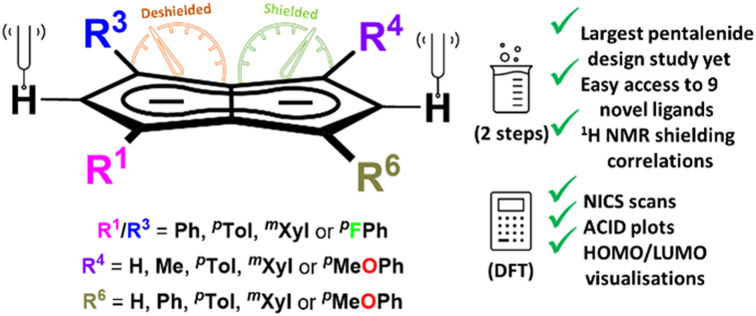

## 1. Introduction

**Scheme 1:**
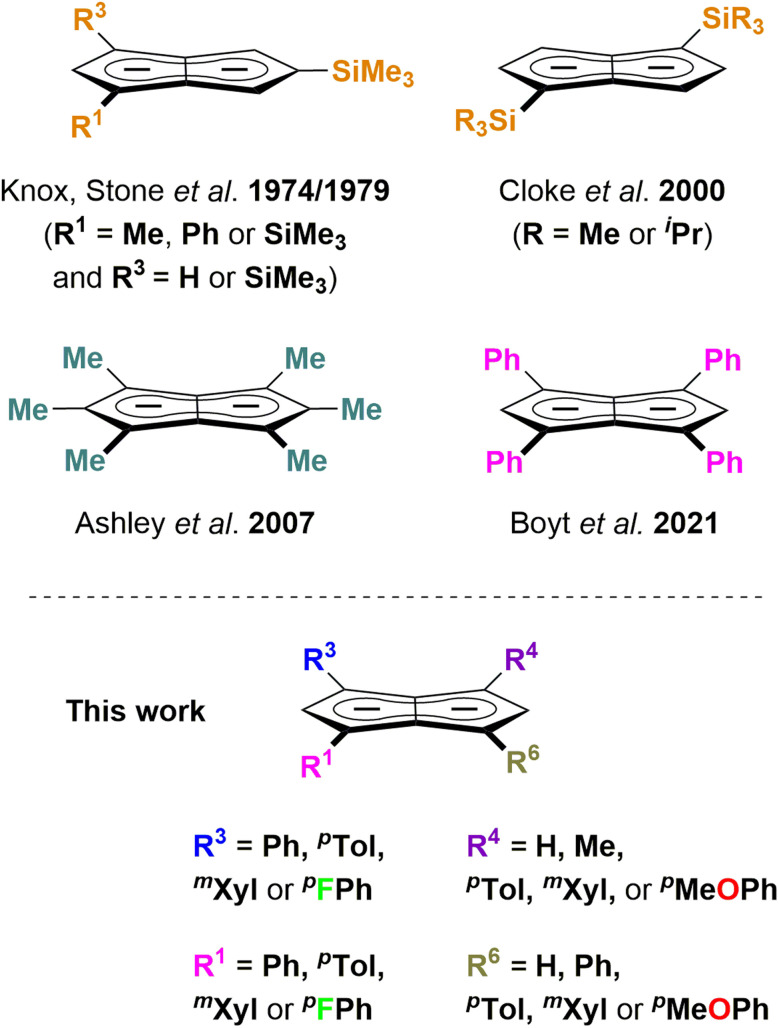
Substituted pentalenides reported to date (top) and work reported here (bottom).

Pentalenides have been intriguing organic and organometallic chemists ever since Katz and Rosenberger reported the first example of the planar 10π aromatic bicyclic **Pn**^**2−**^ (1; C_8_H_6_^2−^) in 1962.^1,2^ Unlike its monocyclic congener **COT**^**2−**^ (C_8_H_8_^2−^) pentalenide ligands can access a variety of bonding modes from η^1^ to η^8^ that allow them to fold around a single metal centre or link two metals together.^2,3^ This ligand manifold has thus been utilised in several mono- and bimetallic d- or f-block organometallic complexes which have found applications in the activation of small molecules such as N_2_ or CO_2_ and in olefin polymerisation catalysis.^3–7^ Compared to the monocyclic **Cp**^**−**^ (C_5_H_5_^−^) ligand class it has remained a rare and rather exotic ligand due to the difficulty of its preparation,^8^ hampering further studies of its organometallic chemistry and utilisation in cooperative bond activation strategies based on multimetallic complexes.^9–12^ No general synthetic protocol which would allow the controlled variation of substituents is known, and to date only eight derivatives of this ligand architecture have been reported (Scheme 1): Knox, Stone *et al.* described four **Pn**^**2−**^ variants obtained *via* thermal rearrangement of substituted cyclooctatetraenes and cyclooctatrienes;^13,14^ Cloke *et al.* subjected unsubstituted **Pn**^**2−**^ to two successive nucleophilic substitution reactions at the 1- and 4-position, creating two bis-silylated variants;^15^ Ashley *et al.* developed a multi-step synthesis of a permethylated **Pn**^**2−**^;^16^ and we recently reported the synthesis of **1,3,4,6-Ph**_**4**_**Pn**^**2−**^ (2^2−^) *via* a two-step solution-phase synthesis from 1,4-Ph_2_CpH and chalcone.^17^ Contrary to the ubiquitous cyclopentadienides^18–21^ no systematic investigation of substituent effects exists, meaning that currently there is no understanding of the extent and nature of electronic variation possible within pentalenides. Here we report the deprotonative metalation chemistry of several substituted dihydropentalenes (**PnH**_**2**_) to yield nine new alkali metal pentalenides with symmetrical and unsymmetrical substitution patterns in 1,3- and 1,3,4,6-positions, and for the first time investigate their electronic effects on the dianionic **Pn**^**2−**^ core by NMR spectroscopy and DFT calculations, including charge distribution analyses, nucleus-independent chemical shift (NICS) scans, and anisotropy of the induced current density (ACID) calculations.

## 2. Results and discussion

### a. Synthesis and properties of symmetrical substitution patterns

**Scheme 2:**
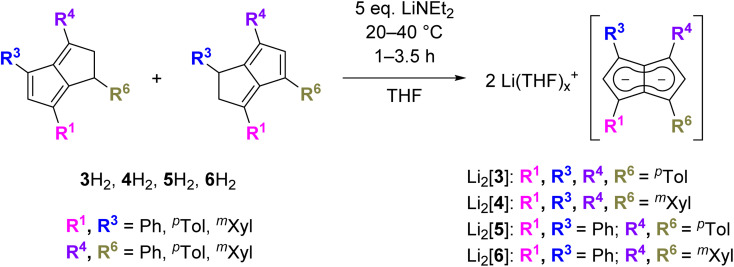
Synthesis of 1,3,4,6-tetrasubstituted pentalenides from their corresponding 1,2-dihydropentalenes (spectroscopically quantitative conversion).

Since the homobimetallic s-block salts of the symmetrically tetraphenyl-substituted [2]^2−^ displayed limited solubility^17^ we investigated the possibility of enhancing solvation *via* the synthesis of the corresponding *p*-tolyl and *m*-xylyl analogues, dilithium 1,3,4,6-tetra-*p*-tolylpentalenide Li_2_[^***p***^**Tol**_**4**_**Pn**] (Li_2_[3]) and dilithium 1,3,4,6-tetrakis(3,5-dimethylphenyl)pentalenide Li_2_[^***m***^**Xyl**_**4**_**Pn**] (Li_2_[4]). Both compounds could be readily synthesised within a few hours at ambient temperature in spectroscopically quantitative yield *via* double deprotonation of the corresponding dihydropentalenes (^***p***^**Tol**_**4**_**PnH**_**2**_ (3H_2_) and ^***m***^**Xyl**_**4**_**PnH**_**2**_ (4H_2_)) with LiNEt_2_ in THF (Scheme 2).^22^ Their excellent solubilities allowed for full characterisation by multi-nuclear NMR spectroscopy as well as mass spectrometry. As was the case for Li·K[2] the ^1^H and ^13^C NMR spectra of Li_2_[3] and Li_2_[4] indicated *D*_2h_ symmetry, and their ^7^Li NMR chemical shifts of +0.7 and −3.8 ppm suggested solvent-separated ion pairs (SSIP) in THF solution at room temperature.^23^ The ^1^H NMR signals for the wingtip protons in 2- and 5-position (denoted H^2^ and H^5^) displayed slight upfield shifts of 0.06 ppm in Li_2_[4] and 0.13 ppm in Li_2_[3] compared to Li·K[2], demonstrating a rather small electronic influence of the *meta*- and *para*-methyl groups on the pentalenide core (see Table 1 as well as Fig. S11–S18‡).

**Fig. 1:**
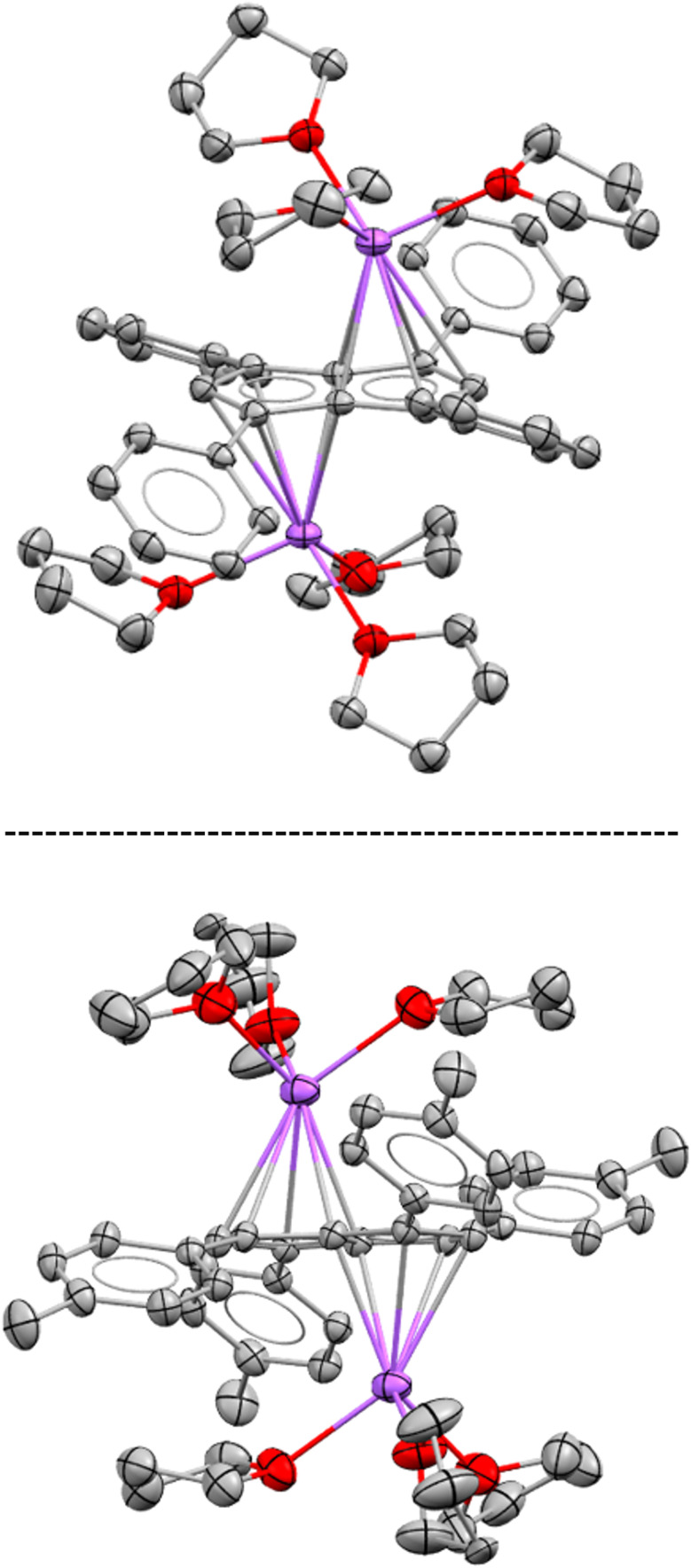
XRD structures of [Na(THF)_3_]_2_[2] (top) and [Na(THF)_3_]_2_[3] (bottom) at 50% thermal probability with hydrogen atoms omitted for clarity (for details see the Experimental section).

While both M_2_[3] and M_2_[4] showed markedly higher solubility than M_2_[2] (M = Li, Na, K) in THF, crystalline material could still be obtained from concentrated solutions in all cases. The crystal structure of [Na(THF)_3_]_2_[3] obtained from using 2.1 equivalent NaNH_2_ as the base (Scheme 2) was found to be iso-structural to that of [Na(THF)_3_]_2_[2],^17^ both containing two solvated Na^+^ ions in *anti*-configuration (Fig. 1).

In both molecules the aryl substituents were bent away from the plane of the pentalenide core by 14–19°, with a pair at one ring pointing upwards and the other pair downwards. As no significant aryl–metal interactions were found in the XRD data of either, and the ^1^H and ^13^C NMR chemical shifts of Li·K[2] and Li_2_[3] were similar in solution (Tables 1 and S1‡), the observation that their substituents had opposite configuration in the solid state (bent towards the metals in [Na(THF)_3_]_2_[2] but bent away from the metals in [Na(THF)_3_]_2_[3]) can be ascribed to packing effects that suggest a degree of flexibility in these molecules. This bending of the aryl substituents also seemed to have influenced the hapticity of the sodium ions: analysing the crystallographic data with the metal slippage vector model,^24^ [Na(THF)_3_]_2_[3] had 78% η^5^ and 22% η^2^ character while [Na(THF)_3_]_2_[2] showed a more ambiguous pattern with a larger coordination preference towards the C–C bridge (58% η^5^ and 42% η^2^ character). In addition to this substituent bend, the aryl substituents were twisted by 15–26° relative to the planar pentalenide core in both structures, but only one set of magnetically equivalent aryl protons were observed in their room temperature NMR spectra, suggesting rapid flipping in solution. This flexible, pairwise substituent bend and twist found in these tetra-substituted pentalenides is likely to relieve steric repulsion between the aryl groups in 1,3 and 4,6 positions, and has implications for electronic substituent effects by affecting the degree of π overlap between the aryl groups and the pentalenide core (see below). As previously found for [2]^2−^, the use of a heterobimetallic combination of two different alkali metals improved the solubility further: subjecting 4H_2_ to sequential deprotonation with first KHMDS and then LiNEt_2_ cleanly furnished Li·K[4] which did not precipitate from THF for more than 12 months and allowed solution phase analysis at variable temperature. Cooling a sample of Li·K[4] in THF to −95 °C resulted in decoalescence of the *o*-aryl and methyl protons of the *m*-xylyl substituents in the ^1^H NMR spectrum at 500 MHz below −65 °C, indicating a slowed oscillation of the aryl substituents (Fig. S54–S57‡). The rate constant for the NMR exchange was determined to be 1500 s^−1^ at −80 °C, where the peaks at 7.24 and 5.85 ppm coalesced, representing a Δ*G*^‡^ value of 8.55 kcal mol^−1^. No significant changes in the ^7^Li chemical shift were observed during the VT NMR experiment, ruling out a reduction in symmetry of [4]^2−^ through increased ion pairing at lower temperatures.

**Table 1:** Pentalenide positional nomenclature and ^1^H NMR chemical shifts of wingtip protons in variously substituted dilithium pentalenides (in THF at room temperature)

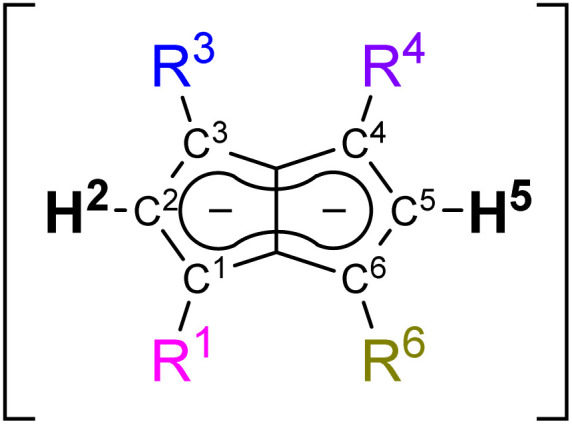							
**Pn** ^**2**^ **^−^**	R^1^	R^3^	R^4^	R^6^	*δ*(H^2^) [ppm]	*δ*(H^5^) [ppm]	Δ*δ*(H_w_) [ppm]
1	H	H	H	H	5.76 ^15^	5.76 ^15^	—
2	Ph	Ph	Ph	Ph	6.79^a^	6.79^a^	—
3	^*p*^Tol	^*p*^Tol	^*p*^Tol	^*p*^Tol	6.66	6.66	—
4	^*m*^Xyl	^*m*^Xyl	^*m*^Xyl	^*m*^Xyl	6.73	6.73	—
5	Ph	Ph	^*p*^Tol	^*p*^Tol	6.72	6.66	0.06
6	^*m*^Xyl	^*m*^Xyl	Ph	Ph	6.79	6.73	0.06
7	^*p*^MeO-Ph	^*p*^MeO-Ph	Ph	Ph	6.56	6.74	0.18
8	^*p*^F-Ph	^*p*^F-Ph	^*p*^Tol	^*p*^Tol	6.68	6.63	0.05
9	Ph	Ph	H	H	7.05	6.06	0.99
10	^*p*^F-Ph	^*p*^F-Ph	H	H	6.92	6.06	0.86
12	Me	Ph	Ph	Ph	6.19	6.67	0.48

a Average of values reported for heterobimetallic **Ph**_**4**_**Pn**^**2−**^ soluble in THF.^17^

### b. Synthesis and properties of unsymmetrical substitution patterns

Asymmetric pentalenides with different substituents on each ring are rare. In the 1970s Knox and Stone *et al.* reported two examples of mono-substituted cyclooctatetraenes that isomerised in the presence of [Ru_2_CO_4_(SiMe_3_)_2_] to give silyl-substituted di-ruthenium pentalenide complexes which were isolated in <1% yield and characterised by ^1^H NMR and IR spectroscopy.^13,14^ To the best of our knowledge, no method for accessing pentalenides with different substituents on each ring in synthetically useful yields has ever been reported.^8^ Independent control over the steric and electronic properties of each ring of the fused pentalenide core promises access to well-defined heterobimetallic complexes with potentially interesting electrochemical, photophysical and catalytic properties.^9–12^ We thus investigated the deprotonation of **Ph**_**2**_^***p***^**Tol**_**2**_**PnH**_**2**_ (5H_2_) and **Ph**_**2**_^***m***^**Xyl**_**2**_**PnH**_**2**_ (6H_2_) as their respective double bond isomer mixtures^22^ with LiNEt_2_ in THF (Scheme 2). Pleasingly, Li_2_[**Ph**_**2**_^***p***^**Tol**_**2**_**Pn**] (Li_2_[5]) and Li_2_[**Ph**_**2**_^***m***^**Xyl**_**2**_**Pn**] (Li_2_[6]) were cleanly obtained in spectroscopically quantitative yields, as in the case of their symmetrical congeners Li_2_[3] and Li_2_[4]. Both showed the same SSIP character in THF solution at room temperature and were fully characterised by NMR spectroscopy and high-resolution mass spectrometry (Fig. S19–S26‡). Likely as a result of their reduced symmetry (time-averaged *D*_2d_), the homobimetallic di-lithium salts remained fully soluble at 10^−2^ M concentration in THF for several months at room temperature. Interestingly, both [5]^2−^ and [6]^2−^ displayed a distinct chemical shift difference of 0.06 ppm for their respective wingtip protons H^2^ and H^5^, indicative of a different polarisation of the two five-membered rings of the pentalenide core.

**Scheme 3:**
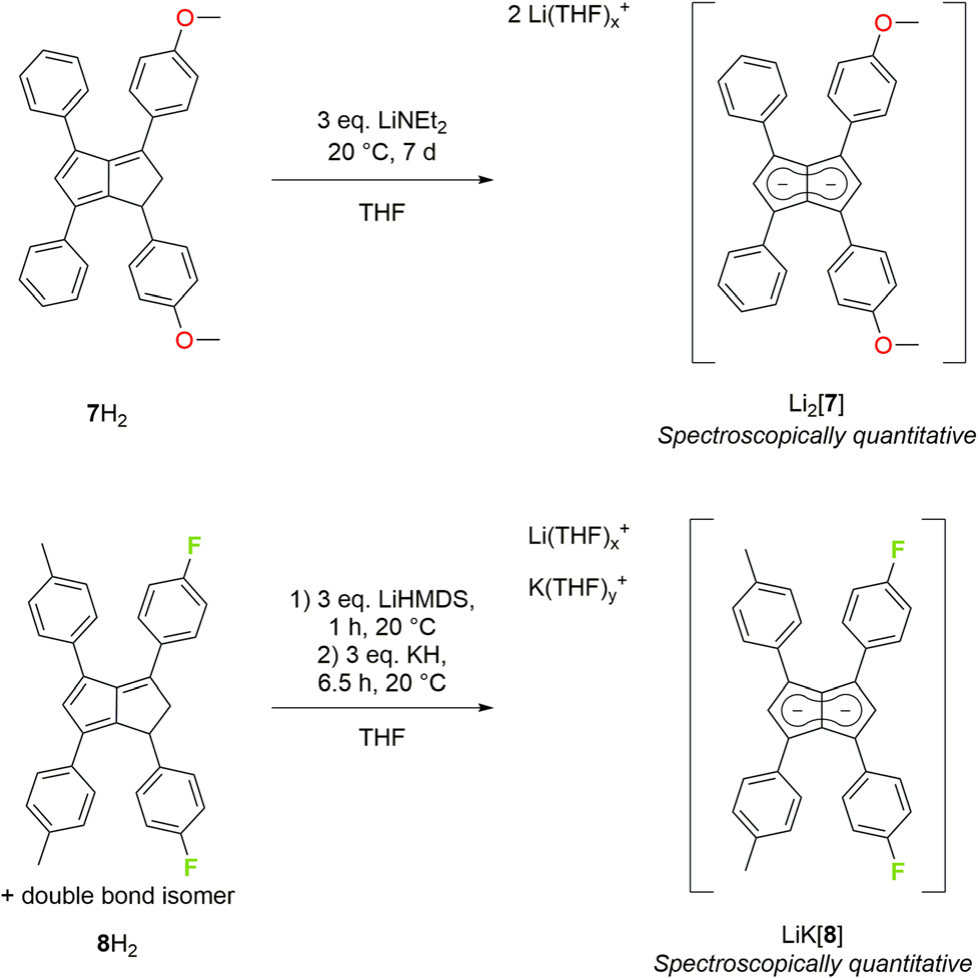
Synthesis of 4-methoxyphenyl substituted (top) and 4-fluorophenyl substituted pentalenides (bottom).

To increase the polarisation of the two pentalenide moieties further, we investigated the metalation of **(**^***p***^**MeOPh)**_**2**_**Ph**_**2**_**PnH**_**2**_ (7H_2_)^22^ bearing more electron-donating aryl groups than tolyl or xylyl. Its deprotonation with an excess of LiNEt_2_ did not occur as quickly as with 3–6H_2_ but required several days to go to completion, presumably due to a higher p*K*_a_ of the five-membered ring with the electron-donating ^*p*^MeOPh groups. However, after seven days at room temperature Li_2_[**(**^***p***^**MeOPh)**_**2**_**Ph**_**2**_**Pn**] (Li_2_[7]) – the first oxygenated pentalenide – was cleanly formed in spectroscopically quantitative yield (Scheme 3). ^1^H NMR analysis showed that the two electron-donating ^*p*^MeOPh groups led to a 0.18 ppm chemical shift difference between the wingtip protons H^2^ and H^5^ in [7]^2−^, three times the polarisation seen in [5]^2−^ and [6]^2−^ (Table 1). Interestingly, its ^7^Li NMR chemical shift of −4.78 ppm suggested a stronger interaction of the Li^+^ with the pentalenide than in 2–6, more in the range of a solvent-shared ion pair (Fig. S27–S29‡).^25^

To see if the introduction of aryl substituents containing electron-withdrawing groups could lead to further deshielding effects on the pentalenide core, we investigated the deprotonative metalation of an isomeric mixture of **(**^***p***^**FPh)**_**2**_**(**^***p***^**Tol)**_**2**_**PnH**_**2**_ (8H_2_).^22^ LiHMDS in THF was found to be ineffective in furnishing the corresponding pentalenide salt, implying its p*K*_a_ to be insufficient for the double deprotonation of a dihydropentalene with either *p*-tolyl or 4-fluorophenyl in the 1,3-positions (unlike in the preparation of Li_2_[2]). Using a mixture of LiHMDS and LiNEt_2_ showed full consumption of the starting materials by ^1^H NMR spectroscopic analysis after 7.5 hours at room temperature, but with a multitude of new, broad signals that could not be confidently assigned. Utilising a heterobimetallic base combination of LiHMDS followed by KH (which had proven successful for 2H_2_ and 4H_2_) resulted in the clean formation of the first fluorinated pentalenide Li·K[**(**^***p***^**FPh)**_**2**_**(**^***p***^**Tol)**_**2**_**Pn**] (Li·K[8]) in spectroscopically quantitative yield after 6.5 hours (Scheme 3) which was fully characterised by NMR spectroscopy and mass spectrometry (Fig. S30–S32‡). The weakly electron-withdrawing ^*p*^FPh groups in Li·K[8] led to a marginal ^1^H NMR chemical shift difference between H^2^ and H^5^ of 0.05 ppm, indicating a rather weak influence on the polarisation of the pentalenide core (Table 1).

**Scheme 4:**
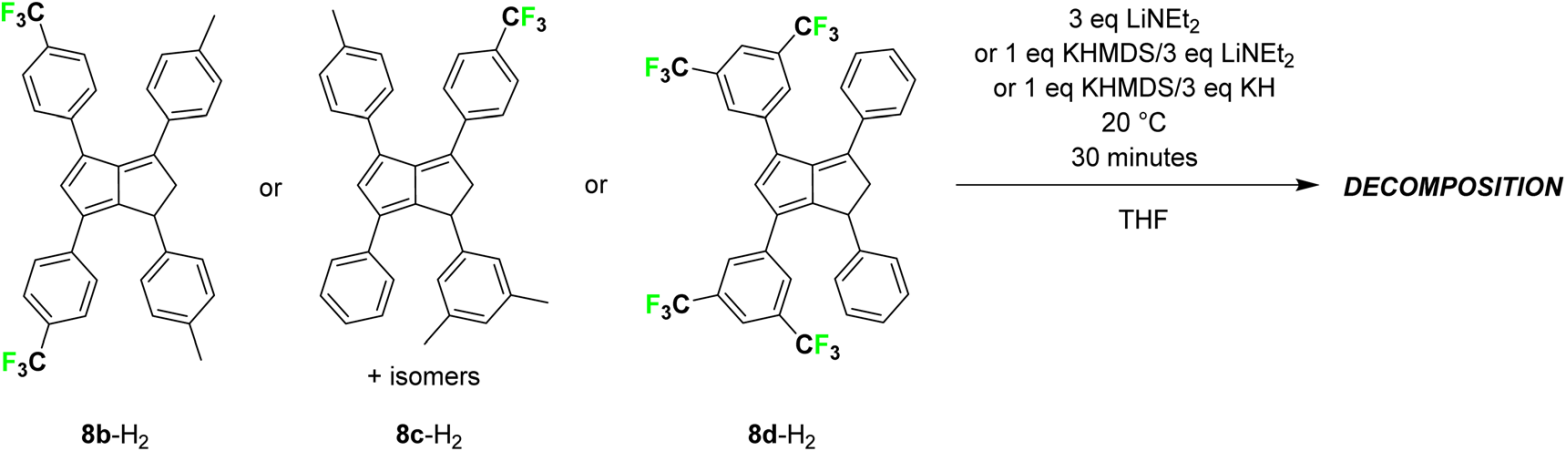
Attempted deprotonative metalations of (CF_3_)_*x*_Ph-substituted dihydropentalenes 8b-H_2_, 8c-H_2_ and 8d-H_2_ (*x* = 1 or 2).

To increase the electron-withdrawing nature of the substituents on one side of the pentalenide further we also tried to deprotonate the ^*p*^CF_3_Ph-substituted dihydropentalene **((**^***p***^**F**_**3**_**C)Ph)**_**2**_**(**^***p***^**Tol)**_**2**_**PnH**_**2**_ (8b-H_2_).^22^ However, *in situ* NMR analysis showed that bases of higher p*K*_a_ values than LiHMDS led to unidentifiable heterogeneous mixtures indicative of decomposition (Scheme 4). All attempts of deprotonating a double bond isomer mixture of **(**^***m***^**Xyl)(Ph)(**^***p***^**Tol)((**^***p***^**F**_**3**_**C)Ph)PnH**_**2**_ (8c-H_2_), where the CF_3_ groups were located in *para*-position but the fluorinated aryl group positioned differently in each dihydropentalene isomer,^22^ led to the same observation (as representative example, see Fig. S58 and S59‡). Finally, to investigate whether CF_3_ groups themselves or their location in the aryl substituents of the dihydropentalenes were incompatible with the basic conditions required for their metalation, we tested the deprotonation of **(Ph)**_**2**_**((**^***m***^**F**_**3**_**C)**_**2**_**Ph)**_**2**_**PnH**_**2**_ (8d-H_2_; product of the condensation of 1,4-diphenyl-cyclopenta-1,3-diene^17^ and 1,3-bis(3,5-bis(trifluoromethyl)phenyl)-2-propen-1-one;^26^ see the ESI‡). As observed with the other two CF_3_-functionalised dihydropentalenes, bases of similar or weaker strength than LiHMDS did not lead to pentalenide formation and stronger amide bases led to decomposition, demonstrating the incompatibility of aryl-CF_3_ substituents in this deprotonative metalation protocol of dihydropentalenes with ionic bases of p*K*_a_ >35.

**Scheme 5:**
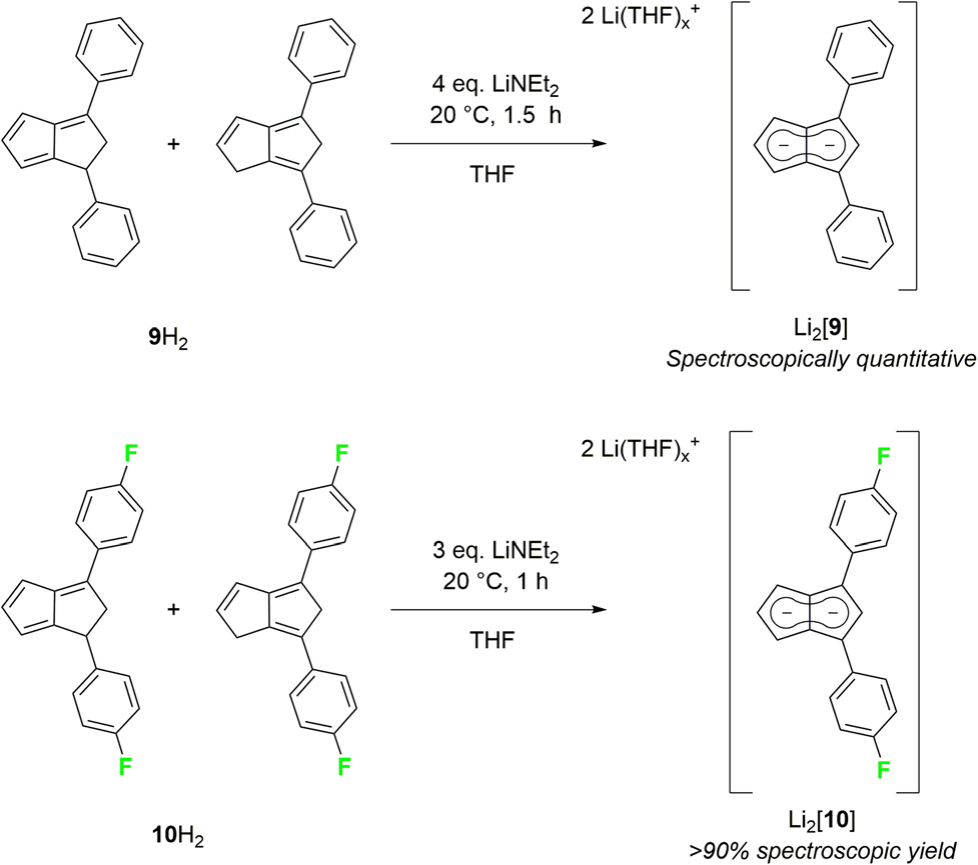
Synthesis of 1,3-diarylated pentalenides.

As strongly electron-withdrawing aryl substituents were found to be incompatible with deprotonative metalations using alkali metal bases, we investigated 1,3-diarylated dihydropentalenes featuring one unsubstituted five-membered ring. An isomeric mixture of **Ph**_**2**_**PnH**_**2**_ (9H_2_) was readily prepared from **CpH** and chalcone following Griesbeck's protocol.^27^ Their deprotonation with LiNEt_2_ in THF proceeded smoothly to give Li_2_[**Ph**_**2**_**Pn**] (Li_2_[9]) as SSIP in spectroscopically quantitative yield after 1.5 hours at room temperature (Scheme 5).

Intriguingly, [9]^2−^ (representing an intermediate between [1]^2−^ and [2]^2−^) displayed drastically different wingtip chemical shifts in the ^1^H NMR spectrum: while H^5^ on the unsubstituted five-membered ring resonated at 6.06 ppm, H^2^ on the diphenylated pentalenide moiety resonated at 7.05 ppm to give a chemical shift difference of 0.99 ppm (Table 1). This represents the strongest polarisation of any pentalenide reported yet, and suggests a markedly different electronic environment in each ring. Compared with [1]^2−^, the introduction of the 1,3-Ph_2_ substituents caused a mild remote deshielding of H^5^ on the unsubstituted ring of 0.30 ppm (from 5.76 ppm (ref. 15) to 6.06 ppm) but caused an even more pronounced deshielding effect on H^2^ in the substituted ring than in [2]^2−^ (7.05 ppm in [9]^2−^*versus* 6.79 ppm in [2]^2−^). The reaction of an isomeric mixture of **(**^***p***^**FPh)**_**2**_**PnH**_**2**_ (10H_2_; see Chapter 2 of the ESI‡) with LiNEt_2_ led to the analogous Li_2_[**(**^***p***^**FPh)**_**2**_**Pn**] (Li_2_[10], Scheme 5), in which to our surprise the ^*p*^FPh substituents caused a slightly poorer deshielding than the phenyl substituents in Li_2_[9]: with a ^1^H NMR chemical shift of 6.92 ppm at H^2^, [10]^2−^ exhibits the second largest pentalenide polarisation based on a chemical shift difference of 0.86 ppm (6.06 ppm for H^5^).

**Scheme 6:**
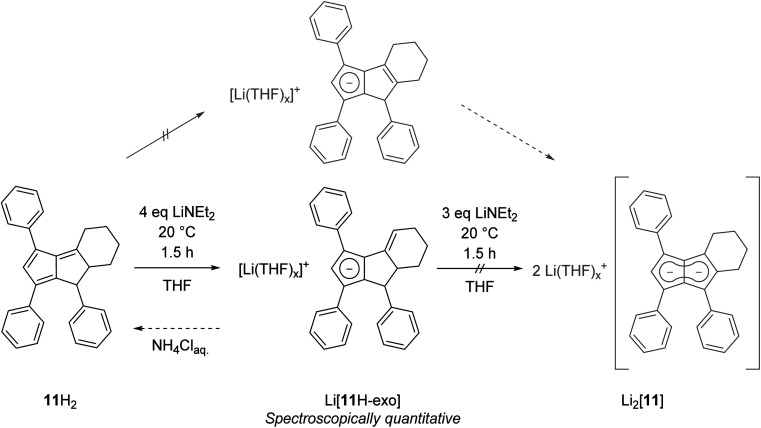
Exocyclic deprotonation of 11H_2_ to 11H-*exo*.

Finally, we investigated dihydropentalenes containing aryl as well as alkyl substituents to see if the electron-donating nature of the latter would serve to tune the electronic polarisation in unsymmetrically substituted pentalenides further. We therefore synthesised the tricyclic dihydropentalene 1,3,8-triphenyl-4,5,6,7,7*a*,8-hexahydro-cyclopenta-[*a*]-indene 11H_2_ (see Chapter 2 of the ESI‡ as well as Fig. S9, S10 and S63‡) featuring three aryl and two alkyl substituents. However, when attempting its metalation with LiNEt_2_ in THF we observed the quantitative formation of the monoanionic hydropentalenide isomer Li[11H-*exo*] featuring an exocyclic double bond in the alkyl substituent in 3-position (Scheme 6 and Fig. S42–S44‡). All attempts to convert this species into the desired dianionic pentalenide Li_2_[11] with an excess of LiNEt_2_ failed. Protolysis of Li[11H-*exo*] restored 11H_2_ with its pentafulvene double bond structure in 25% spectroscopic yield (Scheme 6).

**Scheme 7:**
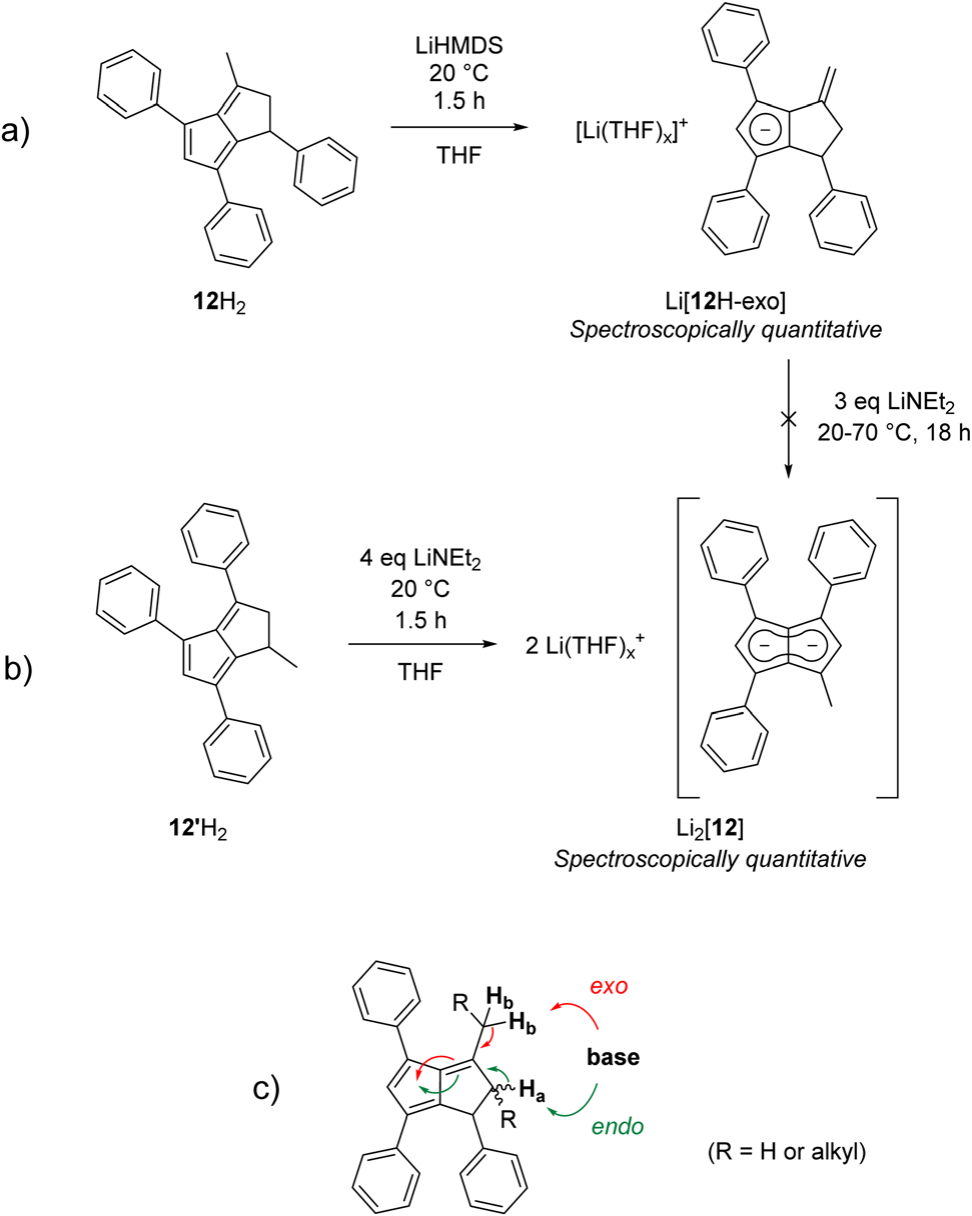
(a) Exocyclic deprotonation of 12H_2_ to Li[12H-*exo*] with LiHMDS; (b) formation of Li_2_[12] from 12′H_2_ with LiNEt_2_; (c) allylic deprotonation competition in 3-alkylated dihydropentalenes.

To investigate whether this exocyclic double bond formation during the deprotonation of 11H_2_ was due to the cyclic structure of the alkyl substituents (perhaps caused by conformational effects) we investigated **3-Me-1,4,6-Ph**_**3**_**PnH**_**2**_ (12H_2_; Fig. S62‡)^22^ featuring a methyl group in 3-position instead of the fused cyclohexyl ring in 11H_2_. Deprotonation of 12H_2_ with one equivalent of LiHMDS quickly generated the exocyclic double bond isomer Li[12H-*exo*] analogous to Li[11H-*exo*] (Scheme 7a). As seen with the latter, all attempts to convert Li[12H-*exo*] to Li_2_[12] by treating it with a stronger base and/or heating to 70 °C failed, showing exocyclic double bond formation to be a dead end for pentalenide formation from 1,2-dihydropentalenes featuring alkyl substituents in the 3-position. Indeed, the analogous deprotonations of **1,3-Me**_**2**_**-4,6-Ph**_**2**_**PnH**_**2**_ ^22^ and **1,3-Me**_**2**_**-PnH**_**2**_ ^28^ with LiNEt_2_ or ^*n*^BuLi led to the same observation, and similar reactivity has been reported for the deprotonative metalation of **1,2,3,4,5,6-Me**_**6**_**PnH**_**2**_ with ^*n*^BuLi.^29^ This observation is likely due to these monoanionic intermediates being more allylic cyclopentadienides than hydropentalenides, meaning that although they still feature a methine hydrogen in the 1-position this is not acidified by being bound to the only sp^3^ carbon between a double bond and a **Cp**^**−**^ (ready to aromatise to one conjugated 10π system) as in an endocyclic hydropentalenide. This exocyclic deprotonation of alkyl groups in the 3-position of 11H_2_, 12H_2_ and related 3-alkylated dihydropentalenes is likely the result of kinetic competition, where the base can attack either one of two allylic positions of similar p*K*_a_ leading to either endocyclic or exocyclic double bond formation (Scheme 7c). The hydrogens on the alkyl substituent being sterically more accessible and statistically dominant over the ring-bound hydrogens (2 : 1 in 11H_2_ and 3 : 2 in 12H_2_) thus leads to predominant formation of the undesired allyl-cyclopentadienides with sterically demanding amide bases akin to the reactivity of 6,6-dialkylpentafulvenes.^30,31^ Unsubstituted and arylated dihydropentalenes avoid this issue by their lack of exocyclic allylic sites for competing deprotonation.

We hypothesised that if exocyclic double bond formation was indeed due to allylic deprotonation competition, then installing a methyl substituent in the 1-position and blocking exocyclic deprotonation with an aryl substituent in the 3-position should avoid this issue. We thus designed **1-Me-3,4,6-Ph**_**3**_**PnH**_**2**_ (12′H_2_), a double bond isomer of 12H_2_, by cyclising 1,3-Ph_2_CpH with (*E*)-1-phenylbut-2-en-1-one (see Chapter 2 of the ESI‡). To our delight, treating 12′H_2_ with three equivalents of LiNEt_2_ resulted in the clean formation of Li_2_[**MePh**_**3**_**Pn**] (Li_2_[12]) – the first mixed aryl-alkyl pentalenide – in spectroscopically quantitative yield (Scheme 7b and Fig. S47–S50‡). No signs of hydropentalenides (endo- or exocyclic) were observed in the reaction, and Li_2_[12] was stable in THF solution without any signs of rearrangement or decomposition for several months under inert conditions. Comparing [12]^2−^ with [2]^2−^, the substitution of a phenyl substituent with single methyl group led to a substantial polarisation of the pentalenide core as indicated by a significant ^1^H NMR wingtip chemical shift difference of 0.48 ppm (Table 1). Crystal structures of the new dihydropentalenes 11H_2_, 12H_2_ and 12′H_2_ can be found in the ESI (Sections 8.2–8.4‡).

### c. Computational analysis of electronic structure

**Fig. 2:**
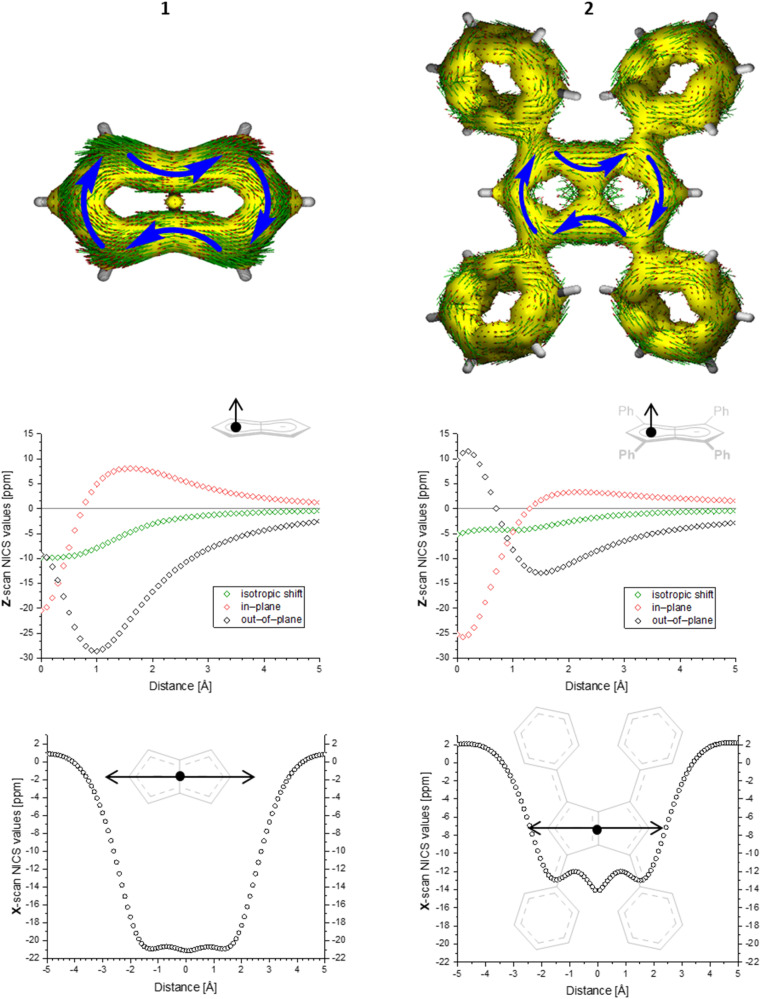
ACID plots (top; isovalue = 0.025) and NICS scans (bottom; *X* scan at a *Z* height of 1.7 Å, NICS probe BQ shown as •) of [1]^2−^ (left) and [2]^2−^ (right).

**Fig. 3:**
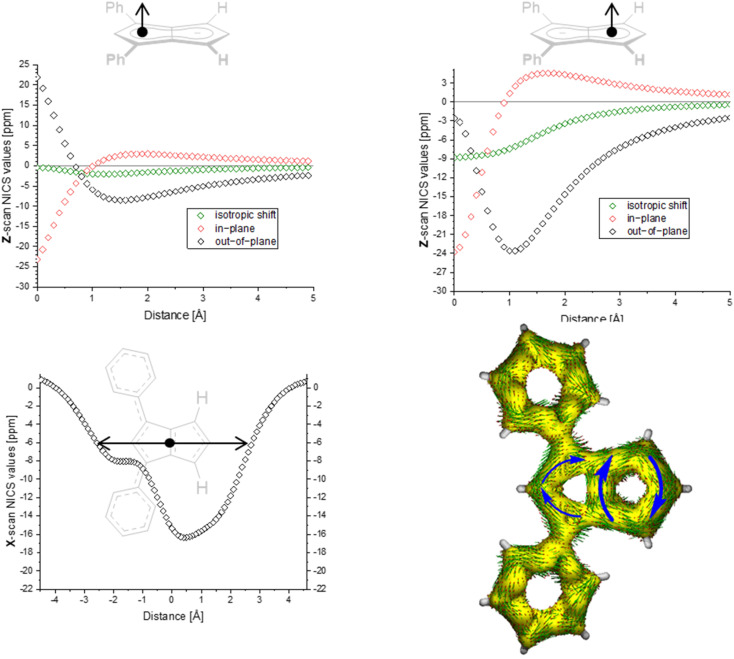
ACID plot of [9]^2−^ (isovalue = 0.025) and NICS scans of [9]^2−^ (*X* scan at a *Z* height of 1.7 Å, NICS probe BQ shown as •).

**Fig. 4:**
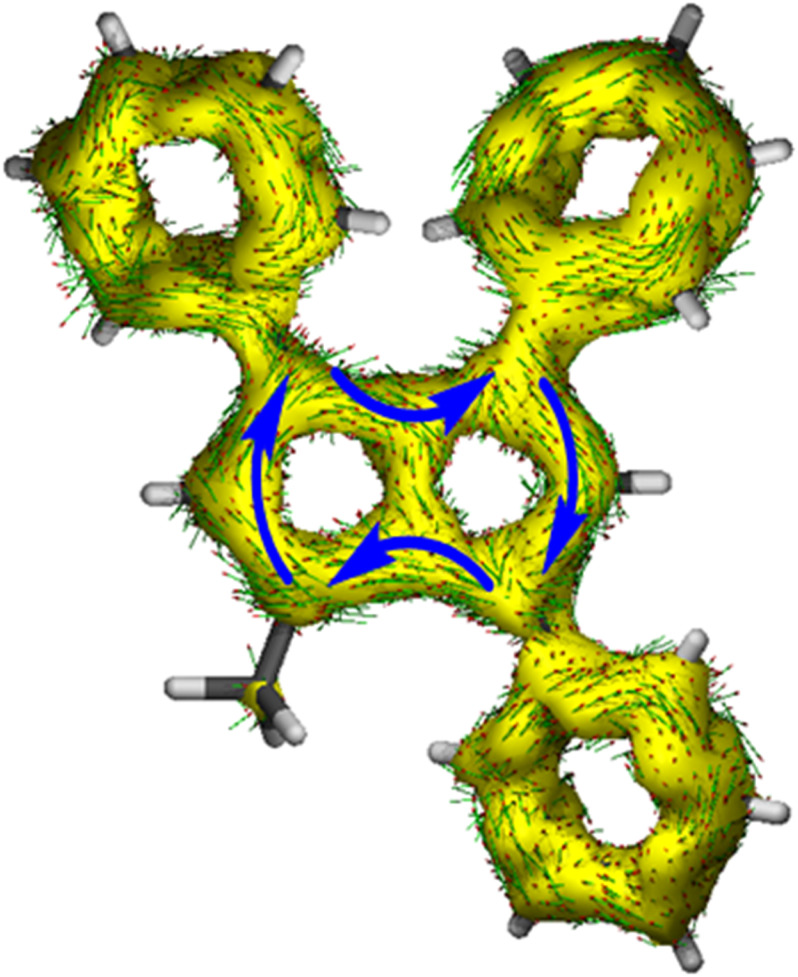
ACID plot of [12]^2−^ (isovalue = 0.025).

To gain deeper insight into the electronic structures of these new pentalenides and understand the polarisation effects introduced by the various substituents, we carried out DFT calculations on [2]^2−^, [3]^2−^, [9]^2−^, [10]^2−^, and [12]^2−^ in comparison with the parent [1]^2−^. To better align the calculated geometries with the solid-state molecular structures from X-ray crystallography and obtain meaningful frontier orbital energies we included two Li^+^ counterions in *trans* η^5^ position as found in the solid state. For aromaticity and charge distribution calculations we used the bare dianions as a more realistic models for the speciation in solution where solvent-separated ion pairs exist (see above). Additionally, this avoids possible distortions of charge localisation due to coulomb attraction. The computed geometries for Li_2_[1–3] agreed well with the experimentally determined solid-state XRD structures respectively,^17,32^ including the ∼30° twist of the aryl substituents relative to the pentalenide core. To elucidate the degree of aromaticity in the conjugated π systems of arylated pentalenides, we calculated the anisotropy of the induced current density (ACID) as well as nucleus-independent chemical shift (NICS) scans along different axes across the molecules (as indicated in the insets in Fig. 2–4).

This analysis revealed that the pentalenide core was clearly aromatic in all compounds but to different extents. The ACID plot of [1]^2−^ showed a strong diatropic (aromatic) ring current around the C_8_ perimeter of the pentalenide excluding the transannular C–C bond (Fig. 2, top). This finding is consistent with previous MO analyses showing haptotropic mobility of Lewis-acidic metals bound to the dianionic 10π system to be confined to the perimeter, with a forbidden path across the central C–C bridge due to unfavourable orbital overlap.^33^ The ACID plots of [2]^2−^ (Fig. 2) and [3]^2−^ (Fig. S67‡) also showed global diatropic ring currents within the pentalenide system but with larger contributions of the transannular C–C bond, in addition the local diatropic ring currents within the four aromatic substituents.

The NICS scans demonstrated a pronounced difference in the degree of the aromatic character of the different pentalenides investigated. Comparison of [1–3]^2−^ showed that the unsubstituted [1]^2−^ was the most aromatic pentalenide within this series. In each case, the NICS scan along the *Z* axis starting from the centre of one five-membered ring perpendicular to the plane of the pentalenide core was indicative of an aromatic ring current. The isotropic shift was negative throughout the scan, and the shape of the curve was mainly governed by the out-of-plane contributions to the isotropic shift with a clear minimum. For [1]^2−^, the calculated NICS at that minimum (*Z* = 1.0 Å) was −28.6 ppm, which is in the same range as that of benzene (−29.1 ppm at 1.0 Å) and slightly higher than that of **Cp**^**−**^ (−33.8 ppm at 0.9 Å).^34^ For [2]^2−^ and [3]^2−^ the minima were significantly higher than in [1]^2−^, with −12.9 ppm for [2]^2−^ and −12.5 ppm for [3]^2−^ at 1.4 Å each (see also Fig. S67‡).

The NICS *X*-scans for [1–3]^2−^ showed a plateau of maximum diatropicity extending over the two five-membered rings, with the shallow minima above the bonds originating from σ-effect contaminations.^35^ This feature is again consistent with an induced ring current over the ellipsoidal pentalenide perimeter, as seen in the ACID maps. The *X*-scans should allow to differentiate whether this was due to a global current over the entire pentalenide system, or the result of superposition of local ring currents in the two fused cyclopentadienyl subunits.^35^ If the latter was the case, the absence of a significant current density at the transannular C–C bond, which is most pronounced in the ACID plot of [1]^2−^, would be the result of a net cancelation of two counter-currents across this linkage. To answer this question, the NICS *X* values of [1]^2−^ and the parent cyclopentadienide were compared. In the case of a superposition of two local **Cp**^**−**^ ring currents, the NICS values at 1.7 Å above the central C–C bridge of [1]^2−^ (−21.1 ppm) should be approximately twice the value for [**Cp**]^−^ at the same *Z* height at 1 Å from its ring centre (−12.8 ppm), which is equivalent to the distance between the middle of one pentalenide ring and the transannular C^3′^–C^6′^ bond in [1]^2−^. As this was not the case, it leads to the conclusion that no local cyclopentadienide ring currents exist in [1]^2−^, and the ring current is due to an overall pentalenide circuit instead. This situation prevailed in [2]^2−^, where the reference 1,3-diphenylcyclopentadienide (**1,3-Ph**_**2**_**Cp**^**−**^) had a NICS *X* value of −12.0 ppm at 1 Å and [2]^2−^ had a NICS value of −14.1 ppm at the C^3′^–C^6′^ bridge (Fig. S72‡).

The ACID plots of [9]^2−^ (Fig. 3) and [10]^2−^ (Fig. S69‡) also revealed global aromatic ring currents but showed that the current in the unsubstituted five-membered ring was significantly more pronounced than in the disubstituted ring. In fact, a closed loop of an additional local cyclopentadienide-like circuit in the unsubstituted five-membered ring was seen in each compound. The NICS *X* shift above the centre of the latter was 21% higher in [9]^2−^ and 13% higher in [10]^2−^ (−15.7 ppm for [9]^2−^ and −18.0 ppm for [10]^2−^) than those above the rings in unsubstituted [1]^2−^ (−20.8 ppm), indicating somewhat reduced aromatic character in the former two. NICS analysis further suggested significantly less pronounced aromaticity on the 1,3-diaryl substituted five-membered ring for both compounds, as seen in the less negative shifts compared to the unsubstituted five-membered ring, consistent with the ACID plots. The aromaticity calculations of [9]^2−^ also revealed that it behaved like a hybrid of [1]^2−^ and [2]^2−^. [9]^2−^ however had even higher NICS values than [2]^2−^ (see Table S3‡), which can be ascribed to the coplanar arrangement of the phenyl substituents with respect to the pentalenide core (0° dihedral twist compared to 30.2° in [2]^2−^) resulting in better conjugation with the core π system. Unlike in [2]^2−^, co-planarity is possible in [9]^2−^ due to the absence of substituents on the other half of the pentalenide, showing electronic preference for full conjugation where sterically possible. Experimentally this change in conjugation due to different dihedral angles between the pentalenide core and the aryl substituents could also be seen in the NMR shifts of the quaternary C^1^ and C^3^ atoms: whereas in [2]^2−^ they resonated at 109.5 ppm (30.2° twist angle) in [9]^2−^ they were shifted upfield to 103.5 ppm (Table S1‡).

The ACID plot of [12]^2−^ (Fig. 4) showed a relatively uniform aromatic ring current around the pentalenide perimeter similar to that of [2]^2−^ (*cf.*Fig. 1). The plot of the NICS *Z*-scans of [12]^2−^ also showed little difference in aromaticity between the two subunits, while the *X* scan indicated a slightly stronger aromatic circuit in the Me,Ph-substituted ring (−12.8 ppm) as shown by lower NICS shifts than in the Ph_2_-substituted ring (−10.9 ppm; Fig. S70 and Table S3‡). All three phenyl twist angles in [12]^2−^ fell in the range of 30–34°, showing a methyl group to cause the same level of partially interrupted conjugation as in the tetra-aryl pentalenides [2]^2−^ and [3]^2−^.

**Fig. 5:**
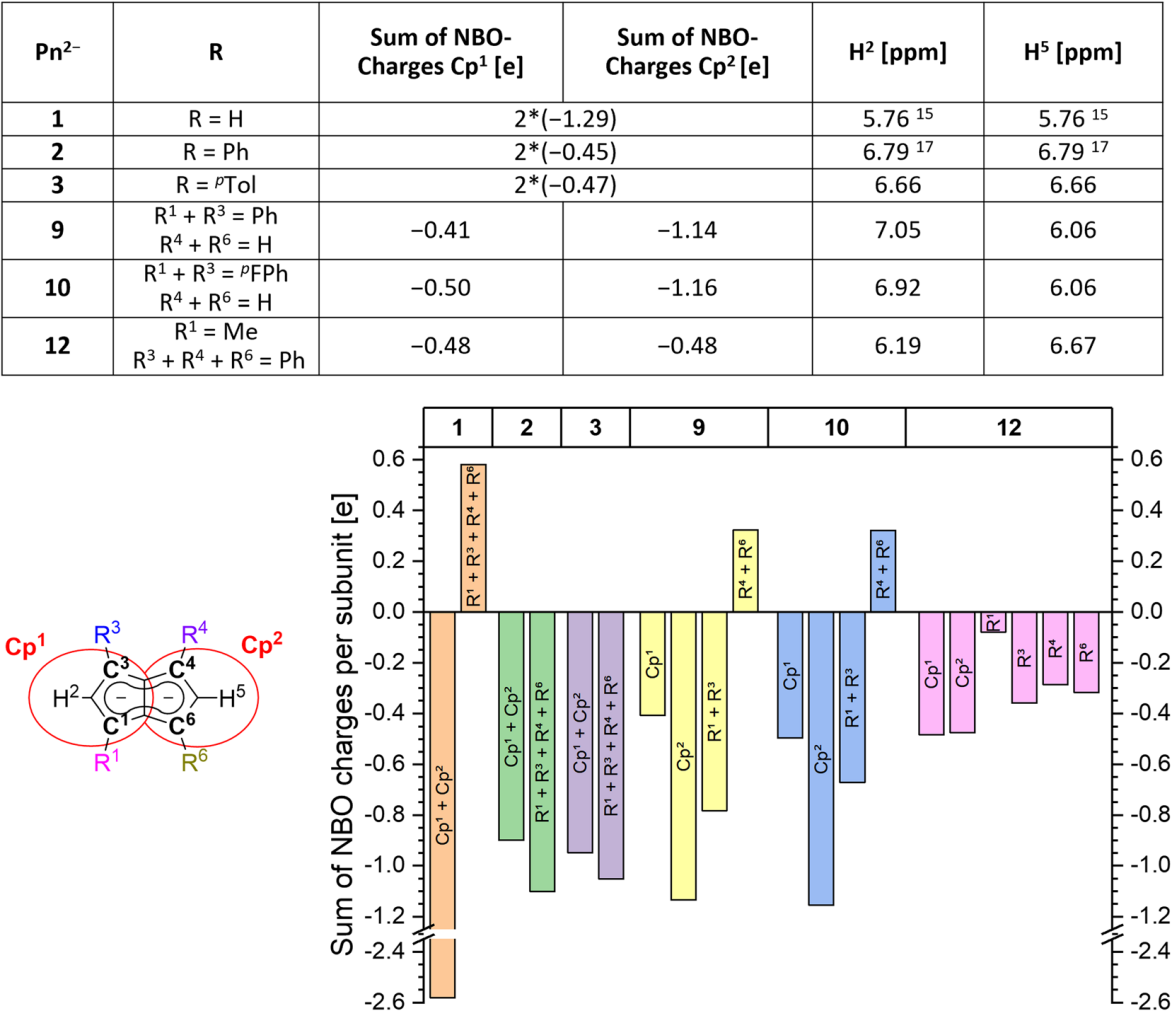
Left: separation of the pentalenide core into two Cp subunits (indicated by red circles) and calculated sums of NBO charges for each subunit compared to the experimentally obtained ^1^H NMR shifts. Right: plot of sums of NBO charges for each part (charges on the shared C^3^′ and C^6^′ atoms were equally distributed between both subunits; for compounds [1–3]^2−^ the charges on **Cp**^**1**^ and **Cp**^**2**^ are added up to eliminate artifacts from minor symmetry deviations).

In order to understand the degree of charge localisation and substituent effects in these pentalenides, NBO (natural bond orbital) calculations of [1]^2−^, [2]^2−^, [3]^2−^, [9]^2−^, [10]^2−^, and [12]^2−^ were carried out. For this analysis we formally separated the pentalenide core into two five-membered subunits with individual substituents (**Cp**^**1**^ and **Cp**^**2**^; Fig. 5, left) to see if they correlate with the experimentally observed relative ^1^H NMR chemical shifts of the wingtip protons H^2^ and H^5^ (Table S3‡ and Fig. 5).^36–41^

While in [1]^2−^ the dianionic charge was delocalised across but confined within the pentalenide core, in [2]^2−^ and [3]^2−^ more than half of the overall negative charge was delocalised into the four aryl substituents. This correlated with a 1 ppm downfield shift of the two equivalent wingtip protons in [2]^2−^ (6.79 ppm) and [3]^2−^ (6.66 ppm) compared to those of [1]^2−^ (5.76 ppm). The slightly higher shielding of the wingtip protons of [3]^2−^ with respect to those of [2]^2−^ can be ascribed to the +I effect of the *p*-tolyl groups leading to slightly less effective charge delocalisation in this case. This was also reflected in the comparison of the NBO charges on the two different aryl moieties of [2]^2−^ and [3]^2−^, where the phenyl groups accepted slightly more charge density than the tolyl groups (−1.10 e for [2]^2−^*vs.* −1.05 e for [3]^2−^). The same analysis allowed understanding the effect of unsymmetrical substitution patterns. In the pentalenides [9]^2−^ and [10]^2−^ the subunits without aryl groups in the 4,6 positions showed a higher sum of charges than their phenylated or ^*p*^FPh-substituted counterparts where the negative charge can be effectively delocalised into these substituents. This correlated with a highfield shift in the H^5^ proton (both 6.06 ppm for [9]^2−^ and [10]^2−^) while the H^2^ protons on the substituted five-membered rings were found at 7.05 ppm for [9]^2−^ and at 6.92 ppm for [10]^2−^, respectively. The slightly more shielded H^2^ in the latter compared to the former can be ascribed to the +M effect of the fluorine atoms in *para*-position of the ^*p*^FPh substituents; hence, the π-systems of the ^*p*^FPh-groups in [9]^2−^ accommodate less charge than the phenyl groups in [10]^2−^, resulting in a higher charge density in its pentalenide subunit. Comparing [9]^2−^ with [2]^2−^, the ^1^H NMR shifts of H^2^ in [9]^2−^ were even more deshielded than in [2]^2−^ which can be ascribed to the above-mentioned coplanar arrangement of the phenyl substituents with respect to the pentalenide core (0° dihedral twist compared to 30.2° for [2]^2−^) that results in better π conjugation and thus more effective charge distribution. This was also reflected in the absolute charge values of the aryl substituents, where one phenyl group in [2]^2−^ accepted a charge of −0.28 e while in [9]^2−^ it accepted −0.39 e.

Changes in the polarisation of the same subunit within different pentalenides can also be explained by the NBO analyses. For example, the observed NMR shifts of the wingtip protons in the unsubstituted parts of [9]^2−^ and [10]^2−^ were shifted slightly downfield from those in [1]^2−^. This is due to the fused nature of the two (hypothetical) subunits, since some charge density was transferred from the unsubstituted **Cp**^**1**^ into the arylated **Cp**^**2**^ where it may be delocalised into the electron-withdrawing substituents. The NBO charges also reflected the electronic influence of the alkyl substituent in [12]^2−^ well, as the C^1^ carbon was markedly less negatively charged than the C^3^ and C^4^ carbon atoms due the +I effect of the methyl group (Fig. 5). As expected, the methyl substituent itself did not accept a significant amount of charge from the pentalenide core, but its presence in 1-position led to a slightly higher charge density in the 3-phenyl group on the same ring compared to the two phenyls on the other subunit. Due to the electron-donating influence of the methyl group **Cp**^**1**^ showed a higher charge density than **Cp**^**2**^, consistent with its wingtip proton resonating more highfield (6.19 ppm) from that of the diphenyl substituted subunit (6.67 ppm). Due to the interaction of the fused five-membered rings this polarisation caused the H^5^ chemical shift to move upfield compared to that of [2]^2−^.

**Fig. 6:**
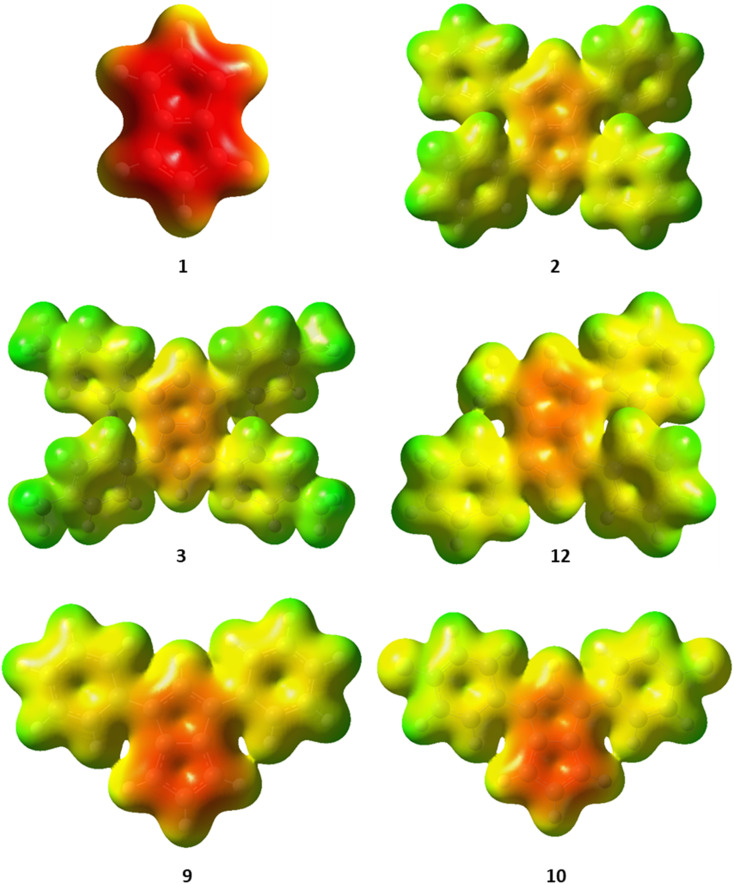
Electrostatic potential (ESP) maps for [1–3]^2−^, [9–10]^2−^ and [12]^2−^. Contour colours range from −0.36 (red) to 0.2 (blue) at an isovalue of 0.01.

The charge distribution within these pentalenides can be visualised by electrostatic potential (ESP) maps (Fig. 6) which illustrate the differences between unsubstituted [1]^2−^, disubstituted [9]^2−^ and [10]^2−^, and tetrasubstituted [2]^2−^, [3]^2−^, and [12]^2−^. As indicated by the NBO charge values (Fig. 5), the ESP maps of [1]^2−^, [2]^2−^, and [3]^2−^ showed a symmetrical charge distribution across the dianionic molecules, while [1]^2−^ has by far the highest charge density in the pentalenide core. Compounds [2]^2−^ and [3]^2−^ showed the least amount of charge density in the pentalenide core, and the negative charge on the core increased going to [12]^2−^, [9]^2−^ and [10]^2−^. In the latter two compounds the charge density was clearly more located on the unsubstituted subunit (similar to [1]^2−^) as indicated by the corresponding NBO values discussed above.

**Fig. 7:**
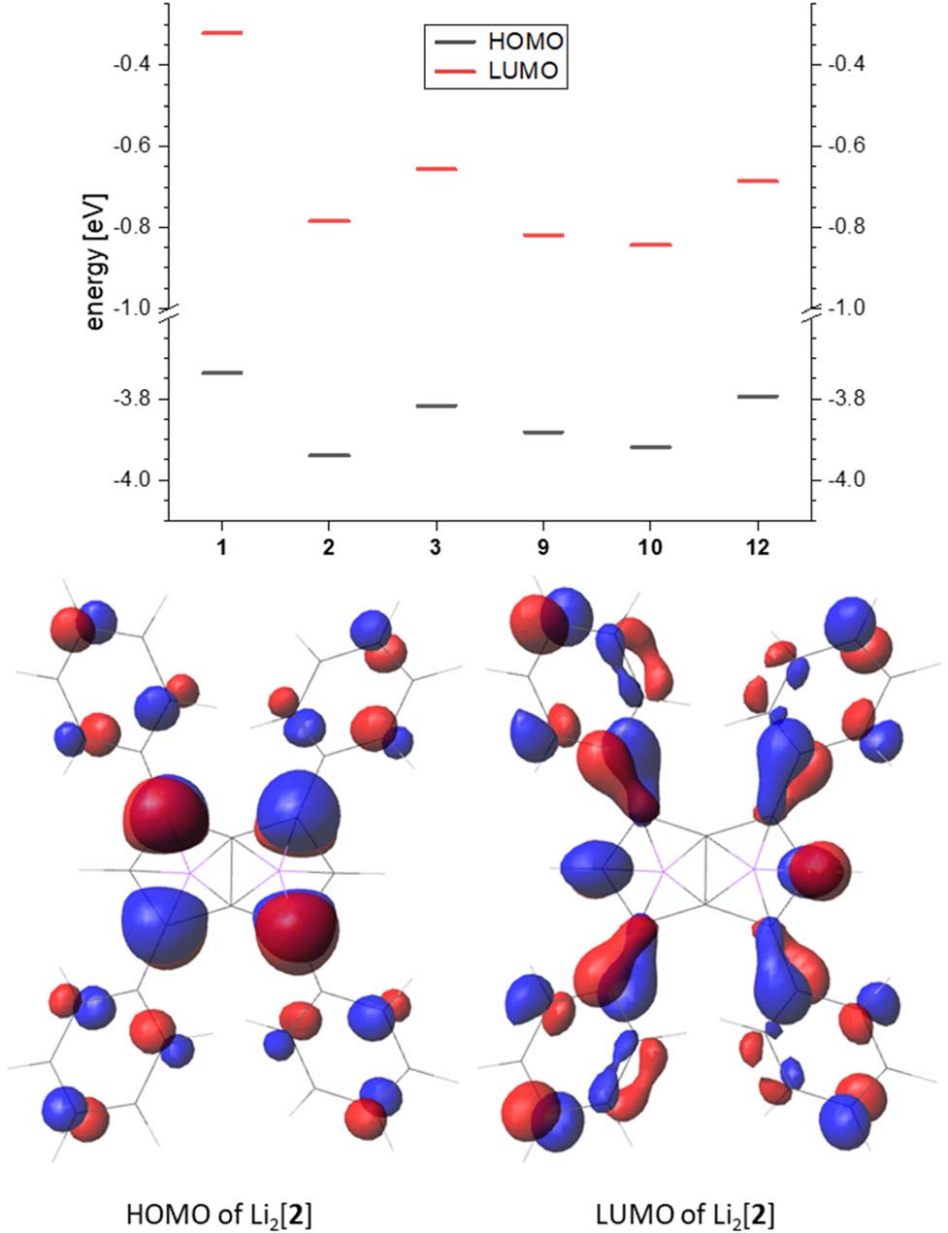
Top: frontier orbital energy levels of the calculated pentalenides in eV (B3LYP/6-311+G*, PCM(THF)). Bottom: representative frontier orbitals (isovalue 0.035).

Comparing the frontier orbitals and their energies across the series further illustrates the electronic influence of the substituents on the pentalenide (Fig. 7, top). In the HOMO of each compound investigated the largest contribution was found in the 1,3,4,6 position, and in the arylated systems some delocalisation into the aryl substituents could be seen (Fig. 7, bottom). The corresponding LUMO showed even stronger delocalisation into the aryl moieties. For the unsubstituted [1]^2−^ the HOMO looked similar to the substituted pentalenides, whilst the LUMO of [1]^2−^ was more located on the lithium cations (Fig. S74‡). The LUMO+12 of [1]^2−^ had a shape similar to the LUMOs of the substituted pentalenides, which is due to the missing distribution of electron density on aryl substituents, therefore destabilising this orbital. Although all substituted pentalenides investigated possessed a similar HOMO–LUMO gap of 3.1–3.5 eV, comparison of the absolute energy levels of unsubstituted [1]^2−^*versus* the tetraphenyl-substituted [2]^2−^ showed how the aryl moieties stabilise the frontier orbitals through conjugation with the dianionic core (Fig. 7, top; Table S2‡). Moving from the Ph_4_ substitution pattern in [2]^2−^ to Tol_4_ in [3]^2−^ slightly raised the energy levels due to the +I effect of the ^*p*^Me groups. The Ph_2_-substituted [9]^2−^ and the ^*p*^FPh_2_-substituted [10]^2−^ had a slightly higher HOMO energy than [2]^2−^, resulting in reduced HOMO–LUMO gaps (3.06 eV for [9]^2−^ and 3.08 eV for [10]^2−^). Comparing the raised frontier orbital energies of Ph_3_Me-substituted [12]^2−^ with those of Ph_4_-substituted [2]^2−^ again demonstrates the +I effect of the Me group.

### d. Transmetalation

**Fig. 8:**
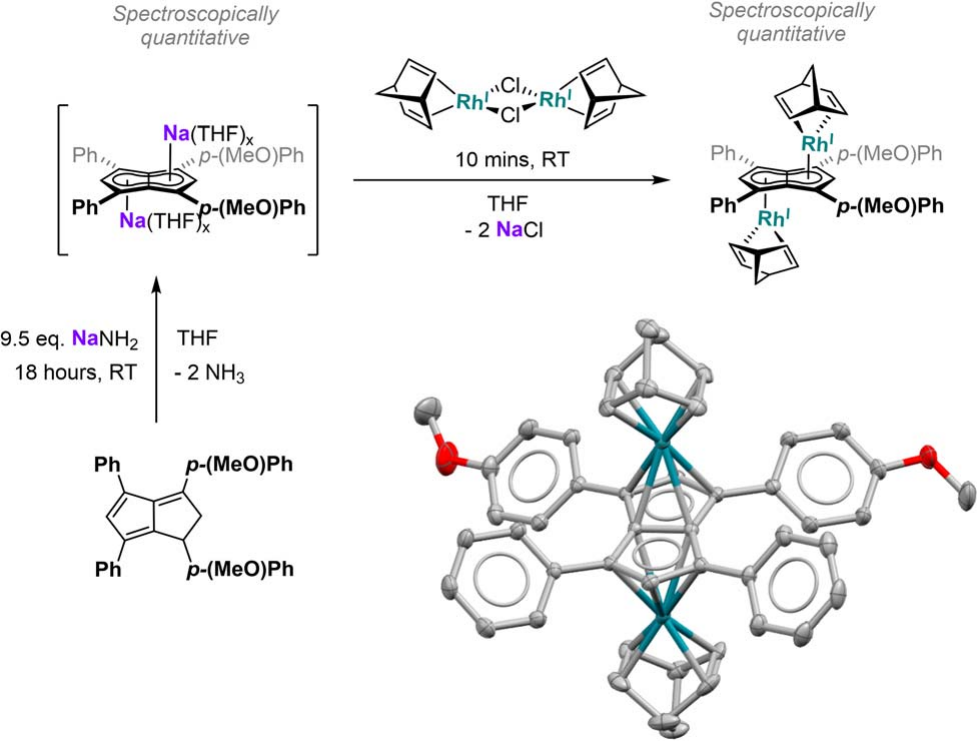
Top: one-pot-synthesis of *anti*-[Rh^I^(NBD)]_2_[μ:η^5^:η^5^(^*p*^MeOPh)_2_Ph_2_Pn] ([Rh(NBD)]_2_[7]). Bottom: single crystal X-ray structure of [Rh(NBD)]_2_[7] with thermal ellipsoids at the 50% probability level (hydrogen atoms omitted for clarity).

Since the results discussed above showed a significant influence of the substituent pattern on the polarisation of the pentalenide core, we sought to investigate their impact on polarising a homobimetallic transition metal complex. As a proof of concept, we decided to try transmetalation of the unsymmetrically substituted [7]^2−^ with the symmetrical [Rh^I^(NBD)(μ-Cl)]_2_ dimer in THF. These conditions have recently been shown to lead to formation of *anti*-homobimetallic complexes of [2]^2−^ with both group 1 and group 2 pentalenide precursors.^42^ However, using Li_2_[7] with [Rh^I^(NBD)(μ-Cl)]_2_ in THF at room temperature resulted in a complex mixture of unidentified products including several pentalenide species. Using the heavier alkali metal analogue Na_2_[7] (prepared *in situ* from 7H_2_ with an excess of NaNH_2_) under the same conditions cleanly yielded *anti*-[Rh^I^(NBD)]_2_[μ:η^5^:η^5^**(**^***p***^**MeOPh)**_**2**_**Ph**_**2**_**Pn**] ([Rh(NBD)]_2_[7]) within 10 minutes in spectroscopically quantitative yield (Fig. 8; see Chapter 3.13 and Fig. S51–S52 in the ESI‡).

The ^1^H NMR spectra of [Rh(NBD)]_2_[7] displayed a reduced wingtip shift difference of 0.1 ppm in comparison to Li_2_[7] (0.18 ppm), likely as a result of the change from a SSIP to a π complex. The latter was clearly established by characteristic ^103^Rh–^1^H-couplings (^2^*J*_RhH_ = 0.9 Hz for H^2^ and 0.8 Hz for H^5^) as well as ^103^Rh–^13^C-couplings (^1^*J*_RhC_ = 6.3 Hz for C^2^ and 5.8 Hz for C^5^). The NBD ligands gave rise to two doublets for the olefinic carbons at 41.3 ppm and 41.2 ppm respectively (see Fig. S53‡), again showing the asymmetry of the two [Rh^I^(NBD)]^+^ fragments bound to [7]^2−^. The observation of distinct resonances and coupling constants also indicated a static bonding situation without dynamic exchange (*via* decoordination or ring-walking)^33^ of the two metals. [Rh(NBD)]_2_[7] was further characterised by mass spectrometry and XRD analysis, the latter confirming the expected *anti*-arrangement of the two [Rh(NBD)] fragments^42^ (see Fig. 8 and Chapter 8.5 in the ESI‡). In the solid state both C_5_-centroid to Rh distances were 1.93 Å, very similar to those reported for [Rh(NBD)]_2_[2] (1.94 Å).^42^

## 3. Conclusion

We have described the synthesis and properties of nine new symmetrically and unsymmetrically arylated pentalenides, including a strategy for the introduction of alkyl substituents with β-hydrogens that otherwise lead to exocyclic deprotonation. The dialkali metal salts crystallise in the usual *trans* η^5^ coordination mode, but in ethereal solution solvent-separated ion pairs exist that feature rapid (likely concerted) substituent flipping. DFT calculations have confirmed a dihedral twist angle of ∼30° (as found experimentally in the solid state) as the energetic minimum due to steric clash competing with the electronic preference for co-planar conjugation. Even at a ∼30° twist, four aryl substituents have been shown to withdraw over 50% of the charge density from the core and reduce its aromaticity by up to 20%. The NMR chemical shifts of the wingtip protons in the 2 and 5 positions of the pentalenide serve as sensitive probes for the polarisation, charge density, and degree of aromaticity of the two five-membered subunits, and the ^13^C NMR shifts of the quaternary carbons connected to the aryl substituents are related to the dihedral angle and degree of conjugation. Whereas unsubstituted C_8_H_6_^2−^ is best described as a fully conjugated sp^2^ system containing 10 π electrons around its perimeter (with negligible electron density at its transannular bond), when aryls are introduced in the 1,3,4,6 positions conjugation with the substituents leads to charge localisations in these pentalenides that are more accurately depicted as two allyl units joined by a shared C=C bond. Frontier orbital analysis has shown such arylated pentalenides to be slightly weaker donors (*i.e.* less reducing) but much better acceptor ligands than unsubstituted (and likely also alkylated) pentalenides, making them promising “soft” ligands for electron-rich metal centres. As a proof of concept, we provide an example of transmetalation of an unsymmetrically substituted pentalenide to a d-block element and report a polarised dirhodium(i) complex where each metal atom with its auxiliary ligands are electronically as well as sterically distinct. The ease and modularity of our synthetic protocol paired with the quantum chemical insights reported will hopefully enable more widespread utilisation of this ligand framework and finally allow its full potential in organometallic chemistry to be realised. In particular, the possibility of designing electronically asymmetric pentalenide ligands offers exciting prospects for the controlled synthesis of homo- and heterobimetallic complexes for application in sensing, functional materials and catalysis.^9–12^ Further variations in substitution patterns, including multiple alkyl groups introduced *via* complementary strategies, as well as their use in organometallic chemistry will be reported in due course.

## Data availability

The datasets supporting this article have been uploaded as part of the ESI.‡

## Author contributions

NAJ carried out most experimental and analytical work, except for the exocyclic deprotonation reactions of SMB and the transmetalation work of HJS and MK. The computational work was largely performed by AH with guidance by HH. SBR and NAJ were responsible for collecting mass spectrometric data. GKK carried out the XRD analyses. The manuscript was prepared by NAJ and AH, and edited and refined by HH and UH who led the project.

## Conflicts of interest

There are no conflicts to declare.

## Supplementary Material

SC-015-D3SC04622B-s001

SC-015-D3SC04622B-s002
