# Supplementary material for: Understanding and tuning the electronic structure of pentalenides
Source: Chem Sci. 2024 Jul 5;15(32):12765–79. doi: 10.1039/d3sc04622b (PMC11323301; doi:10.1039/d3sc04622b)
Supplement: SC-015-D3SC04622B-s001 [file SC-015-D3SC04622B-s001.pdf]

Electronic Supplementary Material (ESI) for Chemical Science.
This journal is © The Royal Society of Chemistry 2024

Understanding and tuning the electronic structure of pentalenides

Niko A. Jenek,[a] Andreas Helbig,[b] Stuart M. Boyt,[a] Mandeep Kaur,[a] Hugh J. Sanderson,[a] Shaun B. Reeksting,[c] Gabriele Kociok-Köhn,[c] Holger Helten*[b] and Ulrich Hintermair*[a]

a) *Department of Chemistry, University of Bath, Claverton Down, Bath BA2 7AY, UK.*

b) *Institute of Inorganic Chemistry and Institute for Sustainable Chemistry & Catalysis with Boron (ICB), Julius-Maximilians-University Würzburg, Am Hubland, D-97074 Würzburg, Germany.*

c) *Chemical Characterisation Facility, University of Bath, Claverton Down, Bath BA2 7AY, UK.*

holger.helten@uni-wuerzburg.de

u.hintermair@bath.ac.uk

SUPPLEMENTARY INFORMATION

Table of contents

1. General ... 3

2. Synthesis and characterisation data of novel starting materials ... 4

3. Synthesis and characterisation data of products ... 8

3.1 Dilithium 1,3,4,6-tetra-p-tolylpentalenide (Li_2[**3**]) ... 8

3.2 Dilithium 1,3,4,6-tetrakis(3,5-dimethylphenyl)pentalenide (Li_2[**4**]) ... 9

3.3 Dilithium 1,3-diphenyl-4,6-di-p-tolylpentalenide (Li_2[**5**]) ... 10

3.4 Dilithium 1,3-bis(3,5-dimethylphenyl)-4,6-diphenylpentalenide (Li_2[**6**]) ... 11

3.5 Dilithium 1,3-bis(4-methoxyphenyl)-4,6-diphenylpentalenide (Li_2[**7**]) ... 12

3.6 Lithium potassium 1,3-bis(4-fluorophenyl)-4,6-di-p-tolylpentalenide (LiK[**8**]) ... 13

3.7 Dilithium 1,3-diphenylpentalenide (Li_2[**9**]) ... 14

3.8. Dilithium 1,3-di(4-fluorophenyl)pentalenide (Li_2[**10**]) ... 15

3.9. Deprotonation product of 1,3,8-triphenyl-4,5,6,7,7a,8-hexahydrocyclopenta-[a]-indene and $LiNEt_2$ (Li[**11-exo**]) ... 16

3.10. Lithium 3-vinyl-1,4,6-triphenyl-1,2,2-trihydropentalenide (Li[**12-exo**]) ... 17

3.11. Dilithium 1-methyl-3,4,6-triphenylpentalenide (Li_2[**12**]) ... 18

3.12. Overview wingtip ^{1}H and *ipso* ^{13}C chemical shifts ... 19

3.13. *anti*-bis(2,5-Norbornadiene)dirhodium 1,3-bis(4-methoxyphenyl)-4,6-diphenyl-pentalenide (Rh_2NBD_2[**7**]) ... 20

4. NMR spectra of novel starting materials (Figures S1–S10) ... 21

5. NMR spectra of products (Figures S11–S53) ... 26

6. Variable temperature NMR of potassium lithium 1,3,4,6-tetrakis(3,5-dimethylphenyl)-pentalenide KLi[**4**] (Figures S54–S57) ... 48

7. NMR spectra of deprotonative metalation attempt of CF_3Ph-substituted dihydropentalenes (Figures S58 and S59) ... 50

8. X-ray crystallography (Figures S60–S64) ... 52

8.1. Crystal data and structure refinement for disodium 1,3,4,6-*para*-tolylpentalenide (Na_2[**3**]) 53

8.2. Crystal data and structure refinement for 1-methyl-3,4,6-triphenyl-1,2-dihydropentalene (**12'**H_2)55

8.3. Crystal data and structure refinement for 3-methyl-1,4,6-triphenyl-1,2-dihydropentalene (**12**H_2) 57

8.4. Crystal data and structure refinement for 1,3,8-triphenyl-4,5,6,7,7a,8-hexahydro-cyclopenta-[a]-indene (**11**H_2) ... 59

8.5. Crystal data and structure refinement for *anti*-bis(2,5-Norbornadiene)dirhodium 1,3-bis(4-methoxyphenyl)-4,6-diphenyl-pentalenide (Rh_2NBD_2[**7**]) ... 61

9. Calculations (Figures S65–S79) ... 63

1. General

Commercially available materials were obtained from Fluorochem, Sigma Aldrich, Alfa Aesar, Fisher or Acros. All manipulations were carried out under dry argon using standard Schlenk techniques or using a MBraun Unilab Plus glovebox, unless specified otherwise. NMR spectroscopy was conducted using a 400 MHz Bruker Avance III or 500 MHz Bruker Avance III at 25 °C. Chemical shifts (δ) are reported in ppm relative to the residual proton chemical shifts of the proteo- or deuterated solvent used (^{1}H: 7.26 for $CDCl_3$, 5.32 for CD_2Cl_2, 3.62/1.78 for THF-h_8 and 3.58/1.72 for THF-d_8; and ^{13}C{^{1}H}: 77.16 for $CDCl_3$, 53.84 for CD_2Cl_2, 68.03/26.19 for THF-h_8 and 67.21/25.31 for THF-d_8) and relative to external LiCl (^{7}Li) as well as $BF_3{\cdot}Et_2O$ (^{19}F{^{1}H}). Multiplicities in NMR are reported as s (singlet), d (doublet), t (triplet), q (quartet), m (multiplet), *pseudo*-t (*pseudo*-triplet), dd (doublet of doublets) and bs (broad singlet). Mass spectrometry (Agilent 6545 QTOF (ESI) or Bruker MaXis HD QTOF (APCI)) was carried out at the Material and Chemical Characterisation Facility at the University of Bath. Tetrahydrofuran was dried by distillation from potassium.

Literature known substituted 1,2-dihydropentalenes were synthesised according to our previously reported protocol.[1] 1,4-Diphenylcyclopenta-1,3-diene and 1-phenyl-4-tolylcyclopenta-1,3-diene were synthesised via our modification of the protocol presented by Drake and Adams.[1-3] 1,3-Bis(3,5-bis(trifluoromethyl)phenyl)-2-propen-1-one was synthesised according to Hicks *et al*.[4] The diphenyl-dihydropentalene isomer mixture was obtained following Griesbeck's protocol.[5] [Rh(NBD)(μ-Cl)]$_2$ was synthesised according to literature.[6]

2. Synthesis and characterisation data of novel starting materials

1-methyl-3,4,6-triphenyl-1,2-dihydropentalene (12'H_2)

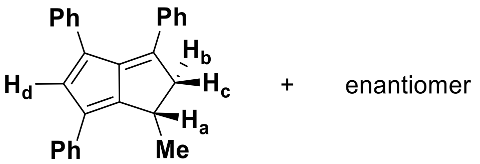

Following our previously reported protocols,[1, 3] 1,4-diphenylcyclopenta-1,3-diene (606 mg, 2.77 mmol, 1 eq.) and technical grade (*E*)-1-phenylbut-2-en-1-one (80% pure, 721 mg, 3.94 mmol, 1.4 eq.) were dissolved in 12 mL dry methanol as well as 12 mL dry toluene under stirring at room temperature in a Schlenk Cajon flask. Pyrrolidine (529 mg, 7.43 mmol, 2.7 eq.) was added dropwise over a range of 6 minutes, the reaction vessel was sealed, and the resulting solution was stirred for 49 hours at 75 °C. After cooling to room temperature, to the dark red solution was added commercial glacial acetic acid (2 mL) in air and the solution stirred for 5 minutes. The solvent was removed under reduced pressure and the crude material was dissolved in diethyl ether (125 mL) as well as aqueous Na_2CO_3 (125 mL). The organic phase was washed with water (2·100 mL) and brine (100 mL). The solvent of the ether fraction was removed under reduced pressure and the crude dissolved in a minimum of 1:1 diethyl ether/*n*-hexane, followed by drying-filtering through neutral silica using 1:1 *n*-hexane:diethyl ether as the eluent, collecting the first dark red-orange band only. This fraction was further purified via repeated recrystallisation from boiling acetonitrile (3 mL) in 16% yield (155 mg, 0.45 mmol). Melting point: 184 °C. Crystals suitable for XRD analysis were grown by slow evaporation of a diethyl ether:$CDCl_3$ solution at room temperature.

^{1}H NMR (400 MHz, $CDCl_3$): δ = 7.65–7.60 (m, 2H, Ph*H*), 7.42–7.37 (m, 2H, Ph*H*), 7.30–7.26 (m, 2H, Ph*H*), 7.26–7.20 (m, 2H, H_d and Ph*H*), 7.18–7.09 (m, 7H, Ph*H*), 3.89 (dd, $^{3}J_{HH}$ = 6.1 Hz, $^{2}J_{HH}$ = 18.9 Hz, 1H, H_c), 3.67–3.59 (m, 1H, H_a), 3.09 (d, $^{2}J_{HH}$ = 18.9 Hz, 1H, H_b), 1.35 (d, $^{2}J_{HH}$ = 7 Hz, 3H, Me).

^{13}C{^{1}H} NMR (101 MHz, $CDCl_3$): δ = 155.3, 152.2, 145.6, 139.6, 136.8, 136.0, 135.7, 130.0, 129.4, 129.0, 128.7, 128.4, 127.98, 127.95, 126.4, 126.2, 126.1, 53.0, 31.4, 19.6.

HR ESI-MS (+): m/z expected for $[M+H]^+$ = 347.1794; found = 347.1781.

Note: Crystals suitable for XRD analysis of the other regioisomer, 3-methyl-1,4,6-triphenyl-1,2-dihydropentalene, were grown by slow evaporation of a n-hexane:CH_2Cl_2 solution at room temperature.

1,3-diphenyl-4,6-bis(3,5-bis(trifluoromethyl)phenyl)-1,2-dihydropentalene (8d-H_2)

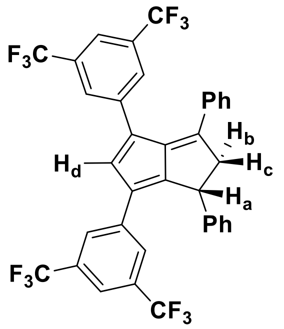

Using the NaO*t*Bu-promoted method described in our previous work,[1] to a pale-yellow stirred solution of 1,4-diphenylcyclopenta-1,3-diene (90 mg, 0.41 mmol, 1 eq.) in 5.2 mL dry THF inside a glovebox, a solution of sodium *tert*-butoxide (40 mg, 0.41 mmol, 1 eq.) in 2.6 mL dry THF was added dropwise over a range of six minutes. After 45 minutes of stirring, to this now golden-yellow solution was added a solution of 1,3-bis(3,5-bis(trifluoromethyl)-phenyl)-2-propen-1-one[4] (298 mg, 0.62 mmol, 1.51 eq.) in 8 mL dry THF dropwise over a range of 37 minutes. The resulting solution was transferred into a Cajon Schlenk flask, the flask sealed, taken out of the glove box, and stirred for further 19 hours at room temperature, followed by stirring at 75 °C for 19 hours. After cooling down to room temperature, the mixture was quenched with 0.1 mL $NH_4Cl_{aq.sat.}$ and stirred fur further 30 minutes, followed by a dilution with 30 mL diethyl ether and 30 mL water. The organic phase was washed with 2·30 mL water and 2·30 mL brine. The solvent was removed under reduced pressure and the fraction was then redissolved in a minimum of 3:1 hexane/diethyl ether and filtered through silica using 3:1 hexane/diethyl ether as the eluent, collecting the first broad dark red-violet band only. This fraction was further purified *via* double preparative thin layer chromatography (10:1 and 20:1 cyclohexane/toluene as eluent), giving the desired 1,2-dihydropentalene as cherry-red solid (123 mg, 0.18 mmol, 44%). Melting point: 89–90 °C. $R_f \approx 0.58$ (10:1 cyclohexane:toluene).

^{1}H NMR (500 MHz, $CDCl_3$): δ = 7.77 (s, 2H, Ar_FH), 7.64-7.61 (two overlapping s, 3H, Ar_FH), 7.57 (s, 1H, Ar_FH), 7.48 (s, 1H, H_d), 7.37–7.33 (m, 1H, Ar*H*), 7.32–7.24 (m, 7H, Ar*H*), 7.23-7.19 (m, 2H, Ar*H*), 4.62 (dd, $^3J_{HH}$ = 6.5 Hz, $^3J_{HH}$ = 1.8 Hz, 1H, H_a), 4.21 (dd, $^2J_{HH}$ = 19.6 Hz, $^3J_{HH}$ = 6.5 Hz, 1H, H_c), 3.57 (d, $^2J_{HH}$ = 19.6 Hz, 1H, H_b).

^{13}C{^{1}H} NMR (126 MHz, $CDCl_3$): δ = 160.5, 153.9, 145.3, 142.4, 139.8, 137.7, 136.5, 134.4, 131.8, 131.5, 131.3, 130.9, 129.7, 129.3, 128.1, 127.85, 127.77, 127.5, 127.3, 126.1, 123.5 (q, $^1J_{CF}$ = 273.0 Hz, CF_3 via HMBC), 123.4 (q, $^1J_{CF}$ = 273.0 Hz, CF_3 via HMBC), 119.8, 119.7, 55.7, 43.5.

^{19}F{^{1}H} NMR (471 MHz, $CDCl_3$): δ = −63.0, −63.1 ppm.

HR ESI-MS (−): m/z expected for $[M-H]^-$ = 679.1300; found = 679.1301.

1,3-bis(4-fluorophenyl)-1,2-dihydropentalene/4,6-bis(4-fluorophenyl)-1,5-dihydropentalene (10H$_2$)

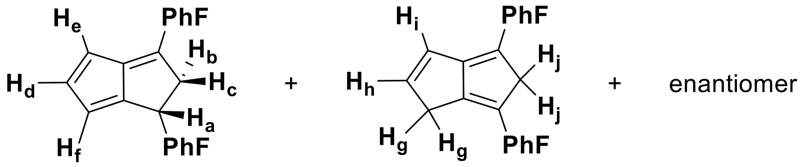

Using a modification of the protocol reported by Griesbeck,[5] commercially available 4,4'-difluorochalcone (289 mg, 1.18 mmol, 1 eq.) was provided in a Schlenk flask and dissolved in 1.5 mL dry methanol while stirring. The flask was cooled down to 0 °C, followed by the addition of freshly cracked cyclopentadiene (350 mg, 5.29 mmol, 4.5 eq.). Pyrrolidine (370 mg, 5.20 mmol, 4.4 eq.) was added dropwise over a range of 15 minutes to the stirring mixture. The flask was sealed, and the resulting solution was stirred for 21.75 hours while warming up to room temperature. Commercial glacial acetic acid (2 mL) was added in air and the solution stirred for 5 minutes. The solvent was removed under reduced pressure and the crude material was dissolved in diethyl ether (10 mL) as well as water (10 mL). The organic phase was washed with water (2·5 mL) and brine (7.5 mL). The solvent of the ether fraction was removed under reduced pressure and the crude dissolved in a minimum of diethyl ether, followed by drying-filtering through neutral silica using 2:1 *n*-hexane:diethyl ether as the eluent, collecting the first bright orange band only. This fraction was further purified via preparative thin layer chromatography (5:1 cyclohexane:toluene), giving a corresponding 1,2- and 1,5-dihydropentalene isomer mixture (1:0.6 ratio) as bright orange amorphous solid (25 mg, $8.55 \cdot 10^{-5}$ mol, 7% combined yield). $R_f \approx 0.45$ (5:1 cyclohexane:toluene).

^{1}H NMR (500 MHz, CD_2Cl_2): δ = 7.84–7.78 (m, 2H, Ar*H* of 1,2-isomer), 7.56–7.46 (2 m, 4H, Ar*H* of 1,5-isomer), 7.27–6.97 (overlapping series of m, ≥10H, Ar*H* of both isomers), 6.95–6.92 (m, 1H, H_d), 6.91–6.87 (m, 1H, H_i), 6.71–6.67 (m, 1H, H_h), 6.51 (d, $^{3}J_{HH}$ = 5.1 Hz, 1H, H_e), 5.89 (s, 1H, H_f), 4.32–4.27 (m, 1H, H_a), 4.07 (s, 2H, H_j), 4.00 (dd, $^{2}J_{HH}$ = 18.6 Hz, $^{3}J_{HH}$ = 6.8 Hz, 1H, H_c), 3.39 (s, 2H, H_g), 3.31 (d, $^{2}J_{HH}$ = 18.6 Hz, 1H, H_b).

^{13}C{^{1}H} NMR (126 MHz, CD_2Cl_2): δ = 164.2 (d, $^{1}J_{CF}$ = 250.0 Hz, *C*F), 161.9 (d, $^{1}J_{CF}$ = 244.0 Hz, *C*F), 161.6 (d, $^{1}J_{CF}$ = 245.0 Hz, *C*F), 161.5 (d, $^{1}J_{CF}$ = 246.0 Hz, *C*F), 155.2, 151.4, 150.4, 147.2, 147.0, 143.5, 142.7, 140.90, 140.88, 133.5, 133.0, 132.2, 132.1, 131.8, 131.54, 131.47, 130.6, 129.14, 129.08, 127.5, 127.4, 127.3, 126.93, 126.87, 116.3 (d, $^{2}J_{CF}$ = 22.0 Hz, CH=*C*H-CF), 115.9 (d, $^{2}J_{CF}$ = 22.0 Hz, CH=*C*H-CF), 115.8 (d, $^{2}J_{CF}$ = 22.0 Hz, CH=*C*H-CF), 115.5 (d, $^{2}J_{CF}$ = 21.0 Hz, CH=*C*H-CF), 112.1, 52.3, 48.1, 41.4, 33.6.

^{19}F{^{1}H} NMR (471 MHz, CD_2Cl_2): δ = −110.3, −116.9, −117.0, −117.8.

HR ESI-MS (−): m/z expected for $[M-H]^-$ = 293.1136; found = 293.1128.

1,3,8-triphenyl-4,5,6,7,7a,8-hexahydrocyclopenta[a]indene (11H$_2$)

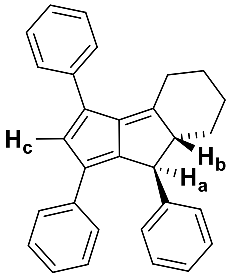

1,3,6-Triphenylfulvene (0.18 g, 0.59 mmol) and cyclohexanone (0.07 ml, 0.65 mmol) were dissolved in 5 ml methanol and 5 ml toluene under argon, and to this was added pyrrolidine (0.07 ml, 0.84 mmol) and the mixture stirred under previously reported reaction conditions for [6+2] cycloadditions.[3] The solvent was removed under reduced pressure. In air the crude material was dissolved in 75 mL diethyl ether and washed with 3·75 mL water and 75 mL brine. The ether fraction was dried over $MgSO_4$ and the solvent the solvent removed reduced pressure. The fraction was then filtered through silica using 1:1 diethyl ether/hexane as the eluent, and the solvent removed under reduced pressure. The resulting dark red solid was recrystallized from ethanol to give an orange red crystalline solid (9 mg, $2.33 \cdot 10^{-5}$ mol, 4%), which was suitable for XRD analysis.

^{1}H NMR (400 MHz, CD_2Cl_2): δ = 7.55–7.51 (m, 2H, Ph*H*), 7.43–7.34 (m, 4H, Ph*H*), 7.31 (s, 1H, H_c), 7.31–7.24 (m, 5H, Ph*H*), 7.20–7.14 (m, 3H, Ph*H*), 7.09–7.04 (m, 1H, Ph*H*), 3.99 (s, 1H, H_a), 3.22–3.14 (m, 1H, -CH_2-), 3.07–3.01 (m, 1H, H_b), 2.56–2.48 (m, 1H, -CH_2-), 2.36 (td, J_{HH} = 13.5 Hz, J_{HH} = 5.7 Hz, 1H, -CH_2-), 1.97–1.82 (m, 2H, -CH_2-), 1.53–1.44 (m, 2H, -CH_2-), 1.40–1.26 (m, 1H, -CH_2-).

^{13}C{^{1}H} NMR (101 MHz, CD_2Cl_2): δ = 164.0, 146.8, 144.3, 144.2, 137.3, 137.2, 135.8, 130.7, 129.1, 129.0, 128.7, 128.54, 128.45, 127.6, 126.70, 126.68, 126.6, 126.2, 67.3, 51.6, 35.3, 29.8, 27.5, 26.0.

HR ESI-MS (+): m/z expected for $[M+H]^+$ = 387.2108; found = 387.2104.

3. Synthesis and characterisation data of products

General procedures for the deprotonative metalation of substituted 1,2-dihydropentalenes (PnH_2):

Inside a glovebox, dry THF (volume specified in each case) was added to the respective base (amount specified in each case) at room temperature. A solution of the substituted **PnH_2** (amount specified in each case) in dry THF (volume specified in each case) was added dropwise over a range of three minutes. The resulting mixture stirred for 1–4 hours in total at room temperature (unless specified otherwise), after which it was transferred into a J. Youngs NMR tube. The ^{1}H NMR spectrum of the reaction solution showed in most of the cases quantitative conversion into the corresponding pentalenide species after 1–168 hours.

3.1 Dilithium 1,3,4,6-tetra-p-tolylpentalenide (Li_2[**3**])

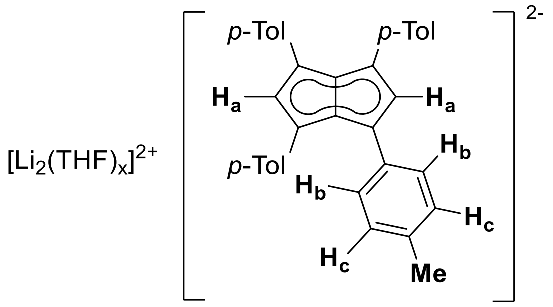

Reaction parameters: 5 eq. $LiNEt_2$ (0.25 mmol in 0.35 mL) and 1 eq. **$^{p}Tol_4PnH_2$** (0.05 mmol in 0.60 mL) used. Reaction volume in total: 0.95 mL. Quantitative conversion was achieved after one hour. The initial solubility was approximately 0.05 mmol **[$^{p}Tol_4Pn$]Li_2** in 0.95 mL THF at 20 °C. The solubility started to decrease after 24 hours. Further addition of solvent redissolved the precipitate.

^{1}H NMR (500 MHz, THF-h_8): δ = 7.06 (d, $^3J_{HH}$ = 7.9 Hz, 8H, H_b), 6.82 (d, $^3J_{HH}$ = 7.9 Hz, 8H, H_c), 6.66 (s, 2H, H_a), 2.24 (s, 12H, 4 CH_3).

^{13}C{^{1}H} NMR (126 MHz, THF-h_8): δ = 139.7, 127.8, 127.7, 127.2, 118.8 (via HSQC/HMBC), 113.1 (via HSQC), 106.4, 21.1.

^{7}Li NMR (194 MHz, THF-h_8): δ = 0.29, −3.80.

^{1}H DOSY (500 MHz, THF-h_8): D = 5.97·10^{-10} m^2s^{-1}.

HR APCI-MS (−): m/z expected for $[M-2Li]^{\bullet-}$ = 462.2353; found = 462.2311.

3.2 Dilithium 1,3,4,6-tetrakis(3,5-dimethylphenyl)pentalenide (Li_2[**4**])

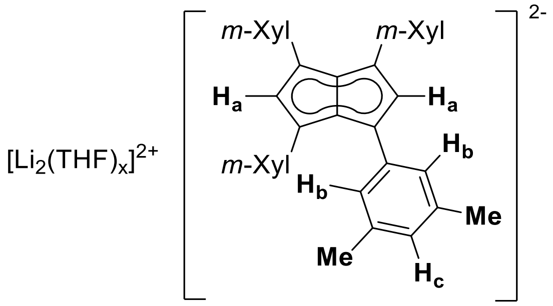

Reaction parameters: 5 eq. $LiNEt_2$ ($1.25\cdot10^{-4}$ mmol in 0.35 mL) and 1 eq. **$^{m}Xyl_4PnH_2$** isomer mixture ($2.50\cdot10^{-5}$ mol in 1 mL) used. Reaction volume in total: 1.35 mL. The reaction temperature had to be increased to 40 °C after the transfer into the J. Youngs NMR tube due to precipitation of product. Quantitative conversion after 3.5 hours (1.5 hours at 20 °C and 2 hours at 40 °C). The initial solubility was approximately $2.50\cdot10^{-5}$ mol **[$^{m}Xyl_4Pn$]Li_2** in 1.35 mL THF at 40 °C. Solubility did decrease after 24 hours.

^{1}H NMR (500 MHz, THF-h_8): δ = 6.76 (two overlapping s, 10H, H_a, H_b), 6.35 (s, 4H, H_c), 2.11 (s, 24H, 8 CH_3).

$^{13}C\{^{1}H\}$ NMR (126 MHz, THF-h_8): δ = 141.9, 135.2, 125.8, 121.1, 119.1 (via HMBC), 112.2 (via HSQC), 107.1, 21.6.

^{7}Li NMR (194 MHz, THF-h_8): δ = 0.69.

^{1}H DOSY (500 MHz, THF-h_8): D = $6.52\cdot10^{-10}$ m^2s^{-1}.

HR APCI-MS (−): m/z expected for $[M-2Li]^{\bullet-}$ = 518.2979 ; found = 518.2952.

3.3 Dilithium 1,3-diphenyl-4,6-di-p-tolylpentalenide (Li_2[**5**])

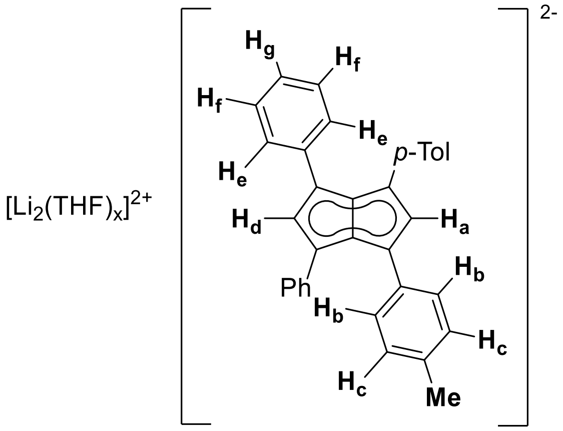

Reaction parameters: 5 eq. $LiNEt_2$ (0.42 mmol in 0.31 mL) and 1 eq. **$^{p}Tol_2Ph_2PnH_2$** isomer mixture (0.08 mmol in 0.61 mL) used. Reaction volume in total: 0.92 mL. Quantitative conversion was achieved after one hour. The initial solubility was approximately $8.00 \cdot 10^{-5}$ mol **[$^{p}Tol_2Ph_2Pn$]Li_2** in 0.92 mL THF at 20 °C. The solubility did not decrease after 24 hours.

^{1}H NMR (500 MHz, THF-h_8): δ = 7.14 (d, $^{3}J_{HH}$ = 7.7 Hz, 4H, H_e), 7.06 (d, $^{3}J_{HH}$ = 7.7 Hz, 4H, H_b), 6.97 (*pseudo*-t, $^{3}J_{HH}$ = 7.7 Hz, $^{3}J_{HH}$ = 7.7 Hz, 4H, H_f), 6.83 (d, $^{3}J_{HH}$ = 7.7 Hz, 4H, H_c), 6.72 (s, 1H, H_d), 6.66 (s, 1H, H_a), 6.63 (t, $^{3}J_{HH}$ = 7.7 Hz, 2H, H_g), 2.24 (s, 6H, 2 CH_3).

^{13}C{^{1}H} NMR (126 MHz, THF-h_8): δ = 142.3, 139.7, 127.81, 127.77, 127.6 (via HMBC), 127.4, 127.2, 127.0, 119.2, 113.5, 112.7, 107.2, 106.6, 21.0.

^{7}Li NMR (194 MHz, THF-h_8): δ = 0.62.

^{1}H DOSY (500 MHz, THF-h_8): D = $5.53 \cdot 10^{-10}$ m^2s^{-1}.

HR APCI-MS (−): m/z expected for $[M{-}2Li]^{\bullet-}$ = 434.2040; found = 434.2010.

3.4 Dilithium 1,3-bis(3,5-dimethylphenyl)-4,6-diphenylpentalenide (Li_2[**6**])

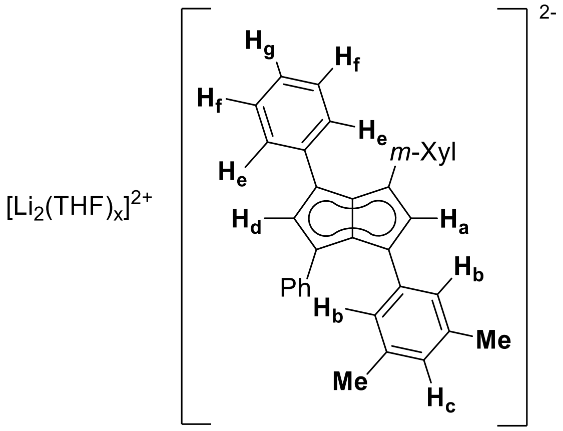

Reaction parameters: 5 eq. $LiNEt_2$ (0.60 mmol in 0.55 mL) and 1 eq. **^{m}Xyl$_2$Ph$_2$PnH$_2$** isomer mixture (0.12 mmol in 1 mL) used. Reaction volume in total: 1.55 mL. Quantitative conversion was achieved after one hour. The initial solubility was approximately $1.20 \cdot 10^{-4}$ mol **[^{m}Xyl$_2$Ph$_2$Pn]Li$_2$** in 1.55 mL THF at 20 °C. Solubility did not decrease after 24 hours.

^{1}H NMR (500 MHz, THF-h$_8$): δ = 7.15 (d, $^{3}J_{HH}$ = 7.2 Hz, 4H, H_e), 7.01 (*pseudo*-t, $^{3}J_{HH}$ = 7.2 Hz, $^{3}J_{HH}$ = 7.2 Hz, 4H, H_f), 6.79-6.78 (overlapping s's, 5H, H_b and H_d), 6.73 (s, 1H, H_a), 6.68 (t, $^{3}J_{HH}$ = 7.2 Hz, 2H, H_g), 6.34 (s, 2H, H_c), 2.11 (s, 12H, 4 CH_3).

^{13}C{^{1}H} NMR (126 MHz, THF-h$_8$): δ = 142.4, 141.7, 135.4, 127.6, 126.8, 126.7 (via HMBC), 125.5, 121.3, 119.3, 113.1, 112.2, 107.4, 106.9, 21.6.

^{7}Li NMR (194 MHz, THF-h$_8$): δ = −2.91, −4.78.

^{1}H DOSY (500 MHz, THF-h$_8$): D = $5.38 \cdot 10^{-10}$ m^{2}s^{-1}.

HR APCI-MS (−): m/z expected for [M−2Li]$^{\bullet -}$ = 462.2353; found = 462.2330.

3.5 Dilithium 1,3-bis(4-methoxyphenyl)-4,6-diphenylpentalenide (Li_2[**7**])

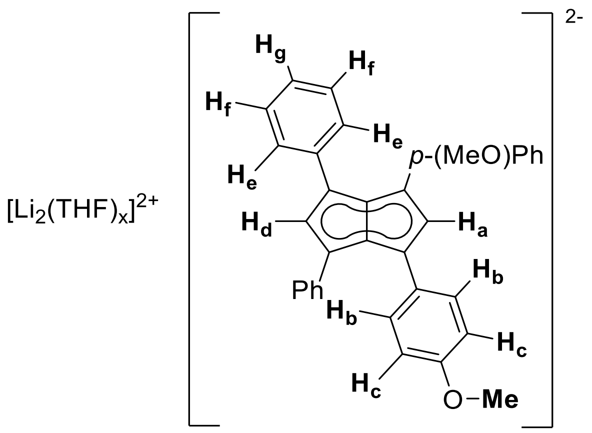

Reaction parameters: 3 eq. $LiNEt_2$ ($9.10\cdot10^{-5}$ mol in 0.3 mL) and 1 eq. **^{p}MeOPh$_2$Ph$_2$PnH$_2$** ($2.94\cdot10^{-5}$ mol in 0.3 mL) used. Reaction volume in total: 0.6 mL. Quantitative conversion was achieved after one week. The initial solubility was approximately $2.94\cdot10^{-5}$ mol **[^{p}MeOPh$_2$Ph$_2$Pn]Li$_2$** in 0.6 mL THF at 20 °C. Solubility did not decrease after several weeks.

^{1}H NMR (500 MHz, THF-h_8): δ = 7.13 (d, $^{3}J_{HH}$ = 7.31 Hz, 4H, H_e), 7.08 (d, $^{3}J_{HH}$ = 8.08 Hz, 4H, H_b), 6.97 (*pseudo*-t, $^{3}J_{HH}$ = 7.3 Hz, $^{3}J_{HH}$ = 7.3 Hz, 4H, H_f), 6.74 (s, 1H, H_d), 6.66–6.60 (m, 6H, H_g and H_c), 6.56 (s, 1H, H_a), 3.71 (s, 6H, CH_3).

^{13}C{^{1}H} NMR (126 MHz, THF-h_8): δ = 154.7, 142.4, 135.9, 128.1, 127.2, 127.1, 127.0, 119.1, 118.6 (via HMBC), 113.6, 112.9, 106.8, 105.5, 55.1.

^{7}Li NMR (194 MHz, THF-h_8): δ = −2.98, −5.37.

HR APCI-MS (−): m/z expected for $[M{-}2Li]^{\bullet-}$ = 466.1938 ; found = 466.1949.

3.6 Lithium potassium 1,3-bis(4-fluorophenyl)-4,6-di-p-tolylpentalenide (LiK[8])

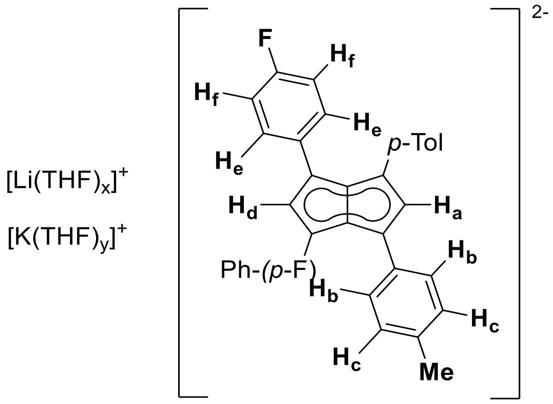

Reaction parameters: 3 eq. LiHMDS ($1.95{\cdot}10^{-4}$ mol in 0.4 mL) and 1 eq. **(^{p}FPh)$_2$(^{p}Tol)$_2$PnH$_2$** isomer mixture ($6.50{\cdot}10^{-5}$ mol in 0.5 mL) used and stirred for one hour, after which the mixture was added to a suspension of 3 eq. KH in THF ($1.95{\cdot}10^{-4}$ mol in 0.1 mL), followed by stirring for another 6.5 hours. Reaction volume in total: 1 mL. Quantitative conversion was achieved after 7.5 hours. The initial solubility was approximately $6.50{\cdot}10^{-5}$ mol **LiK[(^{p}FPh)$_2$(^{p}Tol)$_2$Pn]** in 1 mL THF at 20 °C. Solubility did not decrease for several weeks.

^{1}H NMR (500 MHz, THF-h$_8$): δ = 6.95–6.88 (m, 8H, H_b, H_e), 6.76 (d, $^3J_{HH}$ = 7.8 Hz, 4H, H_c), 6.69–6.61 (m, 6H, H_a, H_d, H_f), 2.19 (s, 6H, 2 CH_3).

^{13}C{^{1}H} NMR (126 MHz, THF-h$_8$): δ = 157.4 (d, $^1J_{CF}$ = 234.0 Hz, *C*F), 140.4, 139.6, 127.8, 126.2 (d, $^3J_{CF}$ = 6.0 Hz, *C*H=CH-CF), 126.0, 125.7, 123.1, 114.7, 114.6, 113.3 (d, $^2J_{CF}$ = 20.0 Hz, CH=*C*H-CF), 109.8, 106.3, 21.4.

^{19}F{^{1}H} NMR (471 MHz, THF-h$_8$): δ = −130.0.

HR APCI-MS (−): m/z expected for $[M{-}KLi]^{\bullet-}$ = 470.1852; found = 470.1868.

3.7 Dilithium 1,3-diphenylpentalenide (Li_2[9])

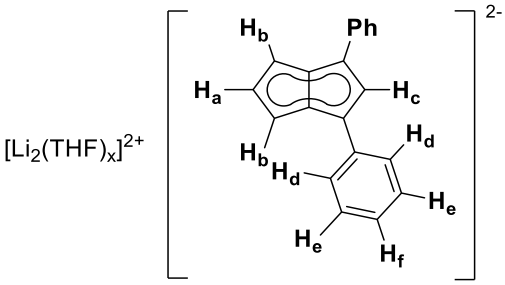

Reaction parameters: 4 eq. $LiNEt_2$ ($2.64 \cdot 10^{-4}$ mol in 0.4 mL) and 1 eq. **Ph_2PnH_2** isomer mixture ($6.60 \cdot 10^{-5}$ mol in 0.4 mL) used and stirred for 1.5 hours. Reaction volume in total: 0.8 mL. Quantitative conversion was achieved after 1.5 hours. The initial solubility was approximately $6.60 \cdot 10^{-5}$ mol **$Li_2[Ph_2Pn]$** in 0.8 mL THF at 20 °C. Solubility did not decrease for several weeks.

^{1}H NMR (500 MHz, THF-h_8): δ = 7.53 (d, $^3J_{HH}$ = 7.7 Hz, 4H, H_d), 7.05 (s, 1H, H_c), 7.01 (*pseudo*-t, $^3J_{HH}$ = 7.7 Hz, $^3J_{HH}$ = 7.7 Hz, 4H, H_e), 6.50–6.43 (m, 2H, H_f), 6.08–6.04 (m, 1H, H_a), 5.89–5.85 (m, 2H, H_b).

^{13}C{^{1}H} NMR (126 MHz, THF-h_8): δ = 143.0, 128.1, 124.3, 121.9, 117.6, 107.8 (via HSQC), 105.4, 103.4 (via HMBC), 84.5.

^{7}Li NMR (194 MHz, THF-h_8): δ = −3.47.

^{1}H DOSY (500 MHz, THF-h_8): D = $7.10 \cdot 10^{-10}$ m^2s^{-1}.

HR APCI-MS (−): m/z expected for $[M{-}2Li]^{\bullet-}$ = 254.1101; found = 254.1027.

3.8. Dilithium 1,3-di(4-fluorophenyl)pentalenide (Li_2[**10**])

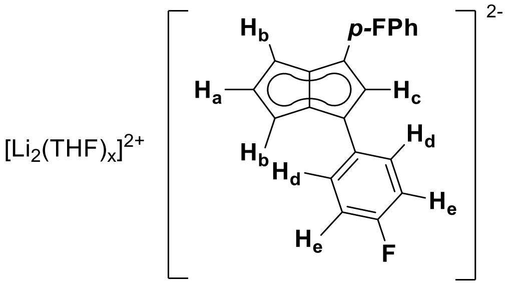

Reaction parameters: 3 eq. $LiNEt_2$ ($2.55\cdot10^{-4}$ mol in 0.3 mL) and 1 eq. **$^{p}FPh_2PnH_2$** isomer mixture ($8.50\cdot10^{-5}$ mol in 0.3 mL) used and stirred for 1 hour. Reaction volume in total: 0.6 mL. Nearly quantitative conversion (>90%) was achieved after 1 hour. The initial solubility was approximately $8.50\cdot10^{-5}$ mol **$Li_2[(^{p}FPh)_2Pn]$** in 0.6 mL THF at 20 °C. The solubility did not decrease for several weeks.

^{1}H NMR (500 MHz, THF-h_8): δ = 7.49–7.42 (m, 4H, H_d), 6.92 (s, 1H, H_c), 6.76 (*pseudo*-t, $^{3}J_{HH}$ = 8.6 Hz, $^{3}J_{HH}$ = 8.6 Hz, 4H, H_e), 6.06 (s, 1H, H_a), 5.82 (s, 2H, H_b).

$^{13}C\{^{1}H\}$ NMR (126 MHz, $CDCl_3$): δ = 157.2 (d, $^{1}J_{CF}$ = 232.0 Hz, *C*F), 139.8, 124.1, 122.1, 114.7 (d, $^{2}J_{CF}$ = 21.0 Hz, CH=*C*H-CF), 107.7 (via HSQC), 105.6, 102.0 (via HMBC), 84.2.

$^{19}F\{^{1}H\}$ NMR (471 MHz, THF-h_8): δ = −129.8.

^{7}Li NMR (194 MHz, THF-h_8): δ = −0.94, −4.03.

^{1}H DOSY (500 MHz, THF-h_8): D = $6.88\cdot10^{-10}$ m^2s^{-1}.

HR APCI-MS (−): m/z expected for $[M-2Li]^{\bullet-}$ = 290.0913; found = 290.0890.

3.9. Deprotonation product of 1,3,8-triphenyl-4,5,6,7,7a,8-hexahydrocyclopenta-[a]-indene and $LiNEt_2$ (Li[**11-exo**])

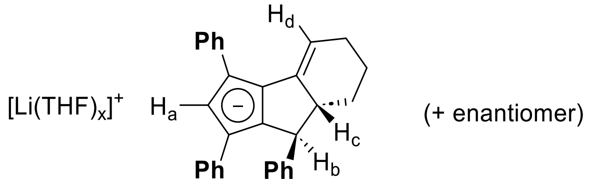

Under argon and stirring 1,3,8-triphenyl-4,5,6,7,7a,8-hexahydrocyclopenta-[a]-indene (9 mg, $2.33 \cdot 10^{-5}$ mol) in 0.4 mL THF was added dropwise (3 minutes) to $LiNEt_2$ (8 mg, $9.32 \cdot 10^{-5}$ mol) in 0.2 mL THF. The dark red solution was stirred for further 90 mins, after which it was transferred into a J. Young NMR tube. The ^{1}H NMR spectrum showed quantitative conversion of the starting material into **13-exo**.

^{1}H NMR (500 MHz, THF-h_8): δ = 7.62 (d, $^3J_{HH}$ = 7.5 Hz, 2H, Ph*H*), 7.32 (d, $^3J_{HH}$ = 7.5 Hz, 2H, Ph*H*), 7.09 (*pseudo*-t, $^3J_{HH}$ = 7.5 Hz, $^3J_{HH}$ = 7.5 Hz, 2H, Ph*H*), 7.04–6.96 (m, 5H, Ph*H*), 6.65 (*pseudo*-t and t, $^3J_{HH}$ = 7.5 Hz, $^3J_{HH}$ = 7.5 Hz, $^3J_{HH}$ = 7.5 Hz, 3H, Ph*H*), 6.53 (s, 1H, H_a), 6.37 (t, $^3J_{HH}$ = 7.5 Hz, 1H, Ph*H*), 5.36–5.33 (m, 1H, H_d), 3.87 (d, $^3J_{HH}$ = 6.5 Hz, integral influenced by solvent, H_b), 2.74 (H_c via ^{1}H-^{1}H COSY and HSQC, signal covered by LiN*Et*$_2$/HN*Et*$_2$ system), 2.20/2.07/1.52/1.50 (all C*H*$_2$, via ^{1}H-^{1}H COSY, HSQC and HMBC; integral influenced by solvent).

^{13}C{^{1}H} NMR (126 MHz, THF-h_8): δ = 150.7, 143.8, 142.3, 132.5, 130.3, 128.5, 128.1, 127.8, 127.6, 127.4, 125.7, 125.2, 124.6, 120.3, 119.3, 115.7, 115.3, 110.7, 101.6, 59.7, 55.0, 29.4, 26.6, 24.7.

^{7}Li NMR (194 MHz, THF-h_8): δ = 2.27, 0.50.

HR APCI-MS (−): m/z expected for $[M-Li]^-$ = 385.1956; found = 385.1859.

3.10. Lithium 3-vinyl-1,4,6-triphenyl-1,2,2-trihydropentalenide (Li[**12-exo**])

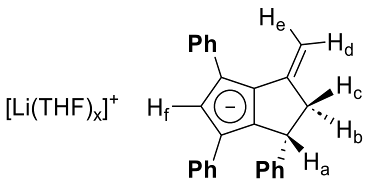

Under argon **3-Me-1,4,6-Ph₃PnH₂** (10.0 mg, 0.029 mmol) in 0.2 mL THF-d_8 was added dropwise to LiHMDS (5.6 mg, 0.032 mmol) in 0.2 mL THF-d_8. The bright yellow solution was allowed to stand for 30 mins, and then was filtered into a J. Young NMR tube. The ^{1}H NMR spectrum showed quantitative conversion of the starting material into **12-exo Li[3-(CH_2)-Ph_3PnH_2]**.

^{1}H NMR (500 MHz, THF-d_8): δ = 7.62 (d, $^3J_{HH}$ = 8.1 Hz, 2H, *o*-Ph), 7.20 (d, $^3J_{HH}$ = 7.5 Hz, 2H, *o*-Ph), 7.17 (d, $^3J_{HH}$ = 7.4 Hz, 2H, *o*-Ph), 7.06–6.99 (m, 4H, *m*-Ph), 6.90 (t, $^3J_{HH}$ = 7.5 Hz, 1H, *p*-Ph), 6.78 (*pseudo*-t, $^3J_{HH}$ = 7.5 Hz, $^3J_{HH}$ = 7.5 Hz, 2H, *m*-Ph), 6.69 (t, $^3J_{HH}$ = 7.4 Hz, 1H, *p*-Ph), 6.52 (s, 1H, H_f), 6.42 (t, $^3J_{HH}$ = 7.4 Hz, 1H, *p*-Ph), 4.76–4.74 (m, 1H, H_e), 4.38 (dd, $^3J_{HH}$ = 8.2 Hz, $^3J_{HH}$ = 1.6 Hz, 1H, H_a), 4.02–4.00 (m, 1H, H_d), 3.57–3.50 (m, 1H, H_c), 2.64 (d, $^2J_{HH}$ = 14.6 Hz, 1H, H_b).

^{13}C{^{1}H} NMR (126 MHz, THF-d_8): δ = 151.4, 149.5, 144.3, 142.8, 137.9, 128.2, 127.94, 127.88, 127.7, 126.8, 126.3, 124.8, 123.5, 120.4, 119.0, 117.0, 115.3, 112.8, 87.6, 52.5, 46.7.

HR APCI-MS (−): m/z expected for $[M−Li]^−$ = 345.1643; found = 345.1622.

3.11. Dilithium 1-methyl-3,4,6-triphenylpentalenide (Li_2[**12**])

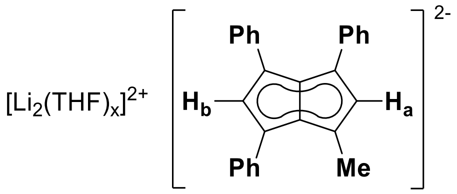

Reaction parameters: 4 eq. $LiNEt_2$ ($6.80\cdot10^{-5}$ mmol in 0.25 mL) and 1 eq. **1-Me-3,4,6-Ph_3PnH_2** ($1.70\cdot10^{-5}$ mol in 0.35 mL) used. Reaction volume in total: 0.6 mL. Quantitative conversion was achieved after 1.5 hours. The initial solubility was approximately $1.70\cdot10^{-5}$ mol **Li_2[$MePh_3Pn$]**/0.6 mL THF at 20 °C. The solubility did not decrease for several weeks.

^{1}H NMR (500 MHz, THF-h_8): δ = 7.64 (d, $^3J_{HH}$ = 7.7 Hz, 2H, o-Ph*H*), 7.06–7.01 (m, 4H, *o*-Ph*H*, *o*-Ph*H*), 6.98–6.88 (m, 6H, *m*-Ph*H*, *m*-Ph*H*, *m*-Ph*H*), 6.66 (s, 1H, H_b), 6.61–6.56 (m, 2H, *p*-Ph*H*, *p*-Ph*H*), 6.54–6.49 (m, 1H, *p*-Ph*H*), 6.19 (s, 1H, H_a), 2.69 (s, 3H, CH_3).

^{13}C{^{1}H} NMR (126 MHz, THF-h_8): δ = 143.4 (via HMBC), 142.1, 128.1, 127.5 (via HMBC), 127.1, 126.9, 125.2, 122.8 (via HMBC), 118.9, 118.7, 117.9 (via HSQC), 112.8, 106.5, 99.2, 18.4.

^{7}Li NMR (194 MHz, THF-h_8): δ = −0.09, −4.91.

^{1}H DOSY (500 MHz, THF-h_8): D = $6.93\cdot10^{-10}$ m^2s^{-1}.

HR APCI-MS (−): m/z expected for $[M-2Li]^{\bullet-}$ = 344.1570 ; found = 344.1580.

3.12. Overview wingtip ^{1}H and *ipso* ^{13}C chemical shifts

Table S1: ^{13}C NMR chemical shifts of C1, C3, C4 and C6 in variously substituted dilithium pentalenides in THF at room temperature.

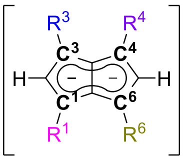

**Pn^{2-}**	**R^1**	**R^3**	**R^4**	**R^6**	**δ(C^1) [ppm]**	**δ(C^3) [ppm]**	**δ(C^4) [ppm]**	**δ(C^6) [ppm]**
**2**	Ph	Ph	Ph	Ph	109.5 §	109.5 §	109.5 §	109.5 §
**3**	^{p}Tol	^{p}Tol	^{p}Tol	^{p}Tol	106.4	106.4	106.4	106.4
**4**	^{m}Xyl	^{m}Xyl	^{m}Xyl	^{m}Xyl	107.1	107.1	107.1	107.1
**5**	Ph	Ph	^{p}Tol	^{p}Tol	107.2	107.2	106.6	106.6
**6**	^{m}Xyl	^{m}Xyl	Ph	Ph	106.9	106.9	107.4	107.4
**7**	^{p}MeO-Ph	^{p}MeO-Ph	Ph	Ph	105.5	105.5	106.8	106.8
**8**	^{p}F-Ph	^{p}F-Ph	^{p}Tol	^{p}Tol	109.3	109.3	109.8	109.8
**9**	Ph	Ph	H	H	103.5	103.5	84.3	84.3
**10**	^{p}F-Ph	^{p}F-Ph	H	H	102.1	102.1	83.9	83.9
**12**	Me	Ph	Ph	Ph	99.2	106.6	106.6	106.6

§ Values reported for ***LiK[Ph$_4$Pn]*** *in THF.*[3]

3.13. *anti*-bis(2,5-Norbornadiene)dirhodium 1,3-bis(4-methoxyphenyl)-4,6-diphenyl-pentalenide (Rh_2NBD_2[**7**])

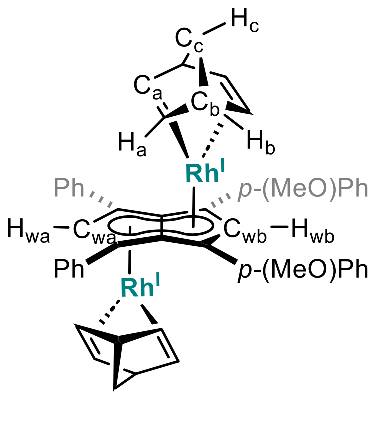

Na_2[1,3-Ph_2-4,6-(^{p}MeO)Ph_2Pn] was generated *in-situ* by the addition of $NaNH_2$ (15 mg, 0.38 mmol in 0.2 mL THF) to **^{p}MeO$Ph_2Ph_2PnH_2$** (20 mg, 0.04 mmol in 0.3 mL THF) followed by stirring for 18 hours at room temperature. Formation was confirmed by ^{1}H NMR, after which [Rh(NBD)(μ-Cl)]$_2$ (20 mg, 0.04 mmol in 0.5 mL THF) was added and a colour change from dark red to dark yellow was observed, with full conversion confirmed by NMR. Crystals suitable for XRD could be grown by addition of hexane to a THF solution followed by standing at -35 °C for 48 hours.

^{1}H NMR (500 MHz, THF-h_8): δ = 7.20–7.16 (m, 4H, Ar*H*), 7.13–7.05 (m, 10H, Ar*H*), 6.66 (d, $^3J_{HH}$ = 8.4 Hz, 4H, Ar*H*), 6.19 (s, 1H, H_{wa}), 6.11 (s, 1H, H_{wb}), 3.72 (s, *via* HSQC and HMBC, integral overlap with solvent, ArO*Me*), 3.31 (bs, *via* HSQC and HMBC, integral influenced by solvent, H_b), 3.15 (bs, 8H, H_a), 0.90–0.86 (m, 4H, H_c).

^{1}H NMR (500 MHz, DCM-d_2): δ = 7.16–7.11 (m, 10H, Ar*H*), 7.08–7.04 (m, 4H, Ar*H*), 6.68–6.65 (m, 4H, Ar*H*), 6.17 (d, $^2J_{RhH}$ = 0.8 Hz, 1H, H_{wb}), 6.07 (d, $^2J_{RhH}$ = 0.9 Hz, 1H, H_{wa}), 3.76 (s, 6H, O*Me*), 3.35–3.31 (m, 4H, H_b), 3.18–3.15 (m, 8H, H_a), 0.91–0.88 (m, 4H, H_c).

^{13}C{^{1}H} NMR (126 MHz, DCM-d_2): δ = 158.4, 136.7, 130.4, 129.4, 128.9, 127.9, 126.1, 113.3, 96.7 (d, $^1J_{RhC}$ = 5.8 Hz, C_{wb}), 96.2 (d, $^1J_{RhC}$ = 6.2 Hz, C_{wa}), 88.6 (d, $^1J_{RhC}$ = 5.7 Hz), 88.2 (d, $^1J_{RhC}$ = 4.6 Hz), 58.3 (d, $^1J_{RhC}$ = 7.6 Hz, C_c), 55.6 (O*Me*), 48.5 (d, $^1J_{RhC}$ = 2.5 Hz, C_b), 41.3 (d, $^1J_{RhC}$ = 10.6 Hz, C_a), 41.2 (d, $^1J_{RhC}$ = 10.7 Hz, C_a).

HR APCI-MS (+): m/z expected for $[M+H]^+$ = 857.1368, found = 857.1199.

4. NMR spectra of novel starting materials (Figures S1–S10)

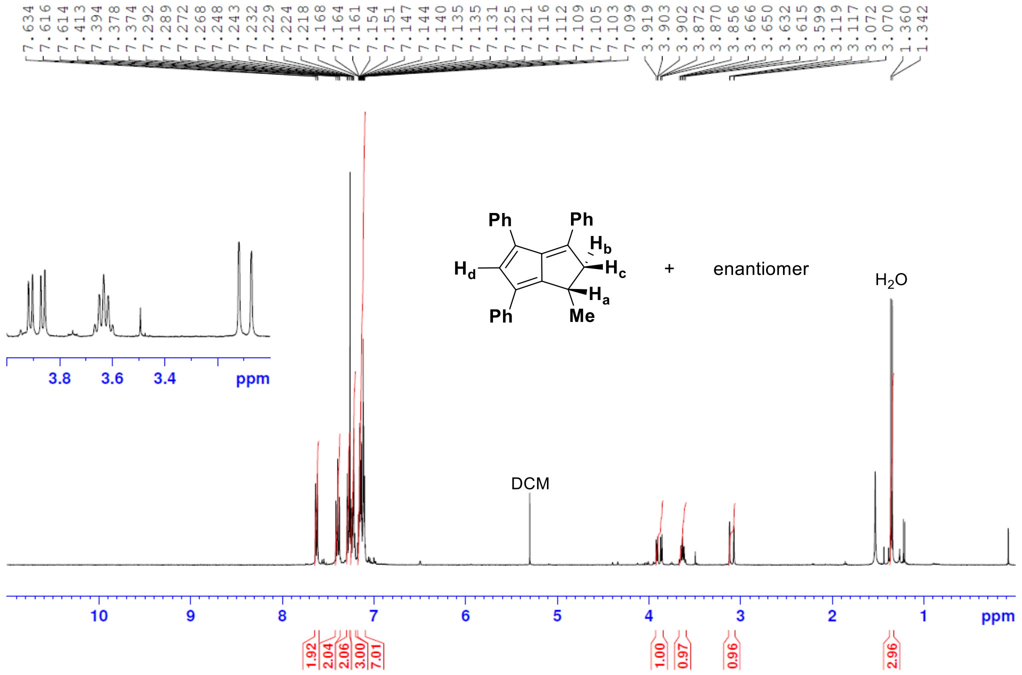

Figure S1: ^{1}H NMR 1-methyl-3,4,6-triphenyl-1,2-dihydropentalene **12’**H_2 (400 MHz, $CDCl_3$, 298 K).

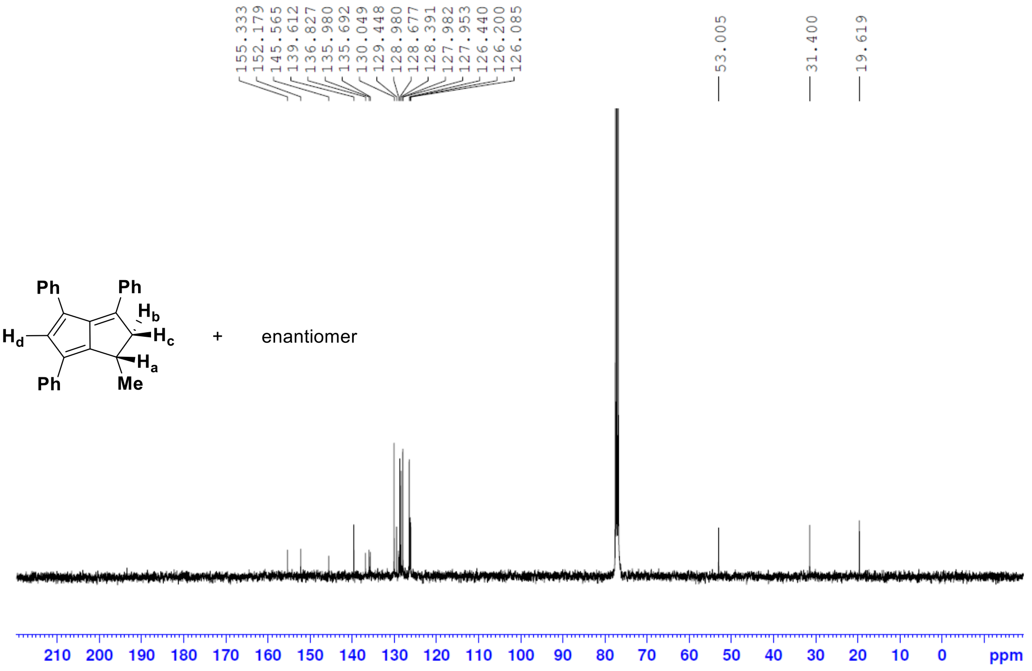

Figure S2: $^{13}C\{^{1}H\}$ NMR 1-methyl-3,4,6-triphenyl-1,2-dihydropentalene **12’**H_2

(101 MHz, $CDCl_3$, 298 K).

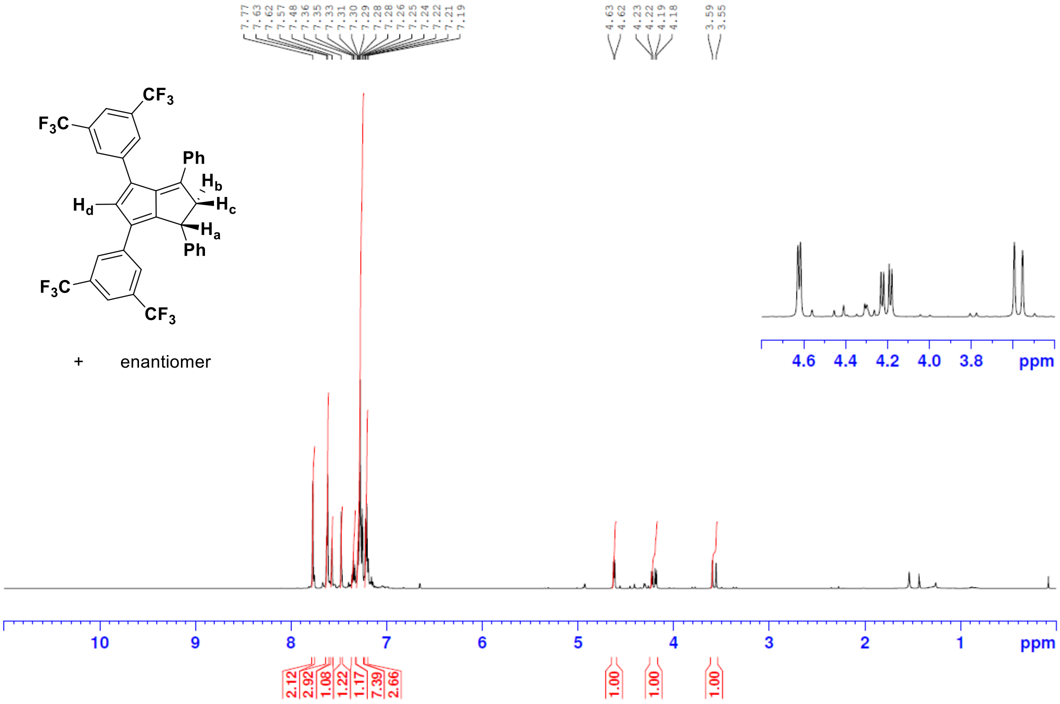

Figure S3: ^{1}H NMR 1,3-diphenyl-4,6-bis(3,5-bis(trifluoromethyl)phenyl)-1,2-dihydropentalene **8d**-H_2 (500 MHz, $CDCl_3$, 298 K).

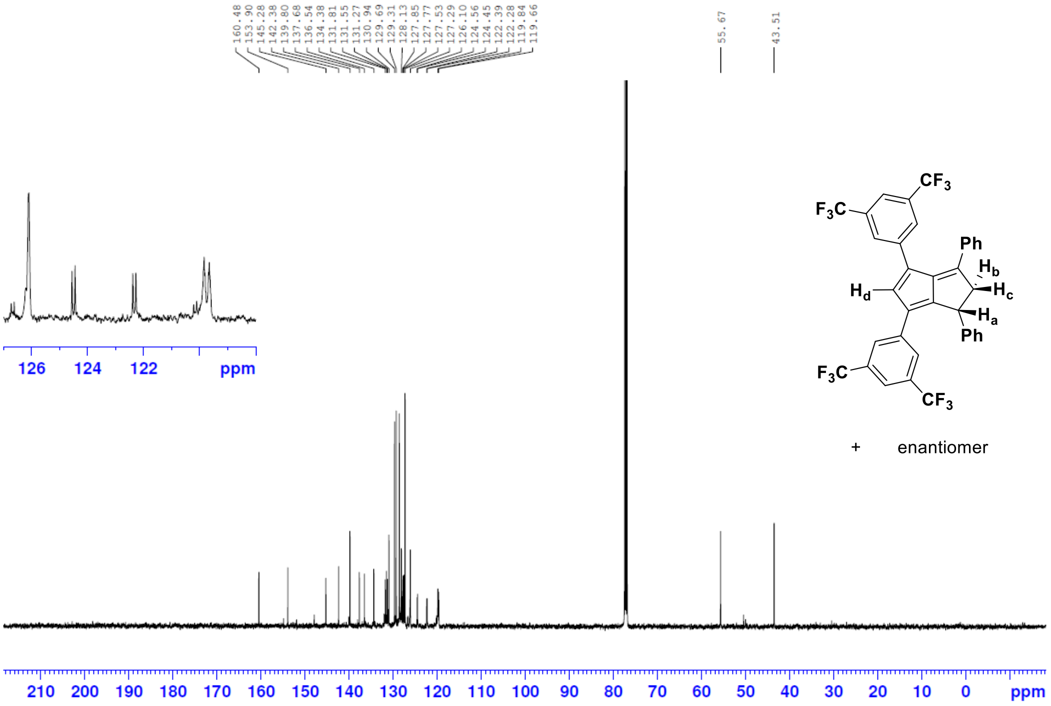

Figure S4: $^{13}C\{^{1}H\}$ NMR 1,3-diphenyl-4,6-bis(3,5-bis(trifluoromethyl)phenyl)-1,2-dihydropentalene **8d**-H_2 (126 MHz, $CDCl_3$, 298 K).

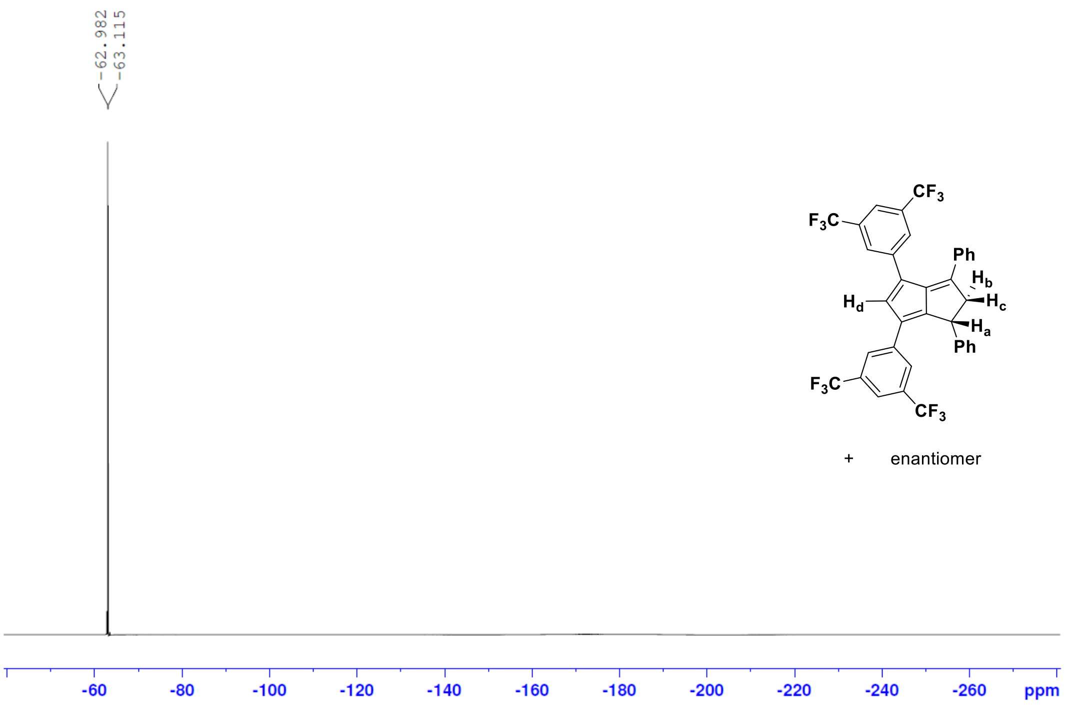

Figure S5: $^{19}F\{^{1}H\}$ NMR 1,3-diphenyl-4,6-bis(3,5-bis(trifluoromethyl)phenyl)-1,2-dihydropentalene **8d**-H_2 (471 MHz, $CDCl_3$, 298 K).

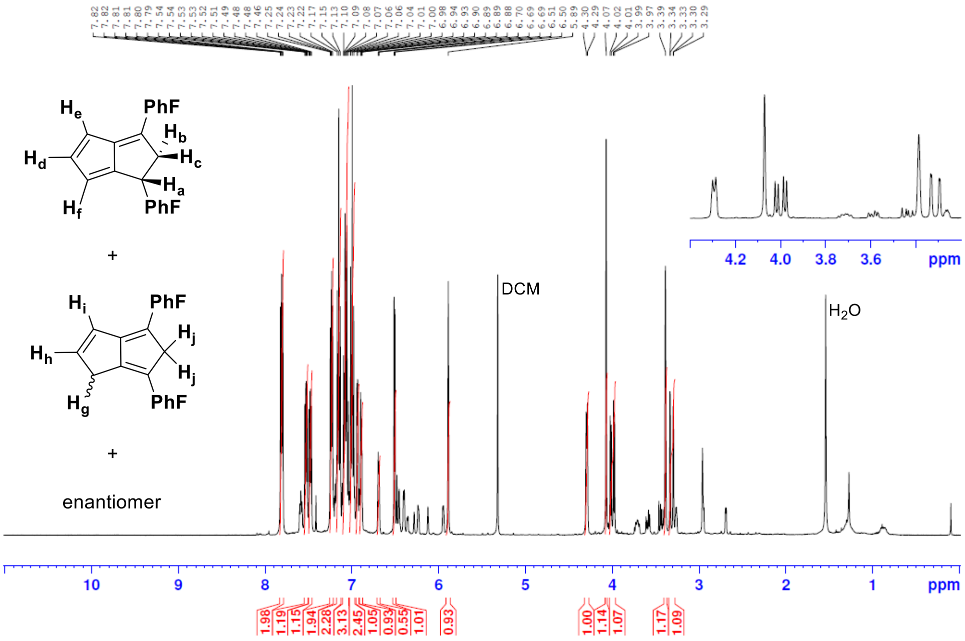

Figure S6: ^{1}H NMR 1,3-bis(4-fluorophenyl)-1,2-dihydropentalene and 4,6-bis(4-fluorophenyl)-1,5-dihydropentalene **10**H_2 (500 MHz, CD_2Cl_2, 298 K).

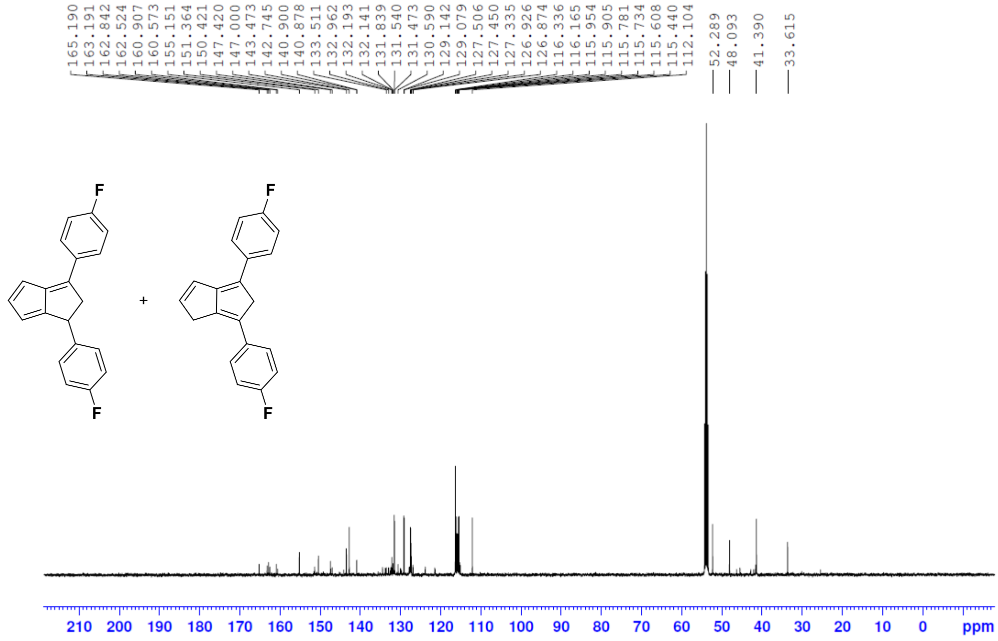

Figure S7: $^{13}C\{^{1}H\}$ NMR 1,3-bis(4-fluorophenyl)-1,2-dihydropentalene and 4,6-bis(4-fluorophenyl)-1,5-dihydropentalene **10**H_2 (126 MHz, CD_2Cl_2, 298 K).

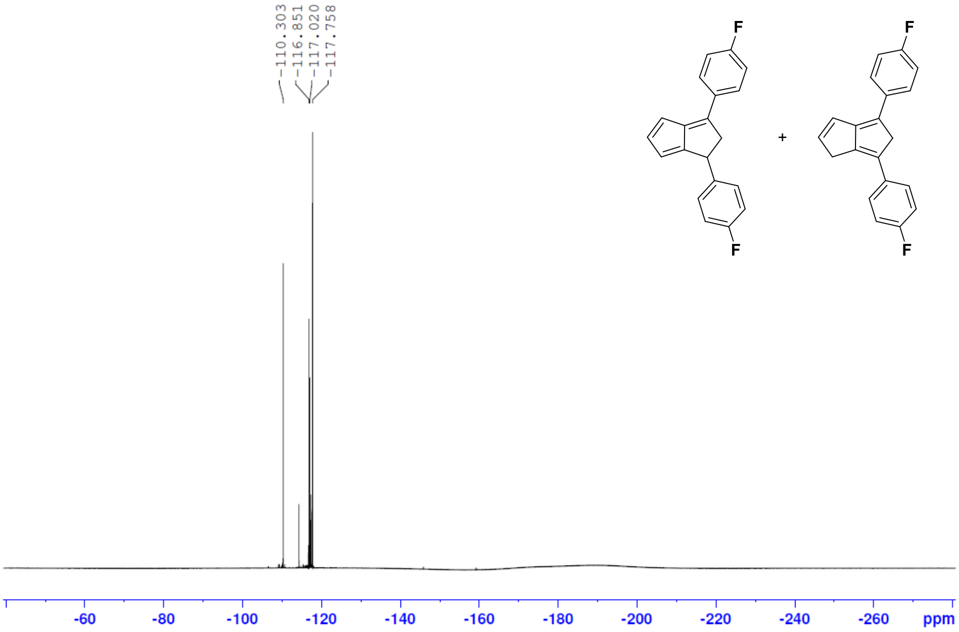

Figure S8: $^{19}F\{^{1}H\}$ NMR 1,3-bis(4-fluorophenyl)-1,2-dihydropentalene and 4,6-bis(4-fluorophenyl)-1,5-dihydropentalene **10**H_2 (471 MHz, CD_2Cl_2, 298 K).

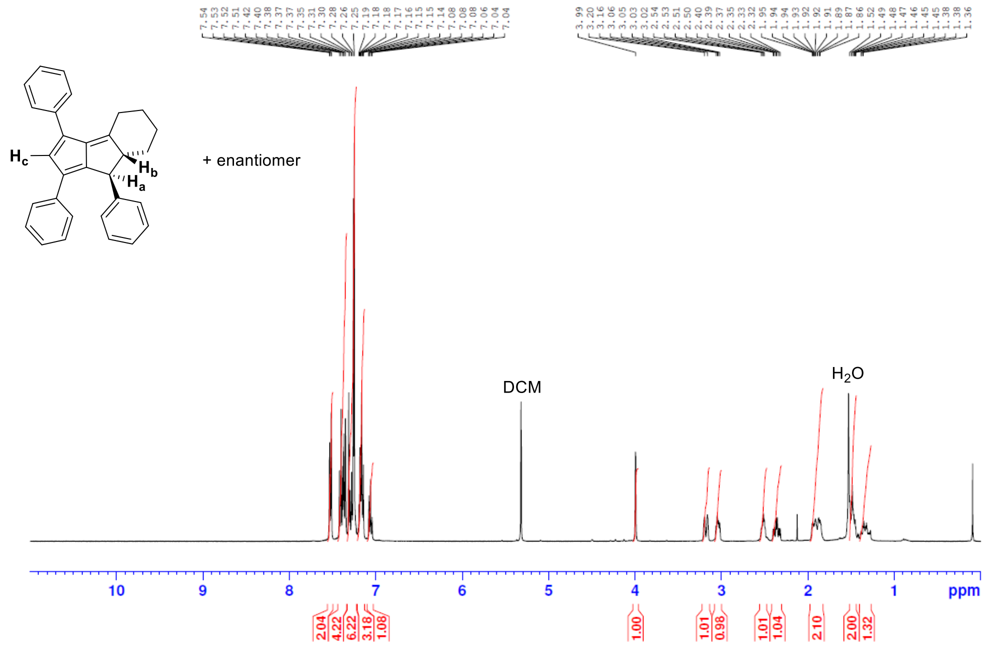

Figure S9: ^{1}H NMR 1,3,8-triphenyl-4,5,6,7,7a,8-hexahydro-cyclopenta-[a]-indene

11H_2 (400 MHz, CD_2Cl_2, 298 K).

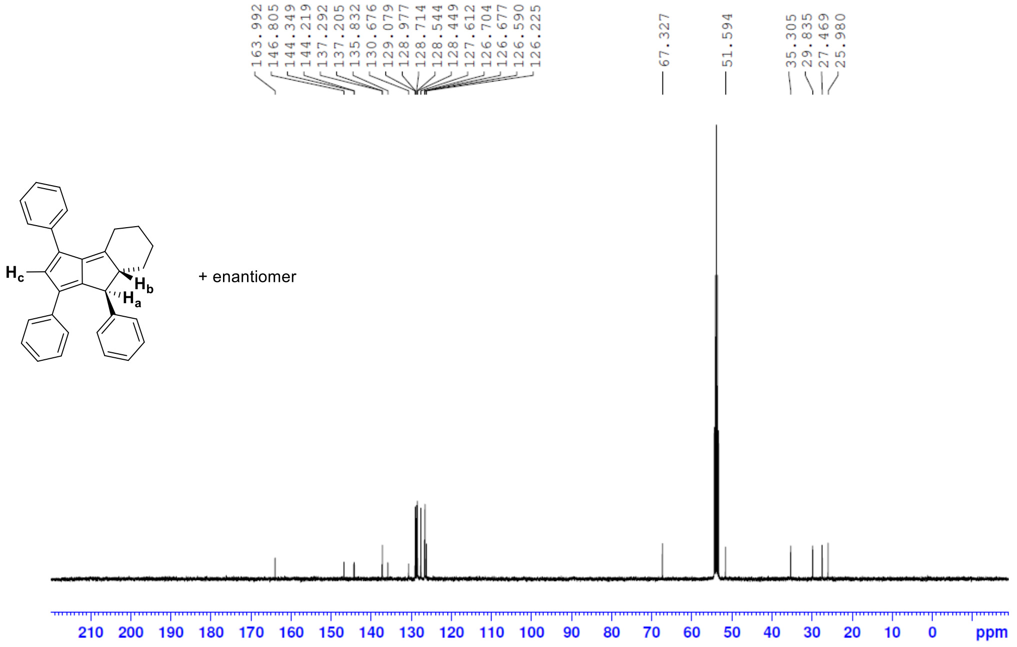

Figure S10: ^{13}C{^{1}H} NMR 1,3,8-triphenyl-4,5,6,7,7a,8-hexahydro-cyclopenta-[a]-indene

11H_2 (101 MHz, CD_2Cl_2, 298 K).

5. NMR spectra of products (Figures S11–S53)

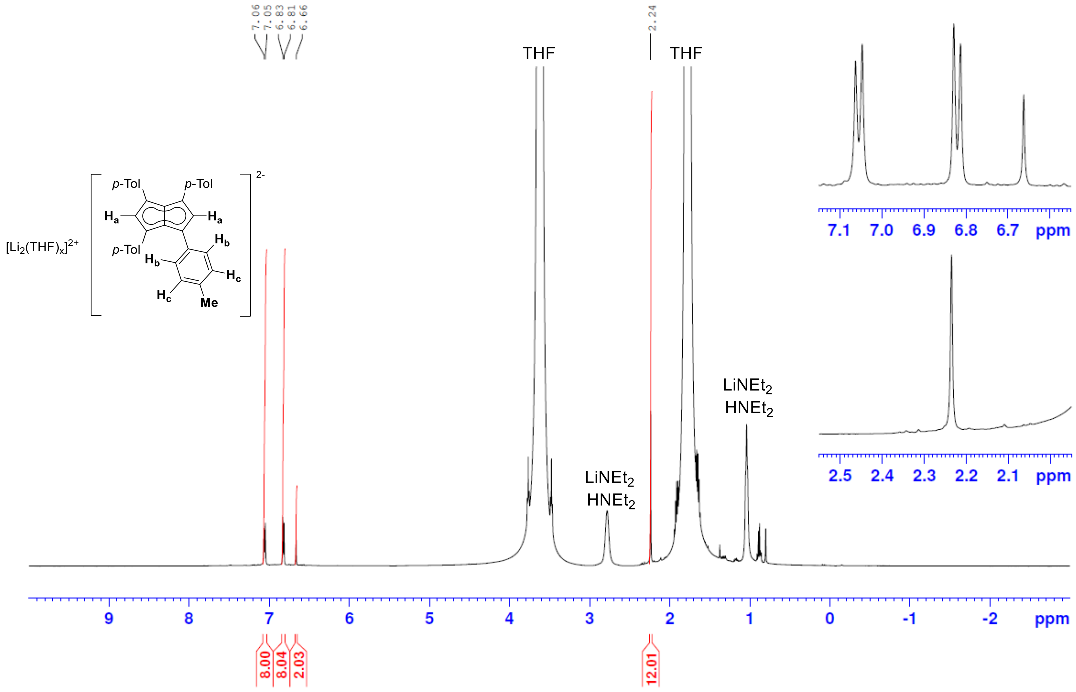

Figure S11: ^{1}H NMR dilithium 1,3,4,6-tetra-*p*-tolylpentalenide Li_2[**3**] (500 MHz, THF-h_8, 298 K).

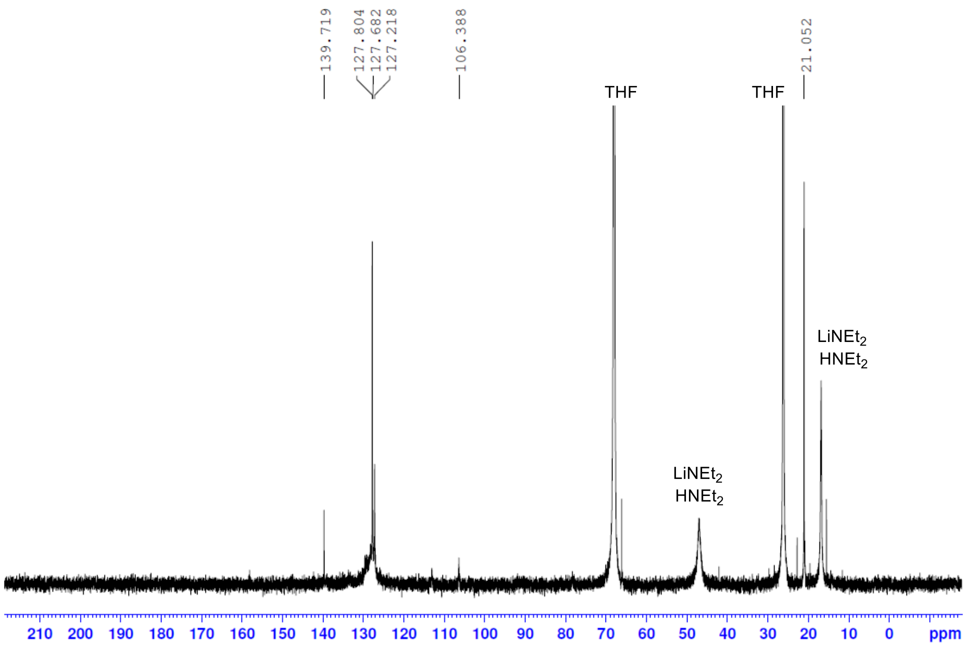

Figure S12: $^{13}C\{^{1}H\}$ NMR dilithium 1,3,4,6-tetra-*p*-tolylpentalenide Li_2[**3**] (126 MHz, THF-h_8, 298 K).

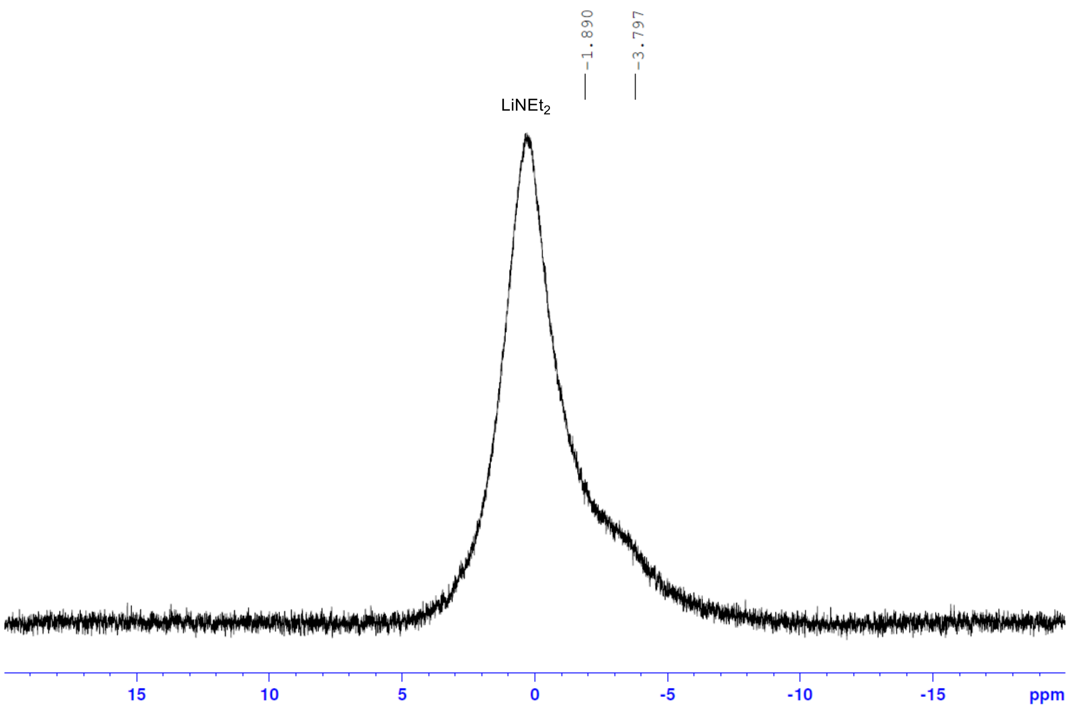

Figure S13: ^{7}Li NMR dilithium 1,3,4,6-tetra-*p*-tolylpentalenide Li_2[**3**] (194 MHz, THF-h_8, 298 K).

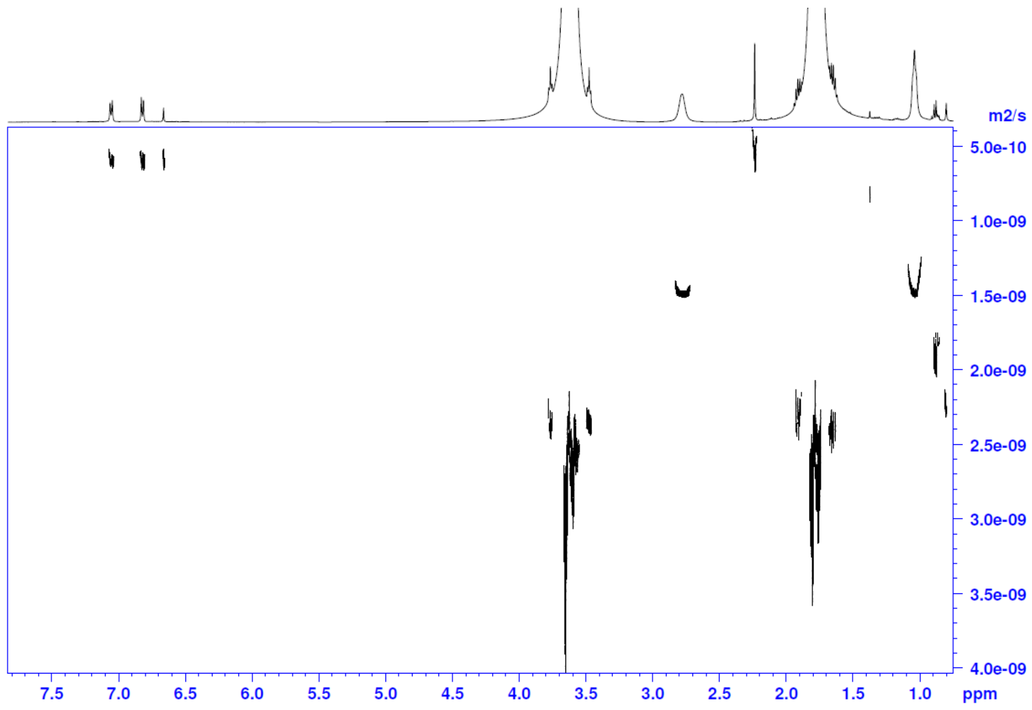

Figure S14: ^{1}H DOSY dilithium 1,3,4,6-tetra-*p*-tolylpentalenide Li_2[**3**] (500 MHz, THF-h_8, 298 K).

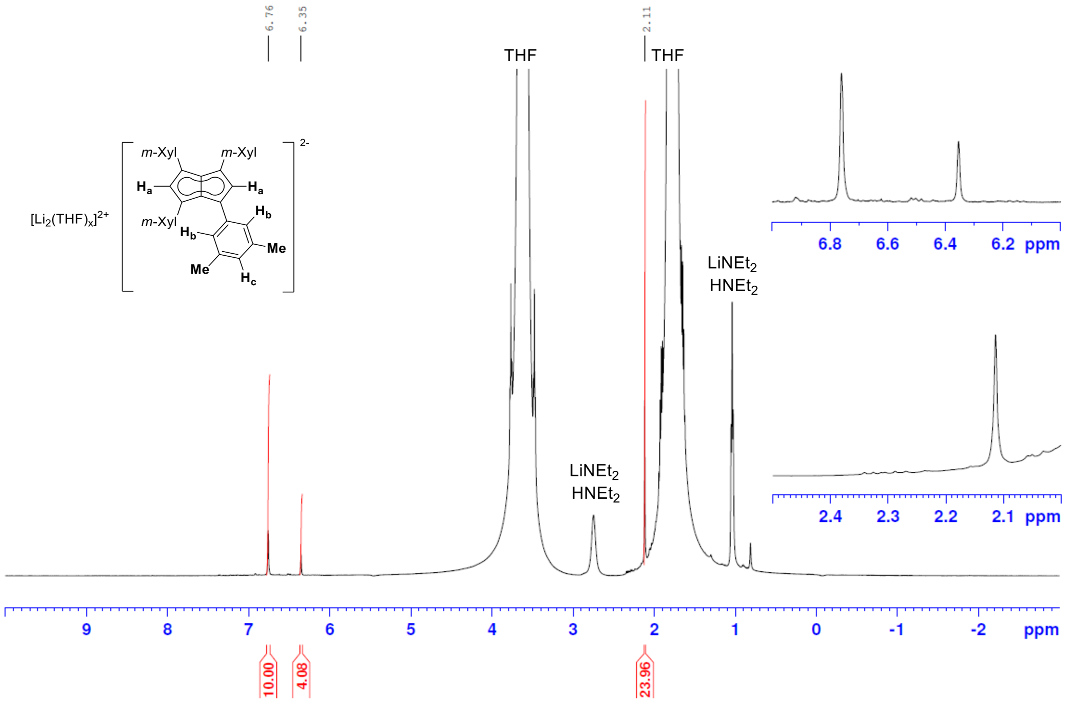

Figure S15: ^{1}H NMR dilithium 1,3,4,6-tetrakis(3,5-dimethylphenyl)pentalenide Li_2[**4**] (500 MHz, THF-h_8, 298 K).

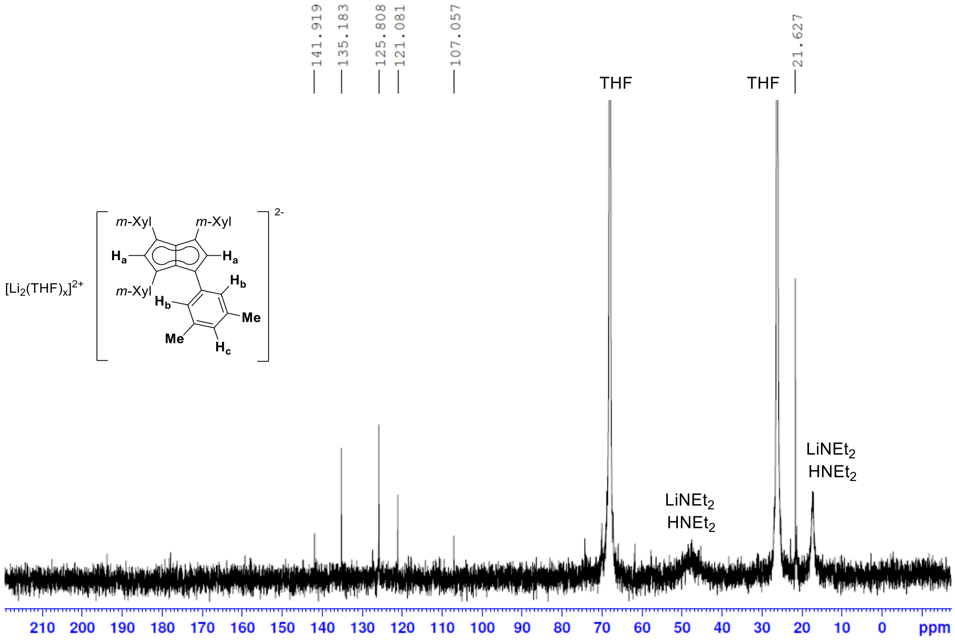

Figure S16: $^{13}C\{^{1}H\}$ NMR dilithium 1,3,4,6-tetrakis(3,5-dimethylphenyl)pentalenide Li_2[**4**] (126 MHz, THF-h_8, 298 K).

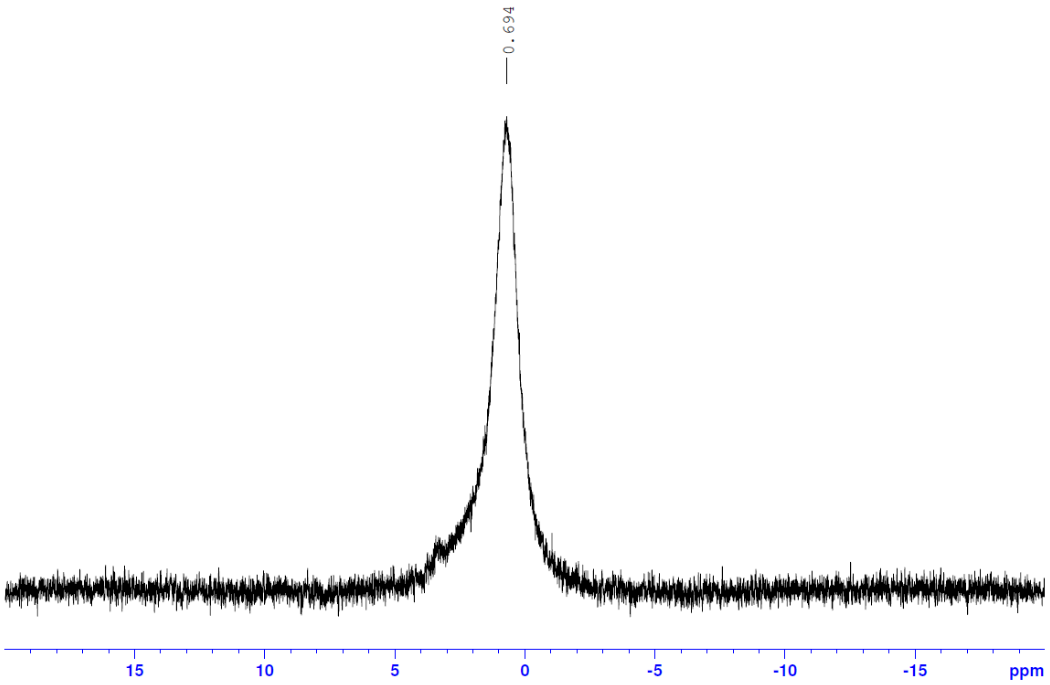

Figure S17: ^{7}Li NMR dilithium 1,3,4,6-tetrakis(3,5-dimethylphenyl)pentalenide Li_2[**4**] (194 MHz, THF-h_8, 298 K).

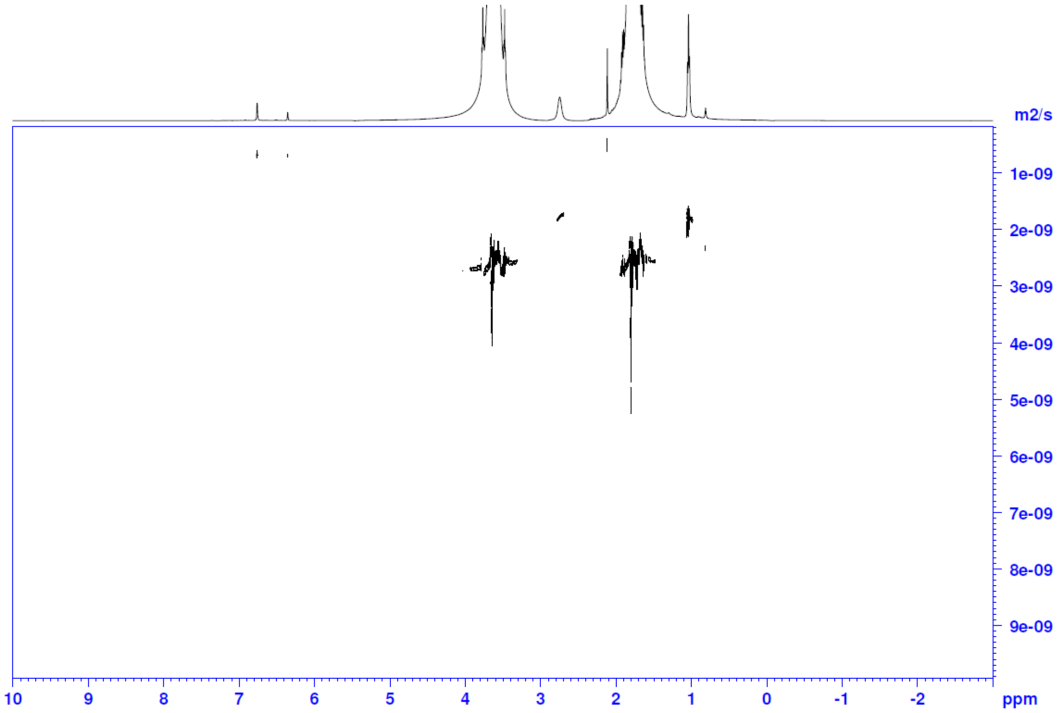

Figure S18: ^{1}H DOSY dilithium 1,3,4,6-tetrakis(3,5-dimethylphenyl)pentalenide Li_2[**4**] (500 MHz, THF-h_8, 298).

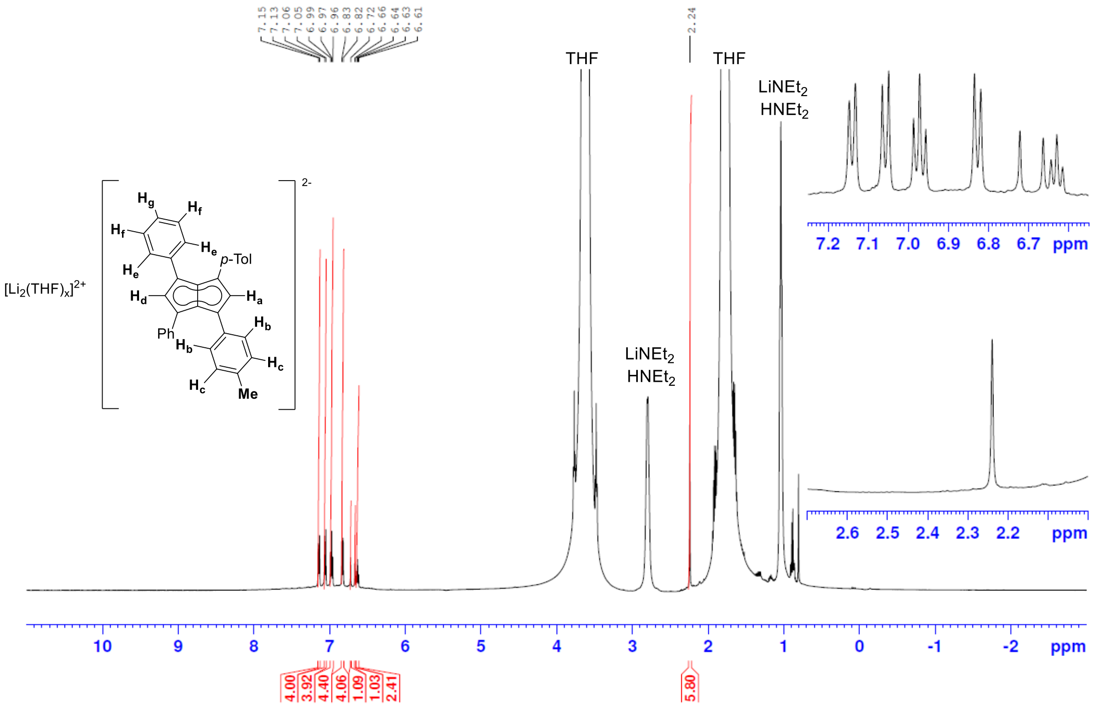

Figure S19: ^{1}H NMR dilithium 1,3-diphenyl-4,6-di-*p*-tolylpentalenide Li_2[**5**] (500 MHz, THF-h_8, 298 K).

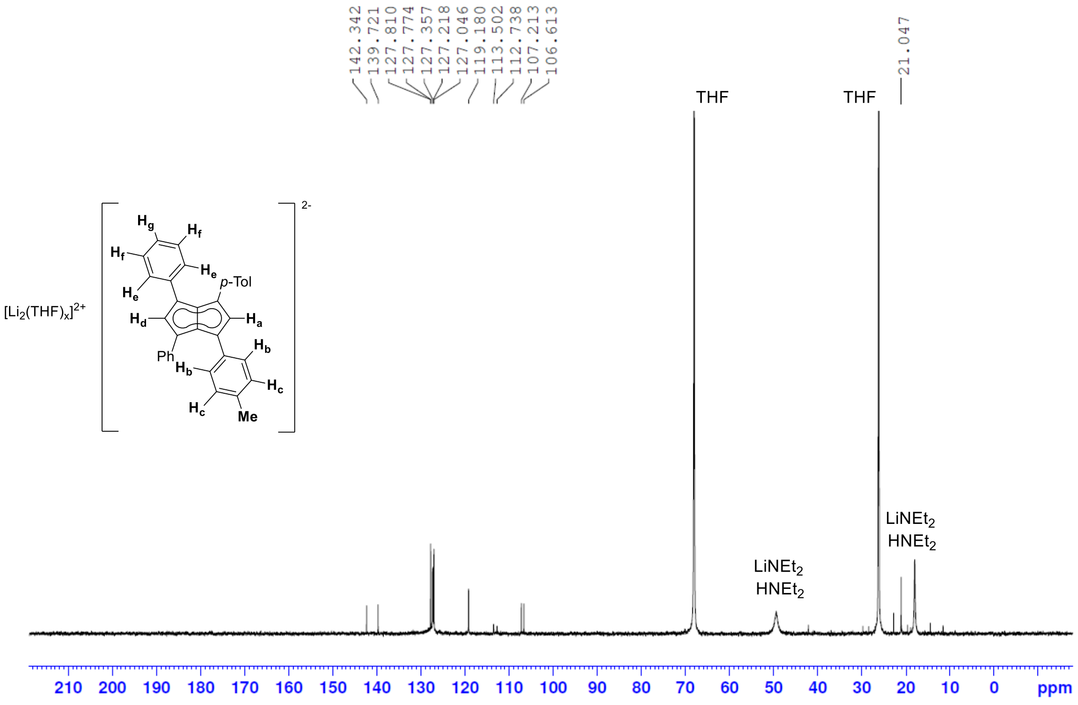

Figure S20: ^{13}C{^{1}H} NMR dilithium 1,3-diphenyl-4,6-di-*p*-tolylpentalenide Li_2[**5**] (126 MHz, THF-h_8, 298 K).

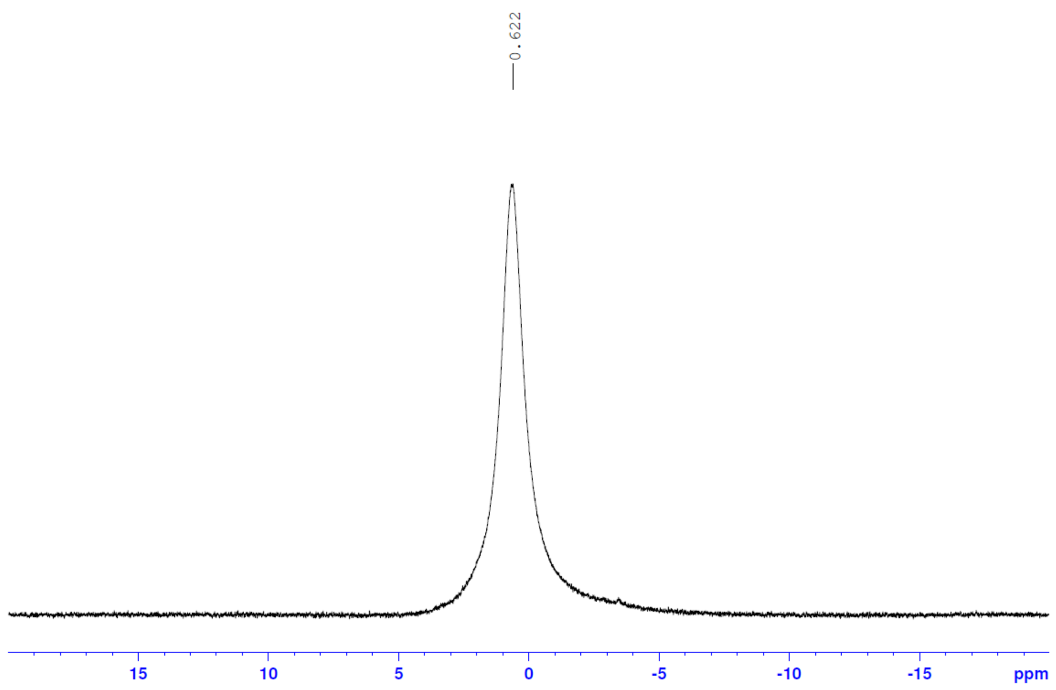

Figure S21: ^{7}Li NMR dilithium 1,3-diphenyl-4,6-di-*p*-tolylpentalenide Li_2[**5**] (194 MHz, THF-h_8, 298 K).

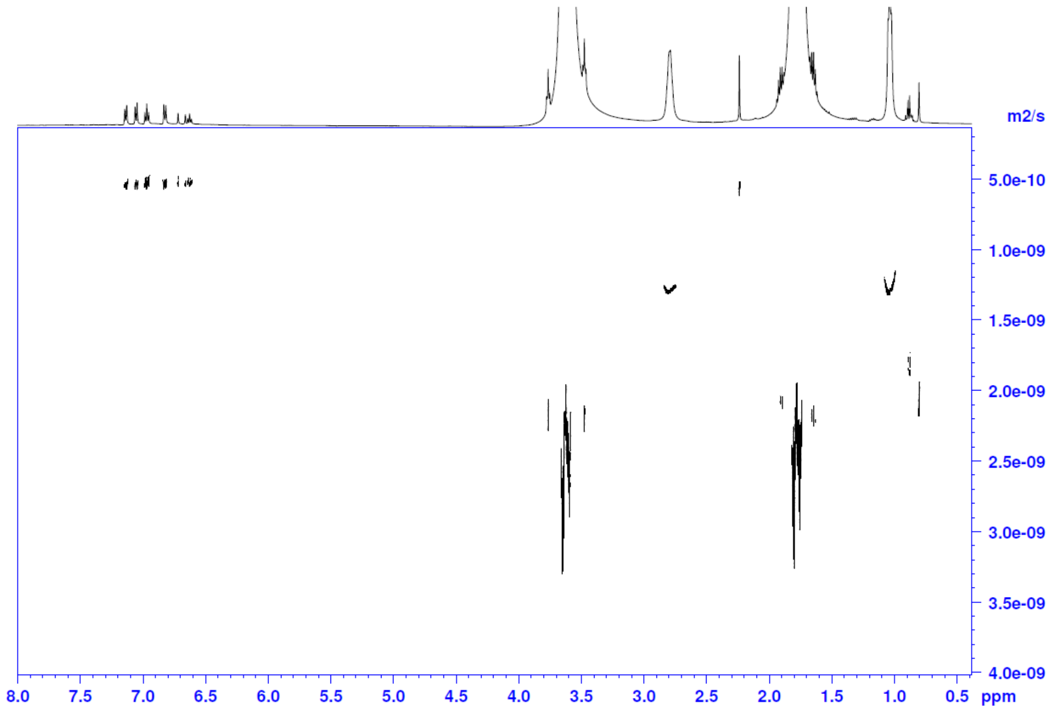

Figure S22: ^{1}H DOSY dilithium 1,3-diphenyl-4,6-di-*p*-tolylpentalenide Li_2[**5**] (500 MHz, THF-h_8, 298 K).

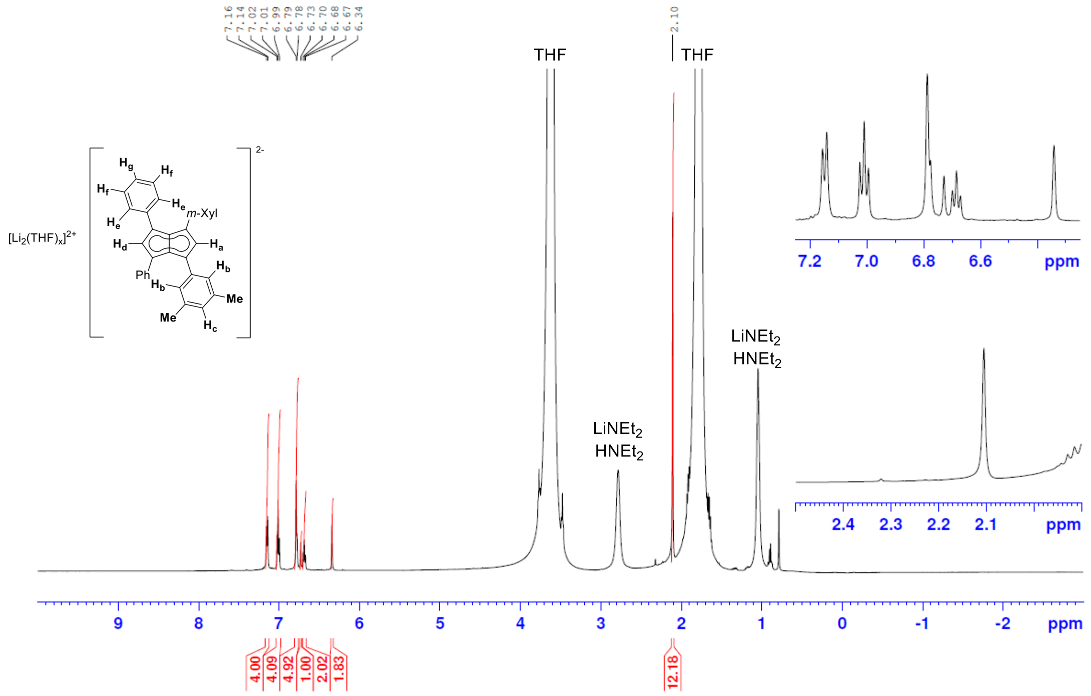

Figure S23: ^{1}H NMR dilithium 1,3-diphenyl-4,6-di-*m*-xylylpentalenide Li_2[**6**] (500 MHz, THF-h_8, 298 K).

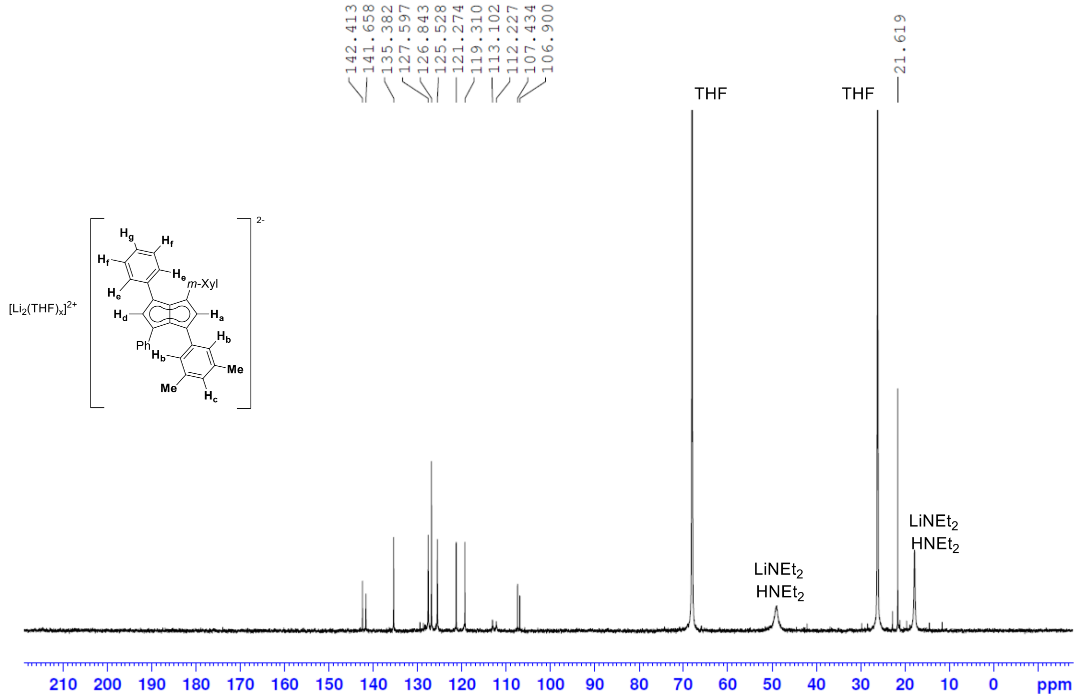

Figure S24: $^{13}C\{^{1}H\}$ NMR dilithium 1,3-diphenyl-4,6-di-*m*-xylylpentalenide Li_2[**6**]
(126 MHz, THF-h_8, 298 K).

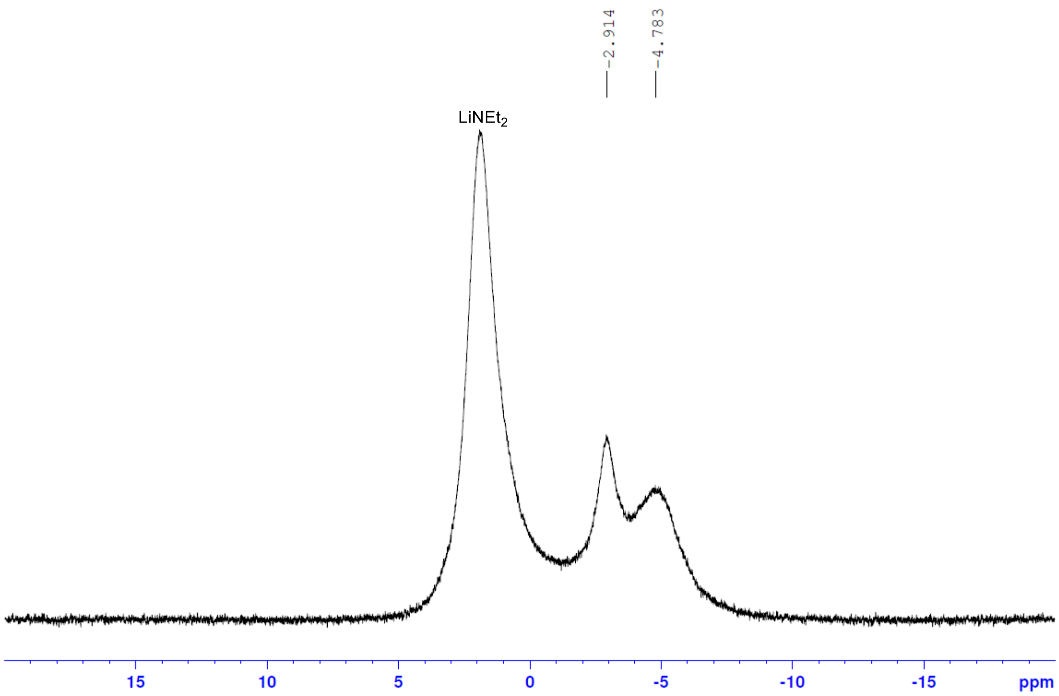

Figure S25: ^{7}Li NMR dilithium 1,3-diphenyl-4,6-di-*m*-xylylpentalenide Li_2[**6**] (194 MHz, THF-h_8, 298 K).

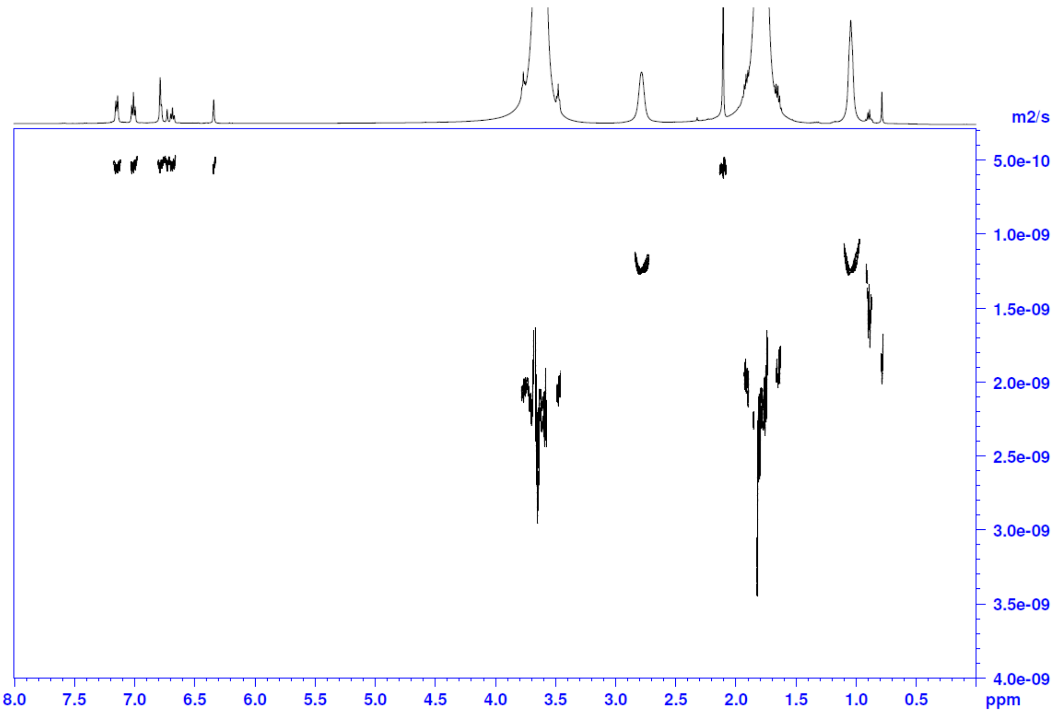

Figure S26: ^{1}H DOSY dilithium 1,3-diphenyl-4,6-di-*m*-xylylpentalenide Li_2[**6**] (500 MHz, THF-h_8, 298 K).

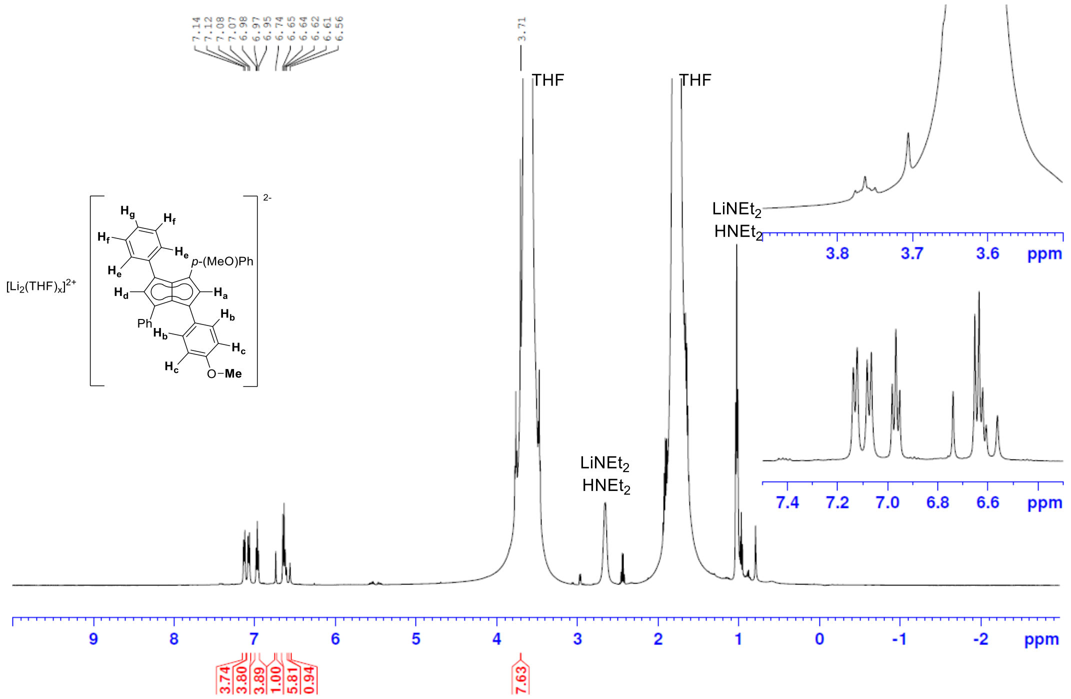

Figure S27: ^{1}H NMR dilithium 1,3-bis(4-methoxyphenyl)-4,6-diphenylpentalenide Li_2[**7**] (500 MHz, THF-h_8, 298 K).

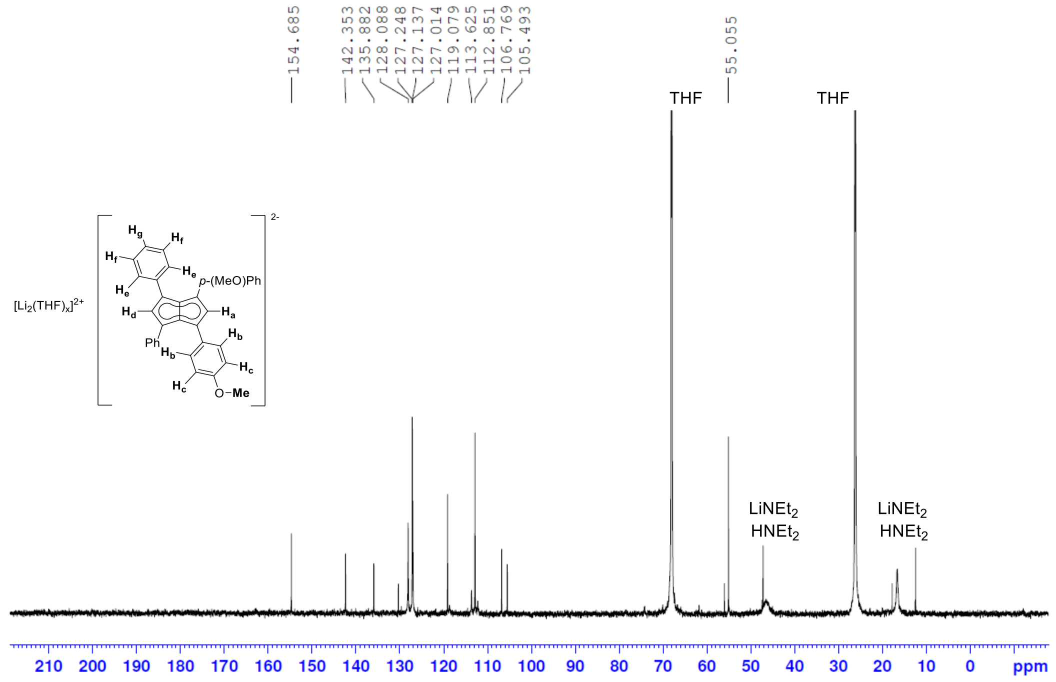

Figure S28: ^{13}C{^{1}H} NMR dilithium 1,3-bis(4-methoxyphenyl)-4,6-diphenylpentalenide Li_2[**7**] (126 MHz, THF-h_8, 298 K).

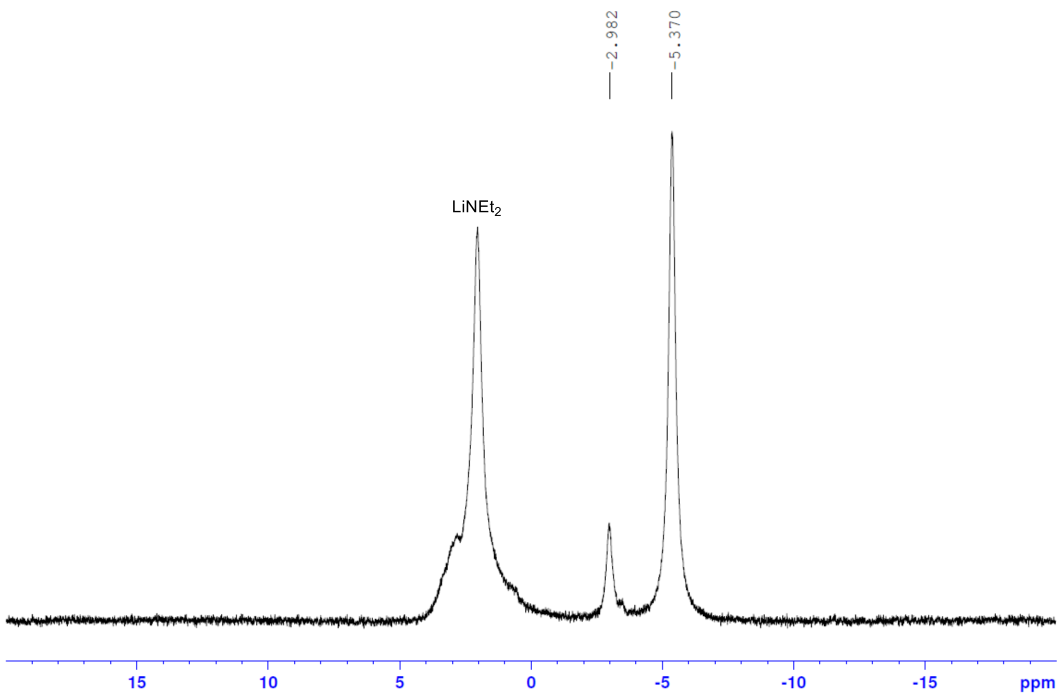

Figure S29: ^{7}Li NMR dilithium 1,3-bis(4-methoxyphenyl)-4,6-diphenylpentalenide Li_2[**7**] (194 MHz, THF-h_8, 298 K).

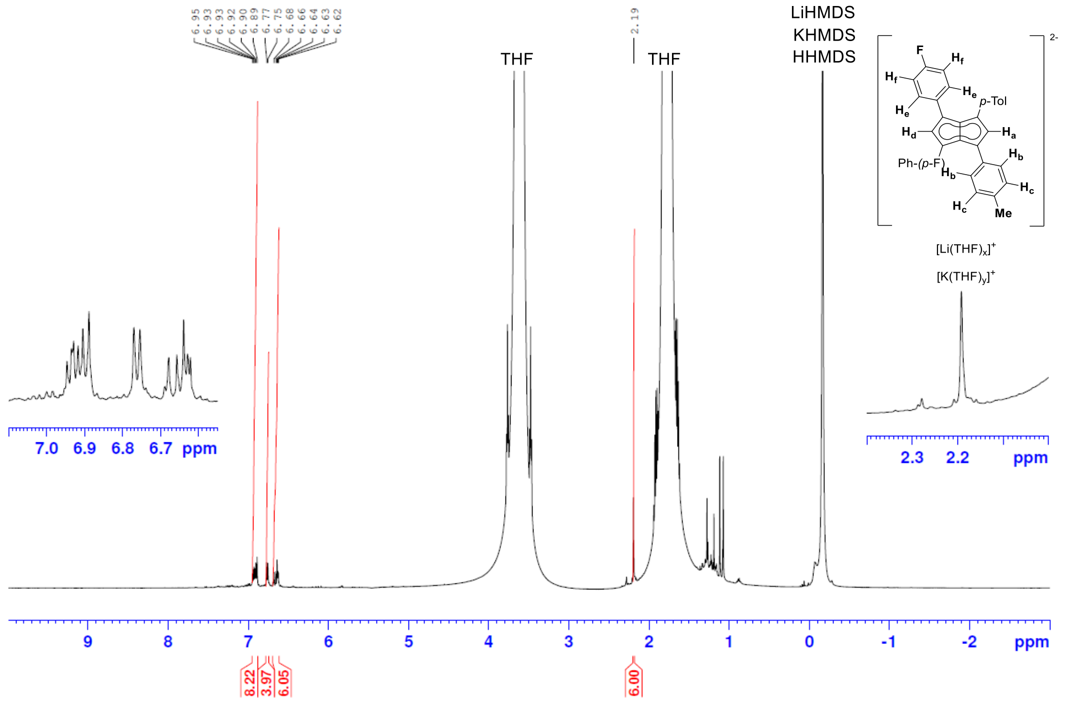

Figure S30: ^{1}H NMR lithium potassium 1,3-bis(4-fluorophenyl)-4,6-di-*p*-tolylpentalenide LiK[**8**] (500 MHz, THF-h_8, 298 K).

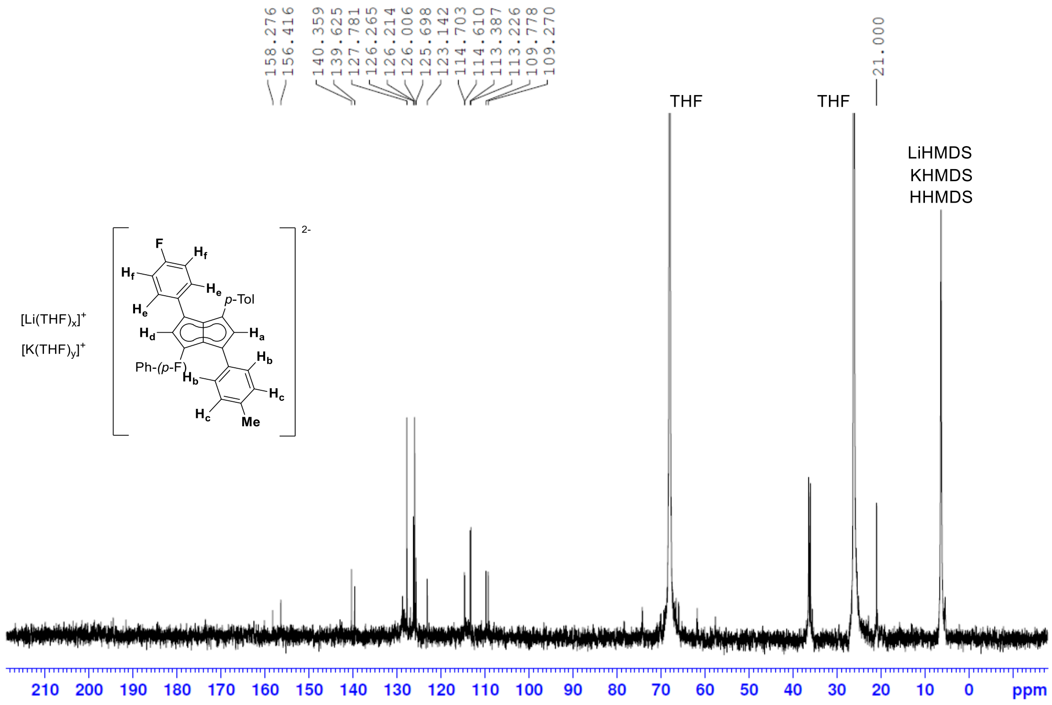

Figure S31: $^{13}C\{^{1}H\}$ NMR lithium potassium 1,3-bis(4-fluorophenyl)-4,6-di-*p*-tolylpentalenide LiK[**8**] (126 MHz, THF-h_8, 298 K).

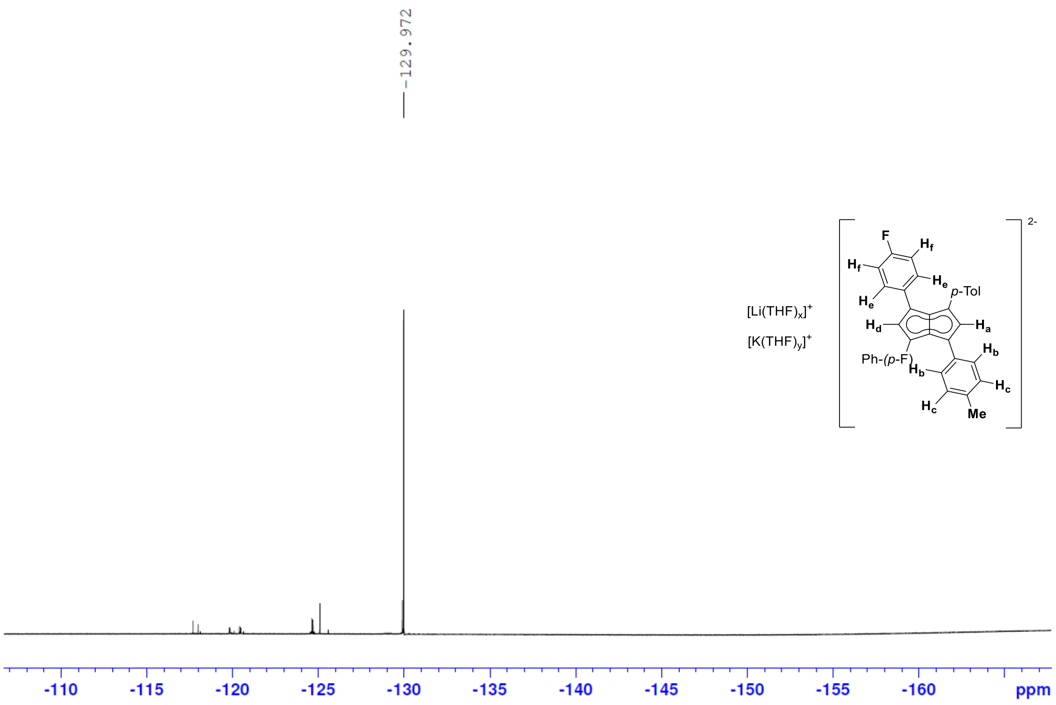

Figure S32: $^{19}F\{^{1}H\}$ NMR lithium potassium 1,3-bis(4-fluorophenyl)-4,6-di-*p*-tolylpentalenide LiK[**8**] (471 MHz, THF-h_8, 298 K).

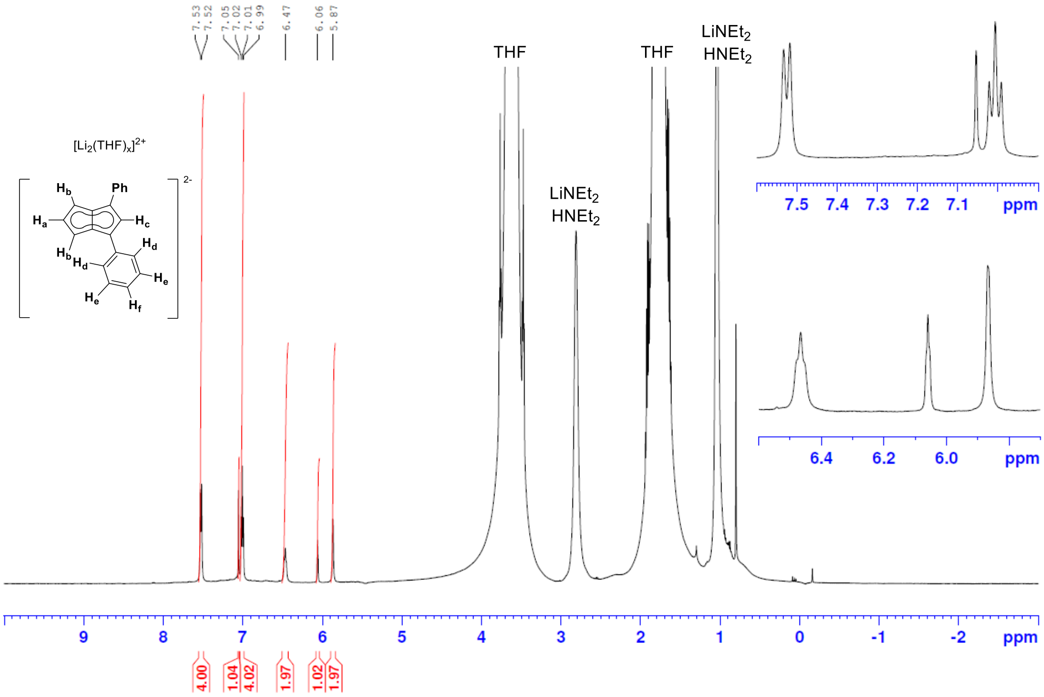

Figure S33: ^{1}H NMR dilithium 1,3-diphenylpentalenide Li_2[**9**]
(500 MHz, THF-h_8, 298 K).

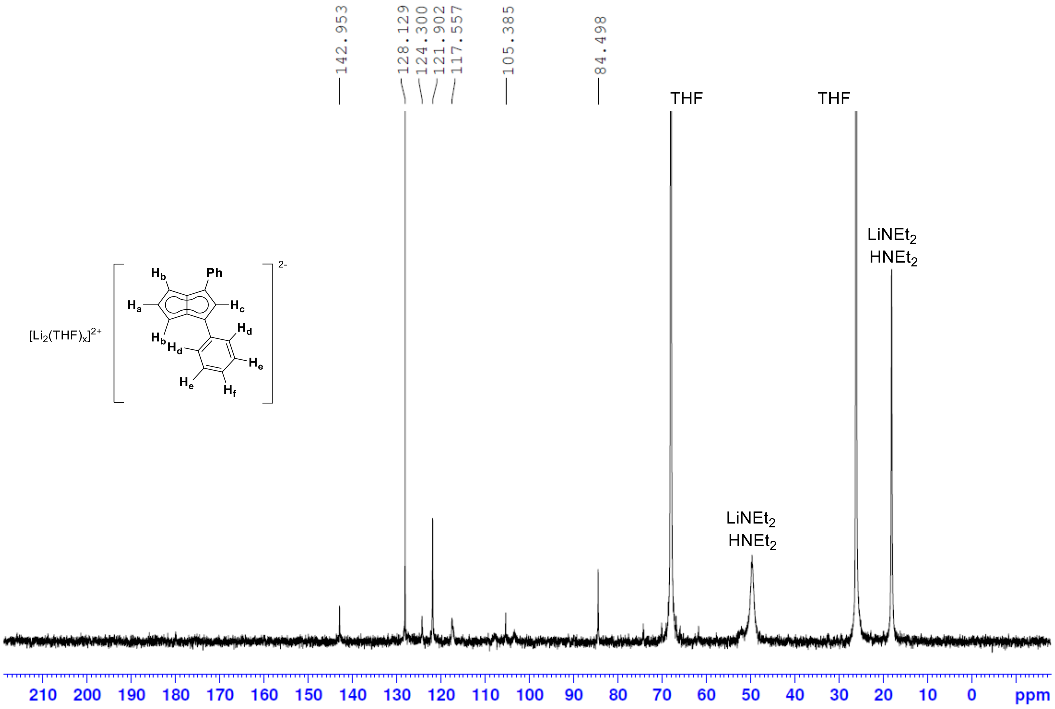

Figure S34: $^{13}C\{^{1}H\}$ NMR dilithium 1,3-diphenylpentalenide Li_2[**9**]
(126 MHz, THF-h_8, 298 K).

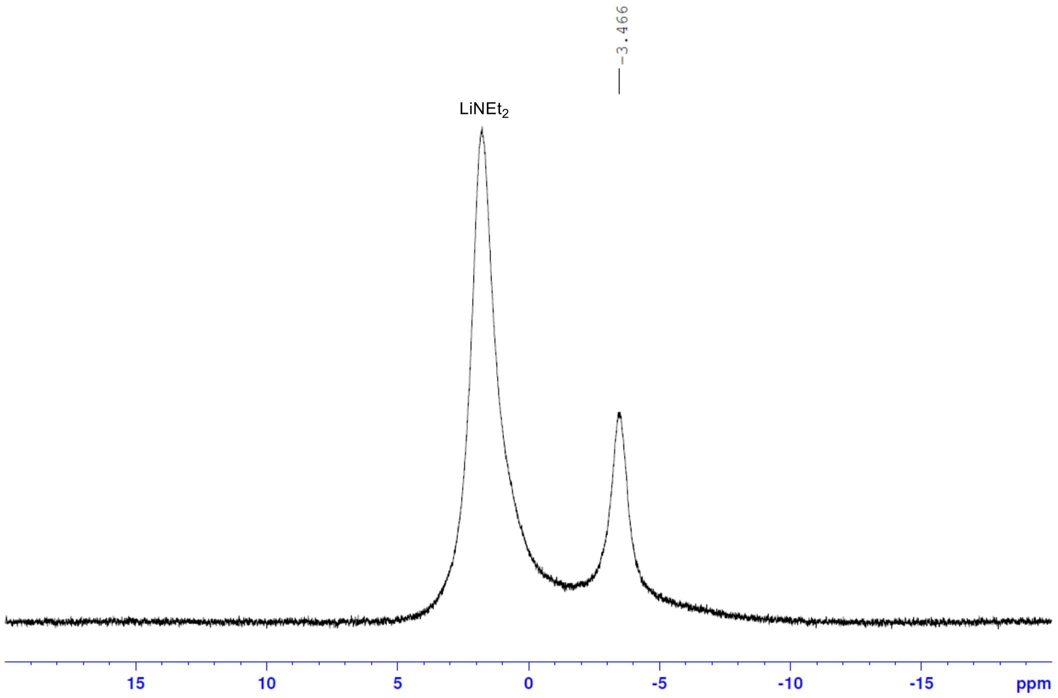

Figure S35: ^{7}Li NMR dilithium 1,3-diphenylpentalenide Li_2[**9**]
(194 MHz, THF-h_8, 298 K).

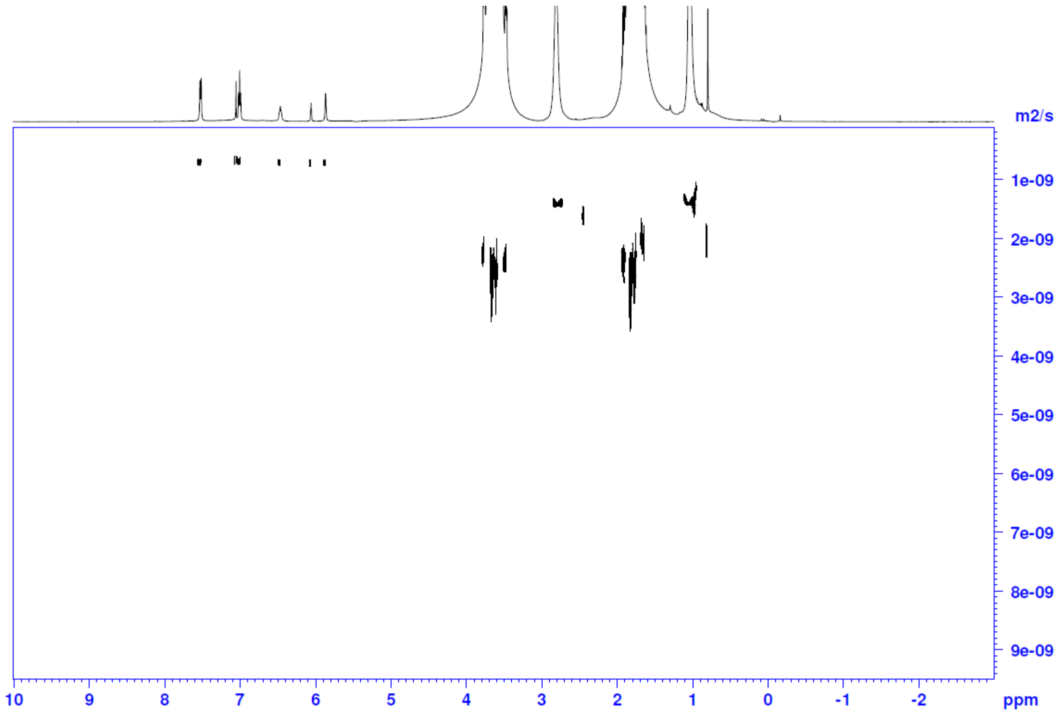

Figure S36: ^{1}H DOSY dilithium 1,3-diphenylpentalenide Li_2[**9**] (500 MHz, THF-h_8, 298 K).

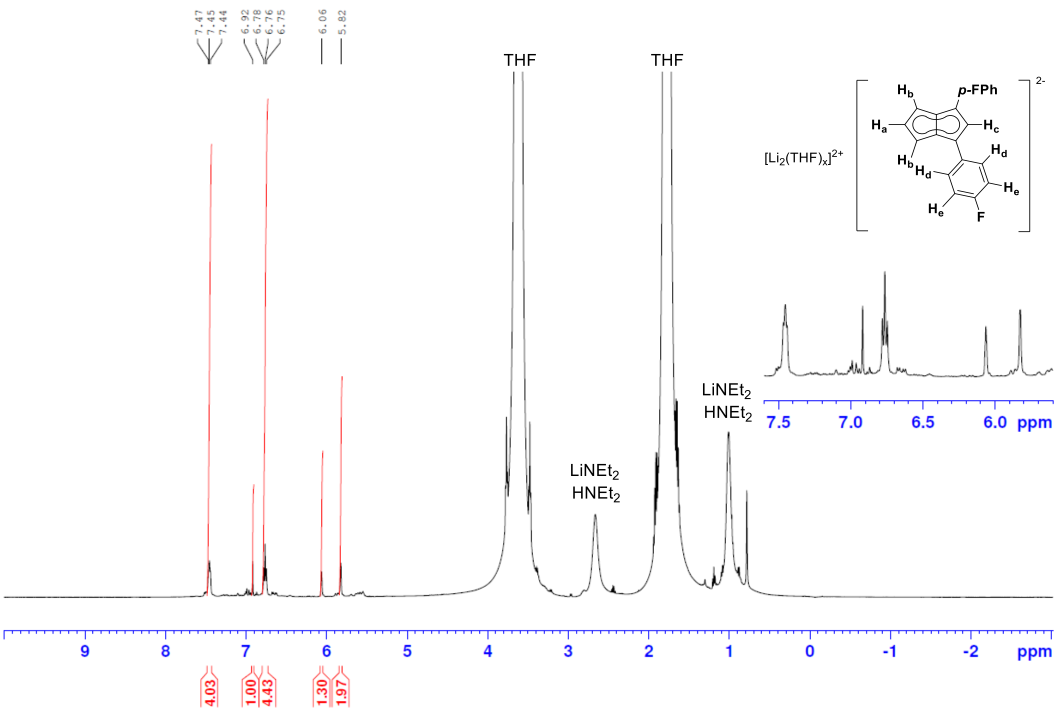

Figure S37: ^{1}H NMR dilithium 1,3-bis(4-fluorophenyl)pentalenide Li$_2$[**10**] (500 MHz, THF-h$_8$, 298 K).

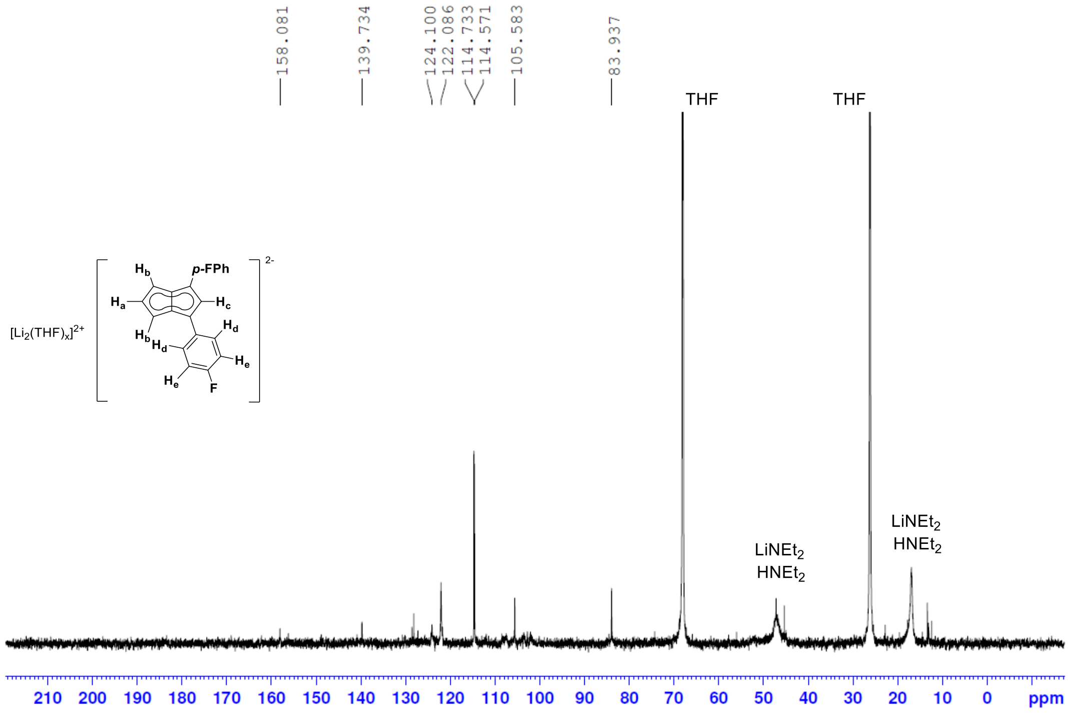

Figure S38: ^{13}C{^{1}H} NMR dilithium 1,3-bis(4-fluorophenyl)pentalenide Li$_2$[**10**] (126 MHz, THF-h$_8$, 298 K).

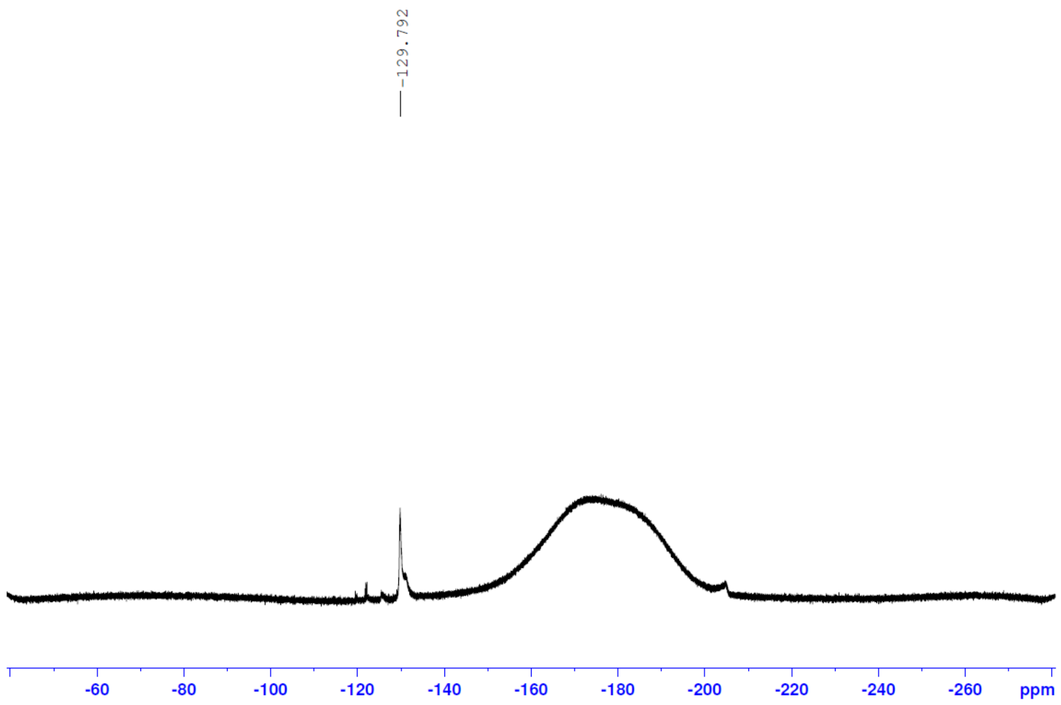

Figure S39: $^{19}F\{^{1}H\}$ NMR dilithium 1,3-bis(4-fluorophenyl)pentalenide Li_2[**10**]
(471 MHz, THF-h_8, 298 K).

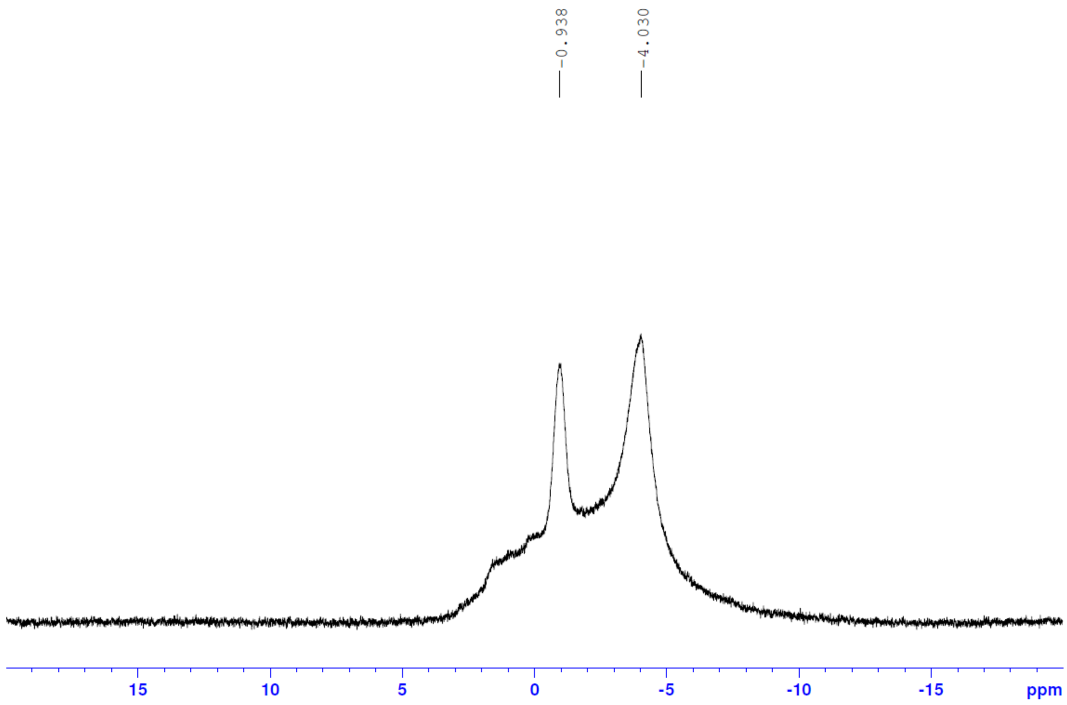

Figure S40: ^{7}Li NMR dilithium 1,3-bis(4-fluorophenyl)pentalenide Li_2[**10**]
(194 MHz, THF-h_8, 298 K).

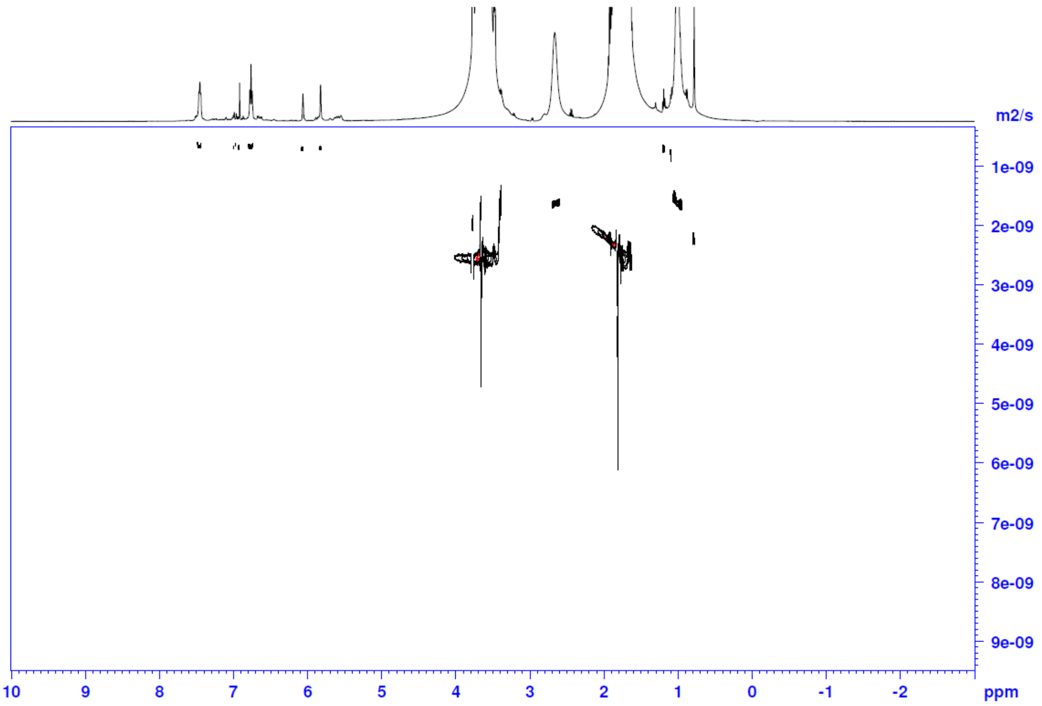

Figure S41: ^{1}H DOSY dilithium 1,3-bis(4-fluorophenyl)pentalenide Li_2[**10**] (500 MHz, THF-h_8, 298 K).

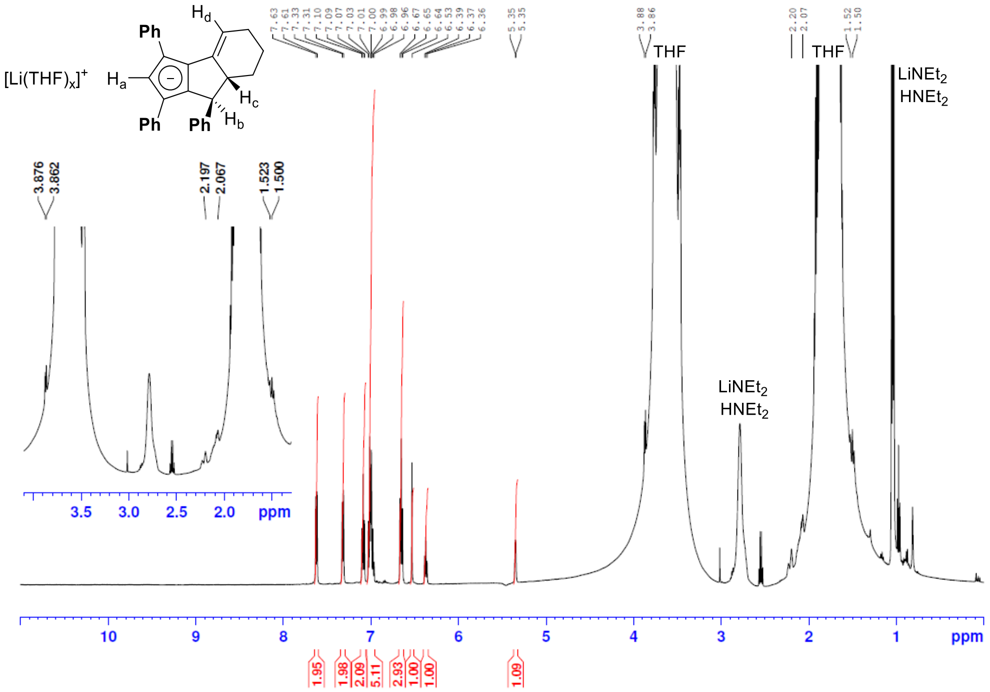

Figure S42: ^{1}H NMR of Li[**11-exo**] (500 MHz, THF-h_8, 298 K).

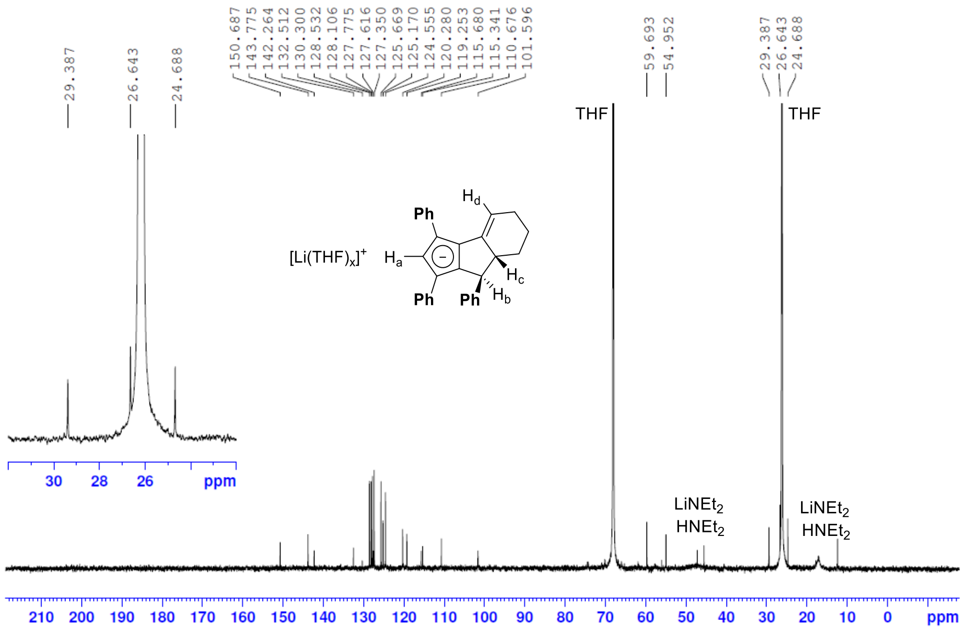

Figure S43: $^{13}C\{^{1}H\}$ NMR of Li[**11-exo**] (126 MHz, THF-h_8, 298 K).

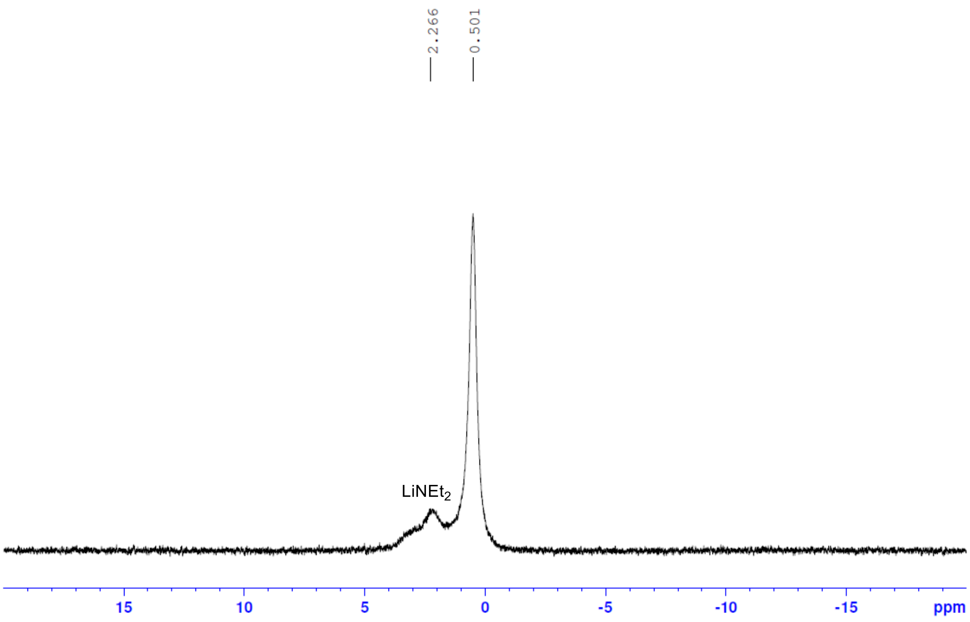

Figure S44: ^{7}Li NMR of Li[**11-exo**] (194 MHz, THF-h_8, 298 K).

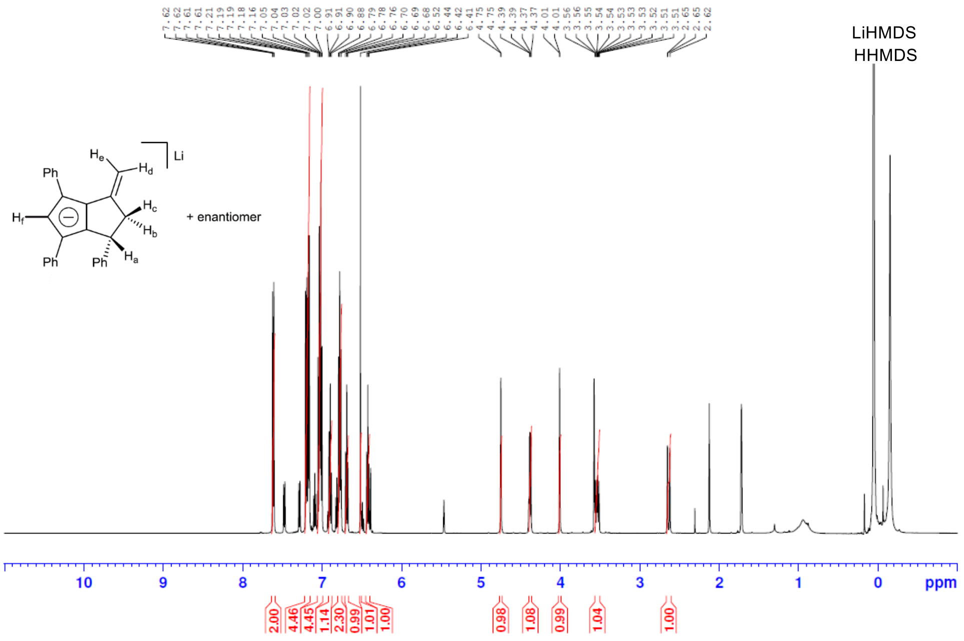

Figure S45: ^{1}H NMR lithium 3-vinyl-1,4,6-triphenyl-1,2,2-trihydropentalenide Li[**12-exo**] (500 MHz, THF-d_8, 298 K).

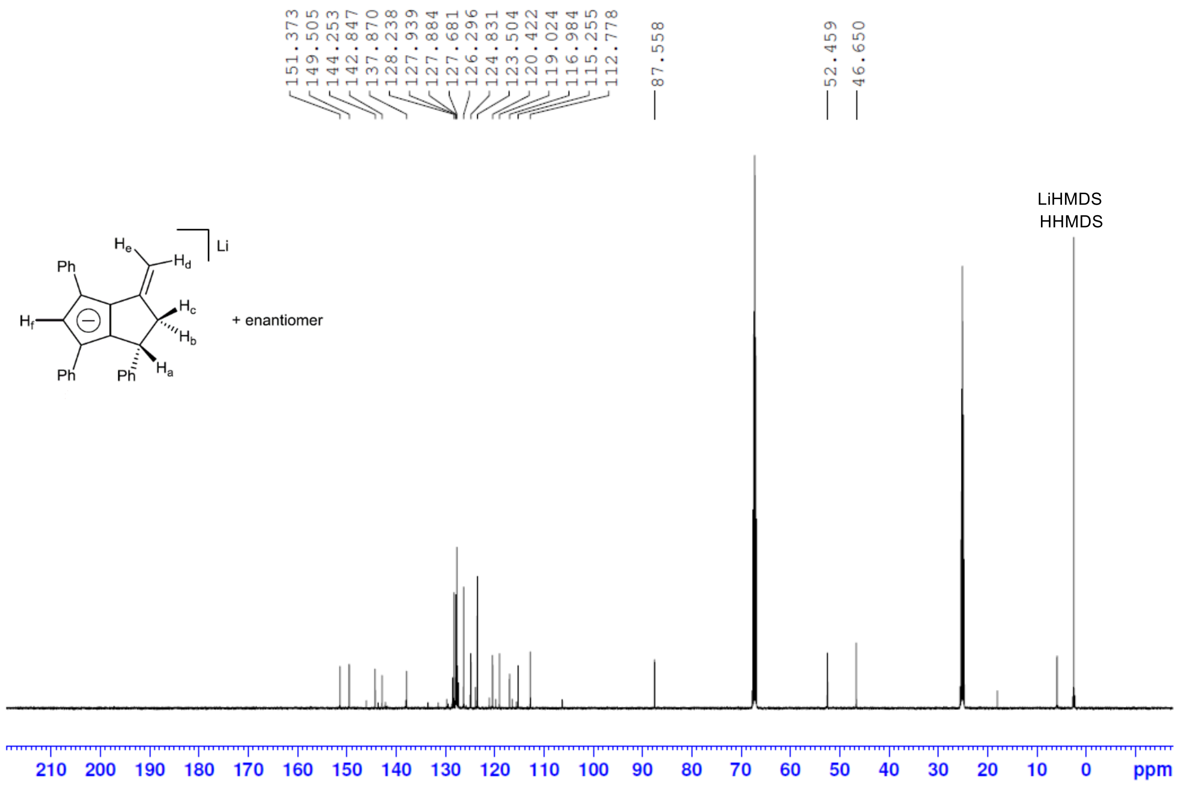

Figure S46: ^{13}C{^{1}H} NMR lithium 3-vinyl-1,4,6-triphenyl-1,2,2-trihydropentalenide Li[**12-exo**] (126 MHz, THF-d_8, 298 K).

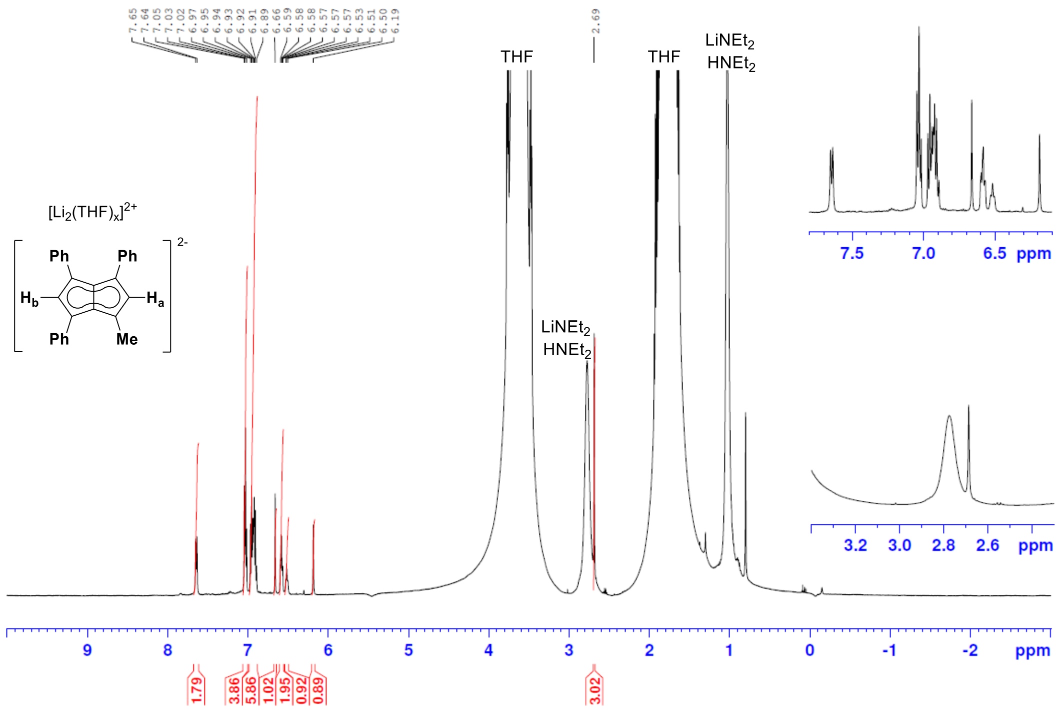

Figure S47: ^{1}H NMR dilithium 1-methyl-3,4,6-triphenylpentalenide Li_2[**12**]
(500 MHz, THF-h_8, 298 K).

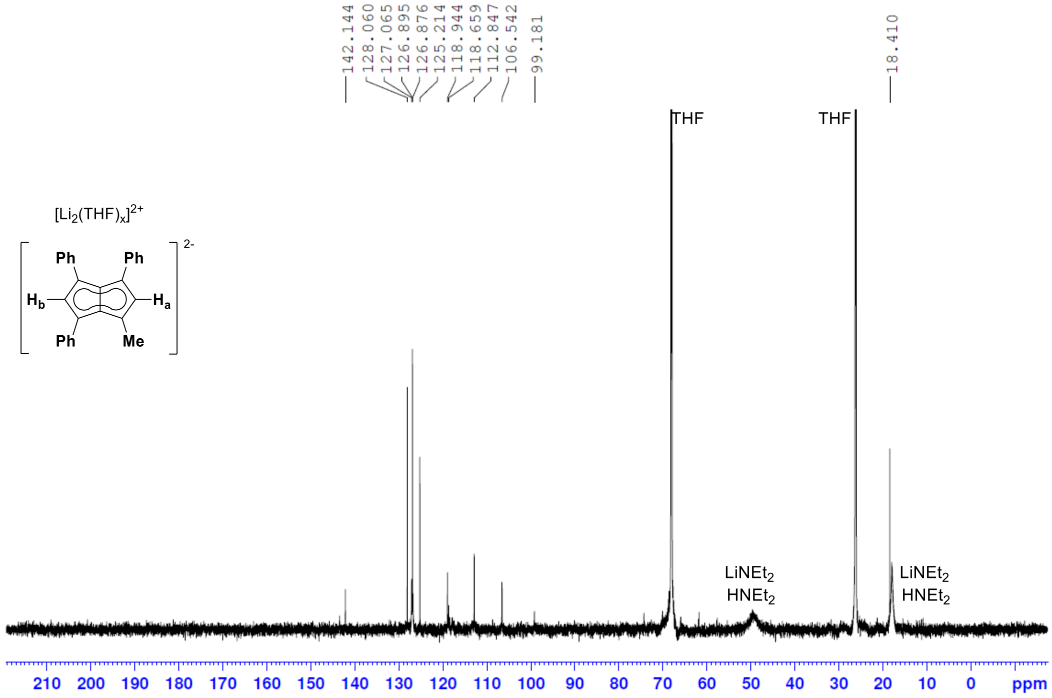

Figure S48: ^{13}C{^{1}H} NMR dilithium 1-methyl-3,4,6-triphenylpentalenide Li_2[**12**]
(126 MHz, THF-h_8, 298 K).

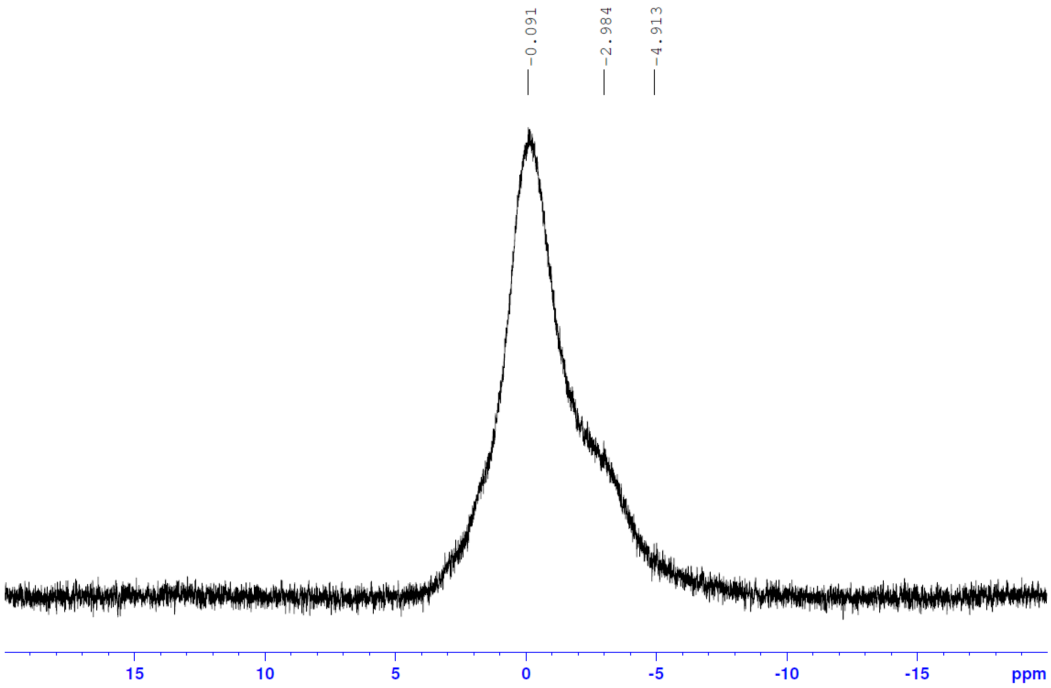

Figure S49: ^{7}Li NMR dilithium 1-methyl-3,4,6-triphenylpentalenide Li_2[**12**]
(194 MHz, THF-h_8, 298 K).

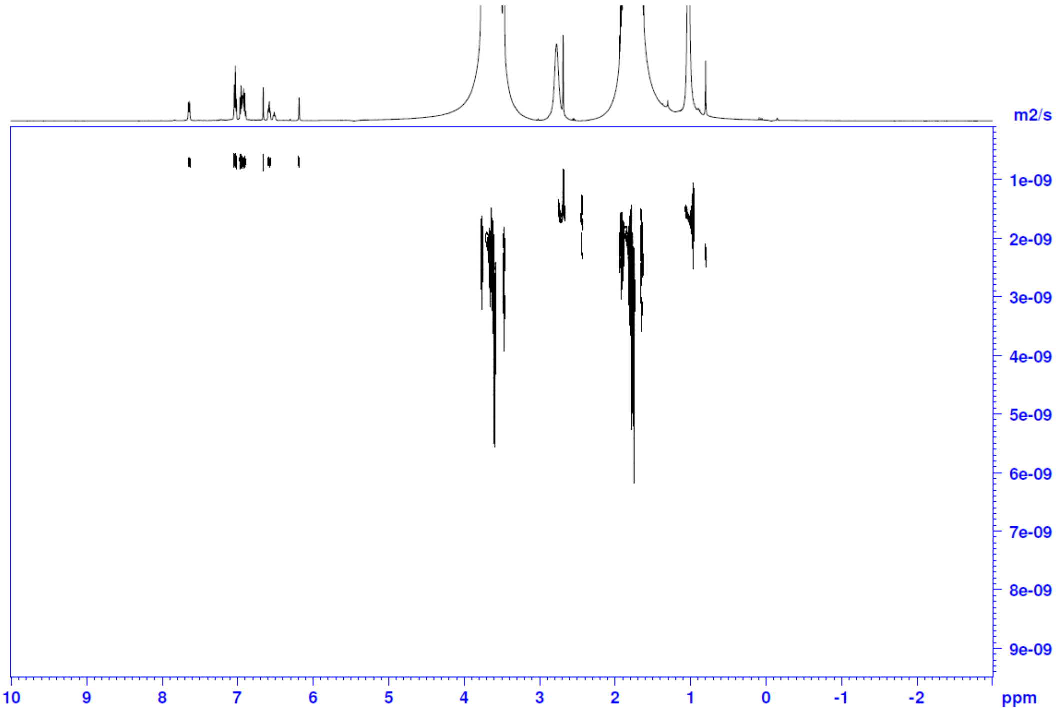

Figure S50: ^{1}H DOSY dilithium 1-methyl-3,4,6-triphenylpentalenide Li_2[**12**]
(500 MHz, THF-h_8, 298 K).

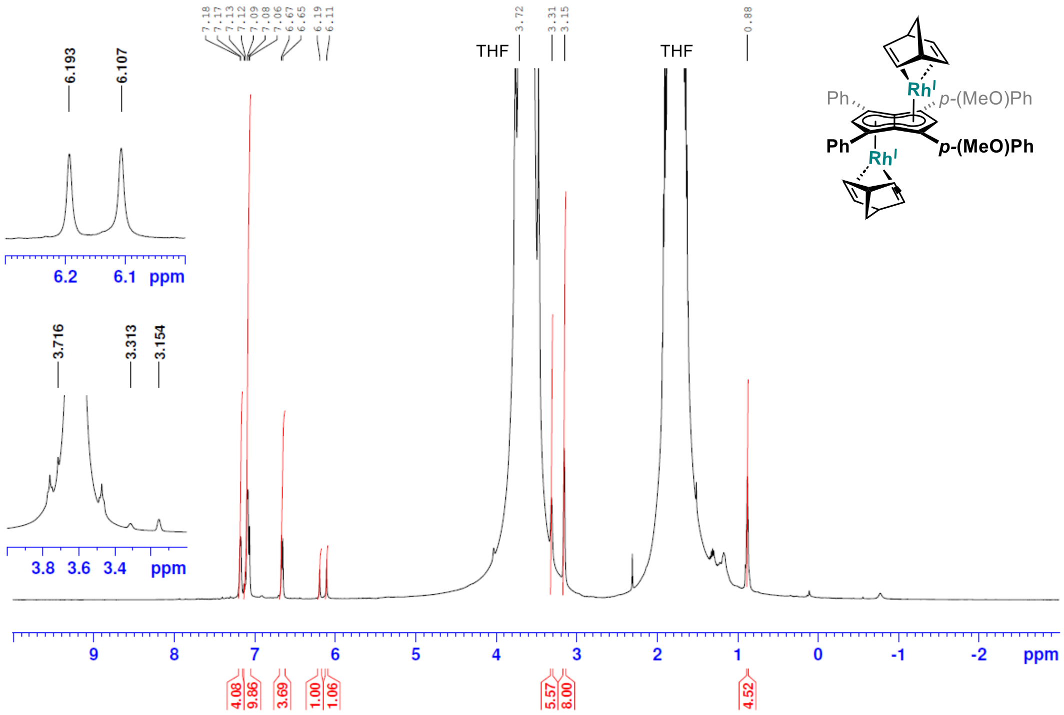

Figure S51: ^{1}H NMR *anti*-bis(2,5-Norbornadiene)dirhodium 1,3-bis(4-methoxyphenyl)-4,6-diphenyl-pentalenide Rh_2NBD_2[**7**] (500 MHz, THF-h_8, 298 K).

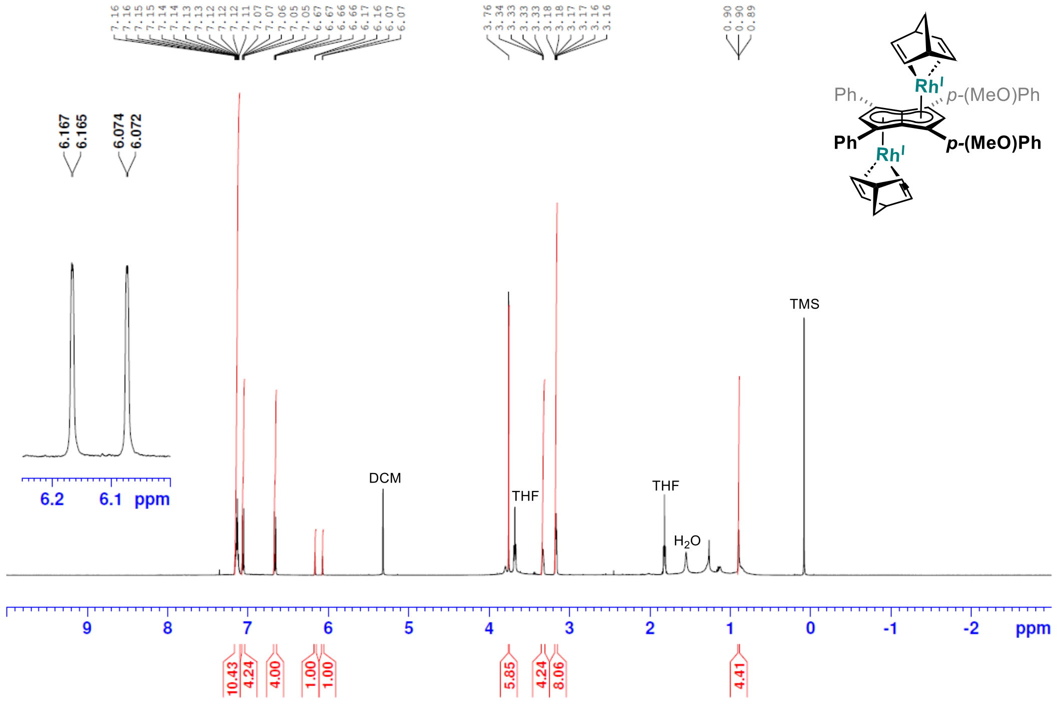

Figure S52: ^{1}H NMR *anti*-bis(2,5-Norbornadiene)dirhodium 1,3-bis(4-methoxyphenyl)-4,6-diphenyl-pentalenide Rh_2NBD_2[**7**] (500 MHz, DCM-d_2, 298 K).

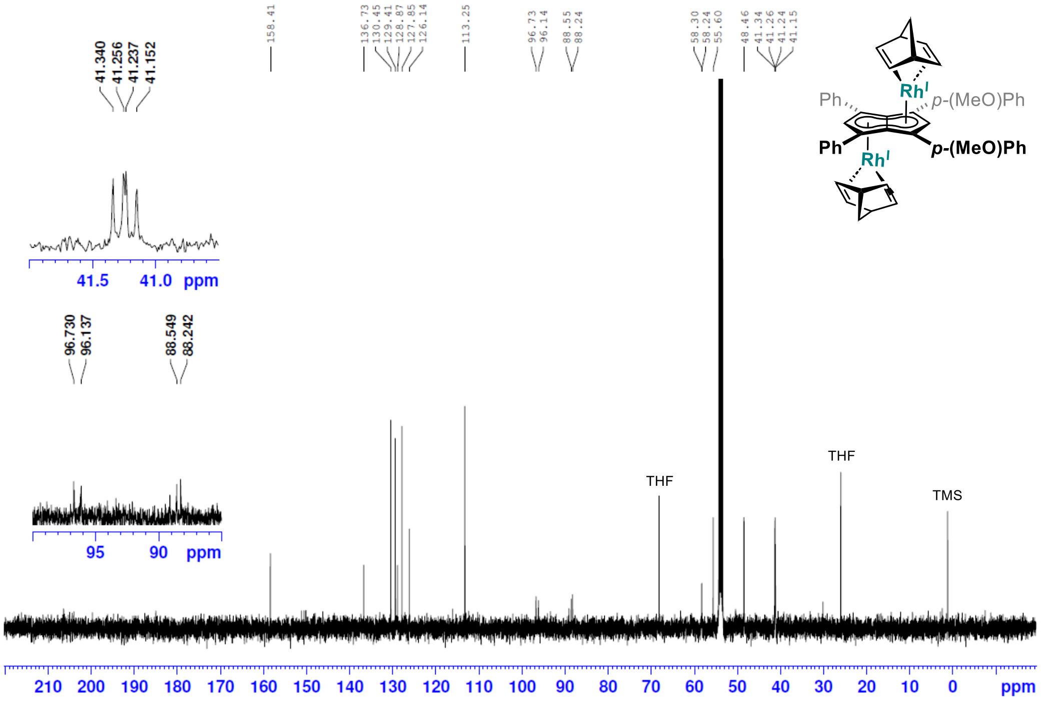

Figure S53: $^{13}C\{^1H\}$ NMR *anti*-bis(2,5-Norbornadiene)dirhodium 1,3-bis(4-methoxyphenyl)-4,6-diphenyl-pentalenide Rh_2NBD_2[**7**] (126 MHz, DCM-d_2, 298 K).

6. Variable temperature NMR of potassium lithium 1,3,4,6-tetrakis(3,5-dimethylphenyl)-pentalenide KLi[**4**] (Figures S54–S57)

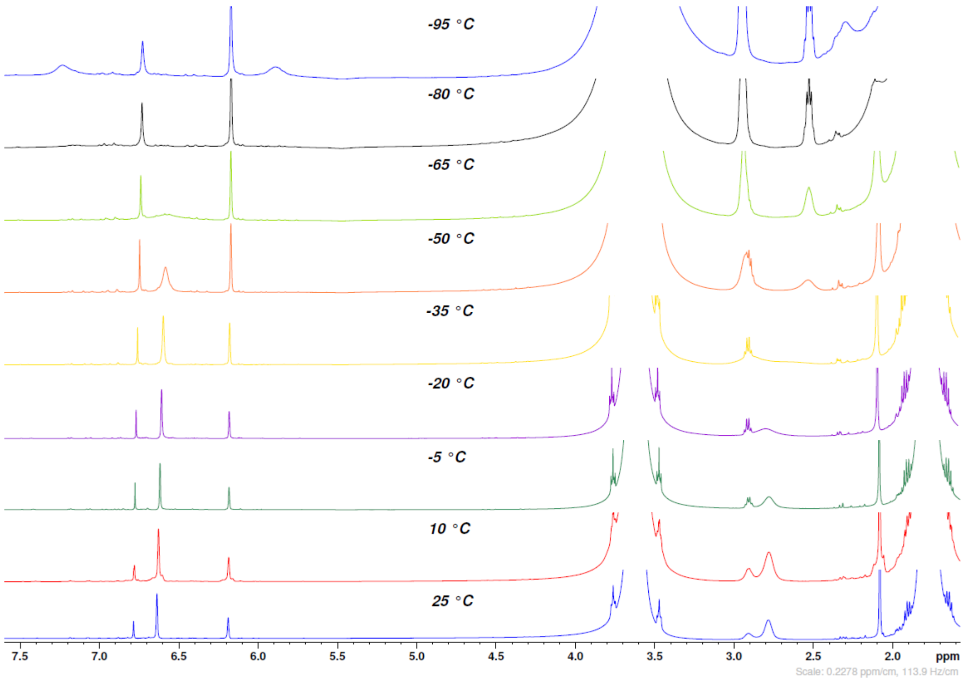

Figure S54: ^{1}H VT NMR of potassium lithium 1,3,4,6-tetrakis(3,5-dimethylphenyl)pentalenide KLi[**4**] (500 MHz, THF-h_8).

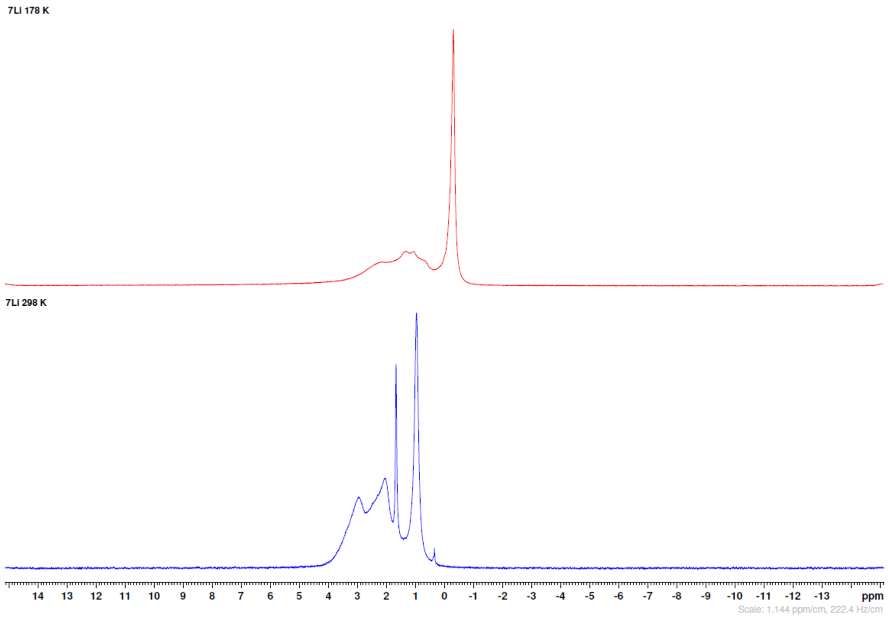

Figure S55: ^{7}Li VT NMR of potassium lithium 1,3,4,6-tetrakis(3,5-dimethylphenyl)pentalenide KLi[**4**] (194 MHz, THF-h_8).

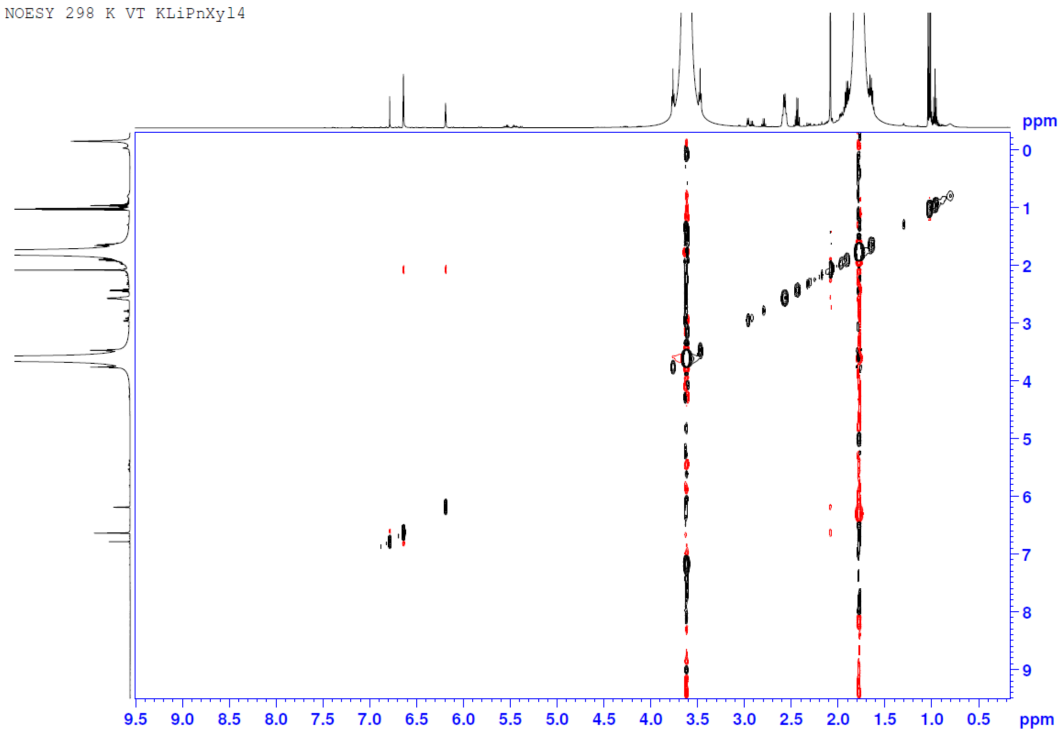

Figure S56: ^{1}H NOESY of potassium lithium 1,3,4,6-tetrakis(3,5-dimethylphenyl)pentalenide KLi[**4**] (500 MHz, 298 K, THF-h_8).

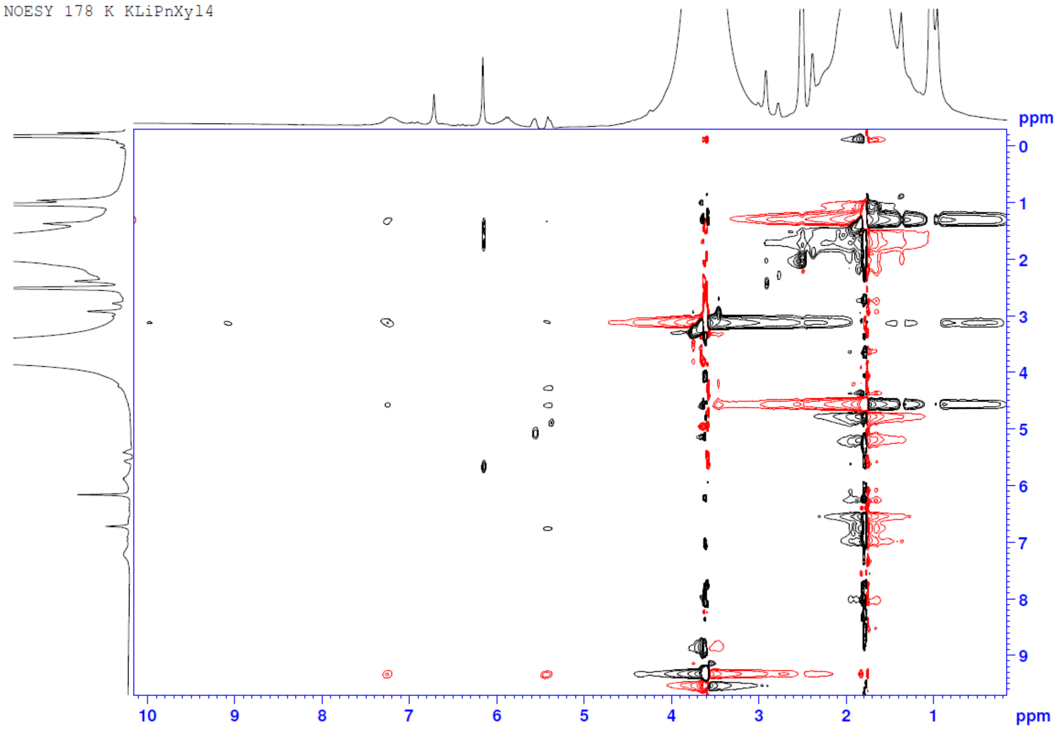

Figure S57: ^{1}H NOESY of potassium lithium 1,3,4,6-tetrakis(3,5-dimethylphenyl)pentalenide KLi[**4**] (500 MHz, 178 K, THF-h_8).

7. NMR spectra of deprotonative metalation attempt of CF_3Ph-substituted dihydropentalenes (Figures S58 and S59)

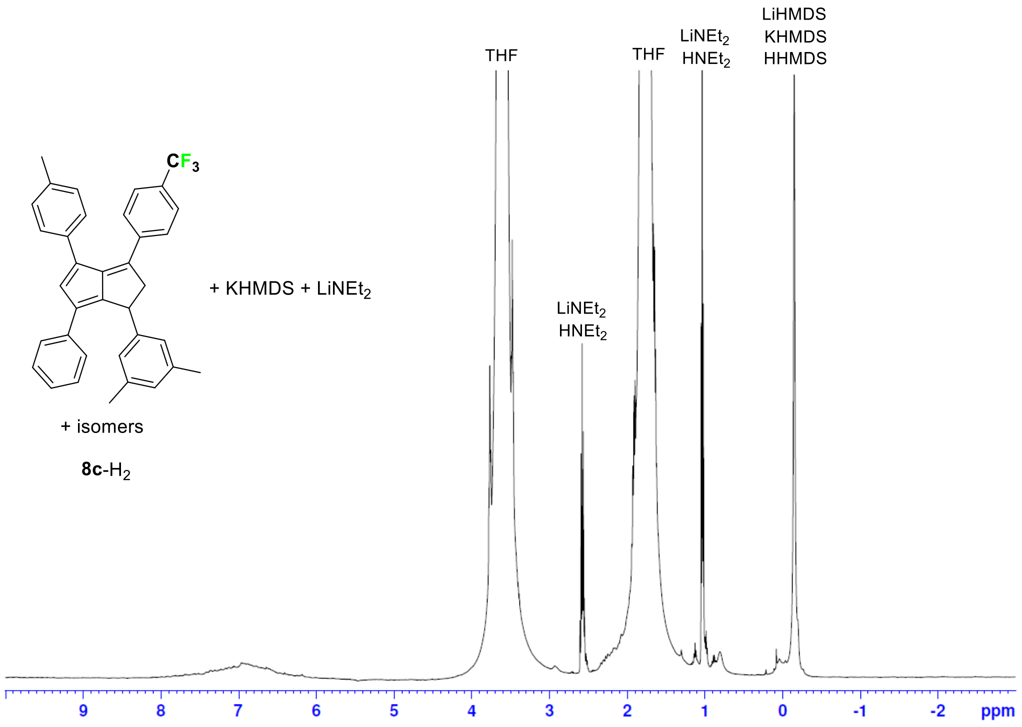

Figure S58: ^{1}H NMR of deprotonative metalation attempt of **8c**-H_2 with KHMDS and $LiNEt_2$

(500 MHz, THF-h_8, 298 K).

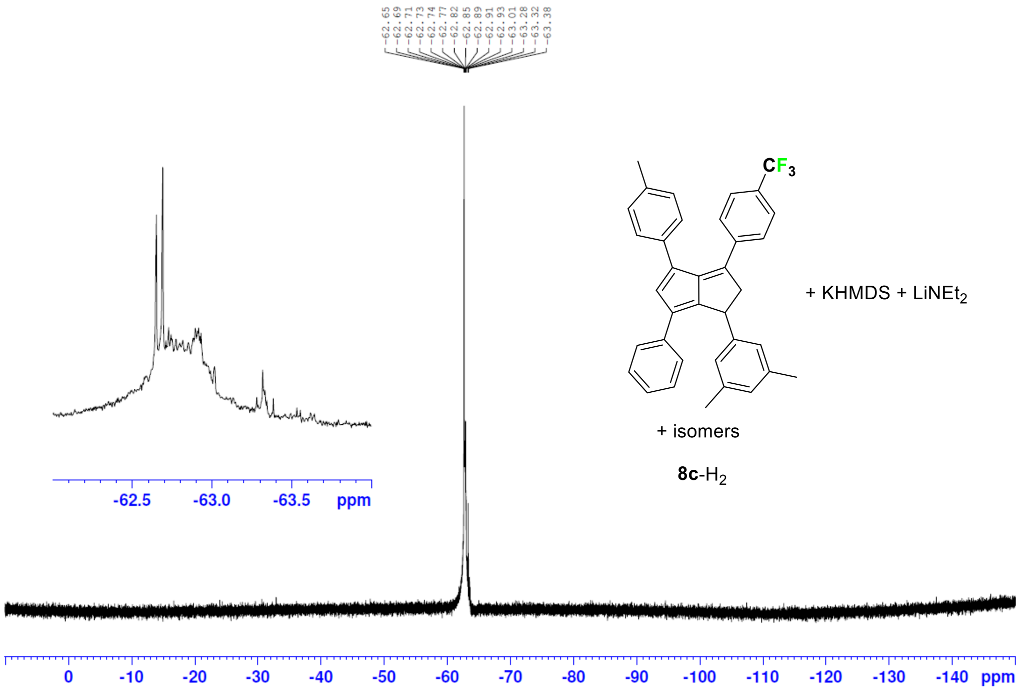

Figure S59: $^{19}F\{^{1}H\}$ NMR of deprotonative metalation attempt of **8c**-H_2 (471 MHz, THF-h_8, 298 K).

References for experimental chapters:

1. N. A. Jenek, M. Balschun, S. M. Boyt and U. Hintermair, *J. Org. Chem.*, 2022, **87**, 13790–13802.
2. N. L. Drake and J. R. Adams, *Journal of the American Chemical Society*, 1939, **61**, 1326–1329.
3. S. M. Boyt, N. A. Jenek, H. J. Sanderson, G. Kociok-Kohn and U. Hintermair, *Organometallics*, 2022, **41**, 211–225.
4. L. D. Hicks, A. J. Fry and V. C. Kurzweil, *Electrochim. Acta*, 2004, **50**, 1039–1047.
5. A. G. Griesbeck, *Chem. Ber.*, 1991, **124**, 403–405.
6. P. A. Boeg, J. Ø. Duus, J. H. Ardenkjær-Larsen, M. Karlsson and S. Mossin, *J. Phys. Chem. C*, 2019, **123**, 9949–9956.

8. X-ray crystallography (Figures S60–S64)

Crystals were selected using the oil drop technique using perfluoropolyether oil and mounted at 150(2) K with an Oxford Cryostream N_2 open-flow cooling device. Intensity data were collected on a Rigaku SuperNova Dual EosS2 single crystal diffractometer using monochromated Cu-Kα radiation (λ = 1.54184 Å). Unit cell determination, data collection, data reduction and absorption correction were performed using the CrysAlisPro software[1]. The structures were solved with SHELXT[2] and refined by a full-matrix least-squares procedure based on F^2 (SHELXL-2018/3)[2]. All non-hydrogen atoms were refined anisotropically. Hydrogen atoms were placed onto calculated positions and refined using a riding model.

Additional programmes used for analysing data and their graphical manipulation included:

SHELXle[3], ORTEP3 for windows [4] and Mercury[5]

1. CrysAlisPro 1.171.42.49 (Rigaku Oxford Diffraction, 2022).
2. SHELXL: G. M. Sheldrick, ActaCryst, 2015, C71, 3-8.
3. ShelXle: a Qt graphical user interface for SHELXL: C. B. Hübschle, G. M. Sheldrick and B. Dittrich, J. Appl. Cryst., 44, (2011) 1281-1284.
4. ORTEP3 for Windows L. J. Farrugia, J. Appl. Crystallogr. 1997, 30, 565.
5. Mercury: C. F. Macrae, P. R. Edgington, P. McCabe, E. Pidcock, G. P. Shields, R. Taylor, Towler and van der Streek, J. Appl. Crystallogr., 2006, 39, 453-457.

8.1. Crystal data and structure refinement for disodium 1,3,4,6-*para*-tolylpentalenide (Na_2[**3**])

**CCDC** number	**2281041**	
Identification code	s22uh31	
Empirical formula	C_{60} H_{78} Na_2 O_6	
Formula weight	941.20	
Temperature	150.00(10) K	
Wavelength	1.54184 Å	
Crystal system	Monoclinic	
Space group	$P2_1/n$	
Unit cell dimensions	a = 11.56222(7) Å	α = 90°
	b = 12.72345(7) Å	β = 93.4414(5)°
	c = 18.05699(10) Å	γ = 90°
Volume	2651.60(3) $Å^3$	
Z	2	
Density (calculated)	1.179 Mg/m^3	
Absorption coefficient	0.720 mm^{-1}	
F(000)	1016	
Crystal size	0.613 x 0.582 x 0.344 mm^3	
Theta range for data collection	4.253 to 72.894°.	
Index ranges	-14<=h<=13, -15<=k<=15, -22<=l<=22	
Reflections collected	57489	
Independent reflections	5290 [R(int) = 0.0317]	
Completeness to theta = 67.684°	99.9 %	
Absorption correction	Semi-empirical from equivalents	
Max. and min. transmission	1.00000 and 0.70894	
Refinement method	Full-matrix least-squares on F^2	
Data / restraints / parameters	5290 / 0 / 346	
Goodness-of-fit on F^2	1.043	
Final R indices [I>2sigma(I)]	R1 = 0.0437, wR2 = 0.1145	
R indices (all data)	R1 = 0.0448, wR2 = 0.1154	
Largest diff. peak and hole	0.267 and -0.284 e.$Å^{-3}$	

Datablock s22uh31 - ellipsoid plot

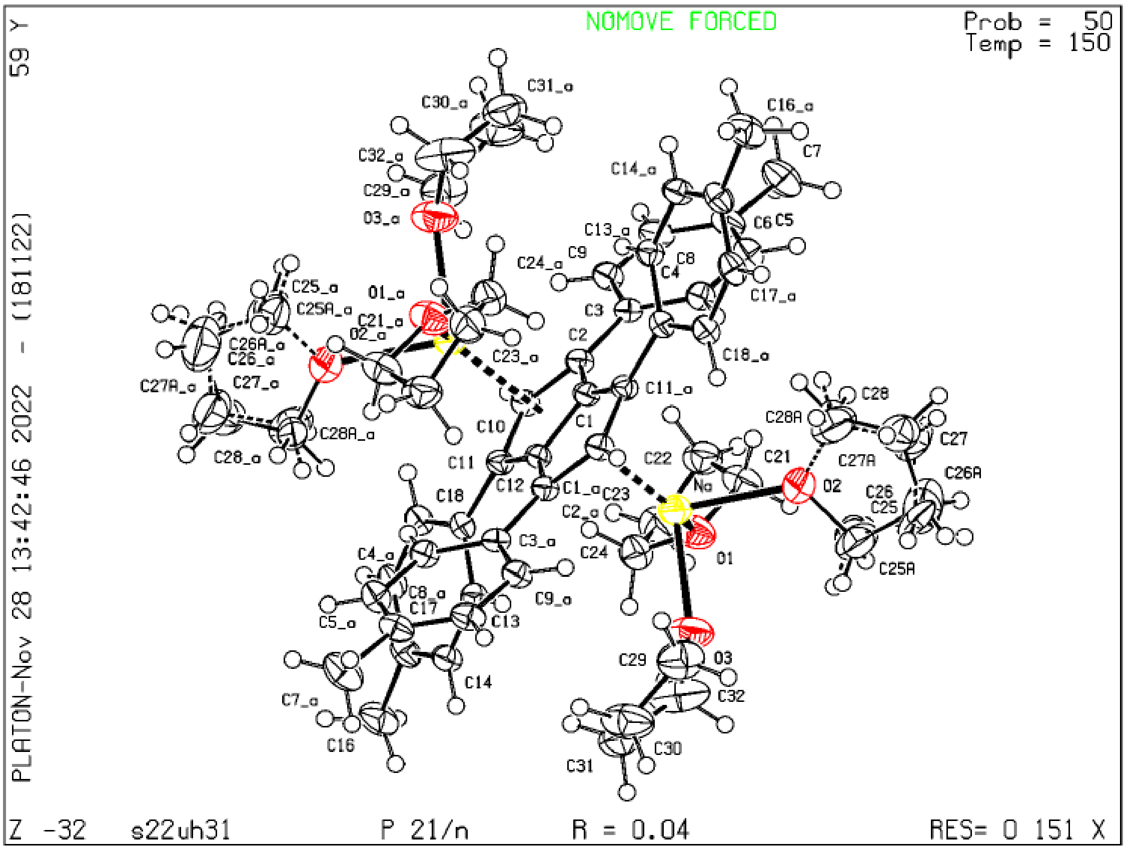

Figure S60: Ellipsoid plot for disodium 1,3,4,6-tetra-*p*-tolylpentalenide Na_2[**3**].

8.2. Crystal data and structure refinement for 1-methyl-3,4,6-triphenyl-1,2-dihydropentalene (**12'**H_2)

**CCDC** number	**2281042**	
Identification code	s22uh8	
Empirical formula	C_{27} H_{22}	
Formula weight	346.44	
Temperature	150.00(10) K	
Wavelength	1.54184 Å	
Crystal system	Monoclinic	
Space group	$P2_1$	
Unit cell dimensions	a = 9.0055(2) Å	α = 90°
	b = 8.75137(16) Å	β = 97.095(2)°
	c = 12.0243(3) Å	γ = 90°
Volume	940.38(4) $Å^3$	
Z	2	
Density (calculated)	1.224 Mg/m^3	
Absorption coefficient	0.519 mm^{-1}	
F(000)	368	
Crystal size	0.130 x 0.090 x 0.050 mm^3	
Theta range for data collection	3.704 to 72.817°.	
Index ranges	-10<=h<=11, -10<=k<=10, -14<=l<=14	
Reflections collected	18689	
Independent reflections	3691 [R(int) = 0.0377]	
Completeness to theta = 67.684°	100.0 %	
Absorption correction	Semi-empirical from equivalents	
Max. and min. transmission	1.00000 and 0.81786	
Refinement method	Full-matrix least-squares on F^2	
Data / restraints / parameters	3691 / 1 / 245	
Goodness-of-fit on F^2	1.416	
Final R indices [I>2sigma(I)]	R1 = 0.0375, wR2 = 0.1101	
R indices (all data)	R1 = 0.0417, wR2 = 0.1109	
Absolute structure parameter	0.0(10)	
Extinction coefficient	n/a	

Largest diff. peak and hole 0.199 and -0.239 e.Å^{-3}

Datablock s22uh8 - ellipsoid plot

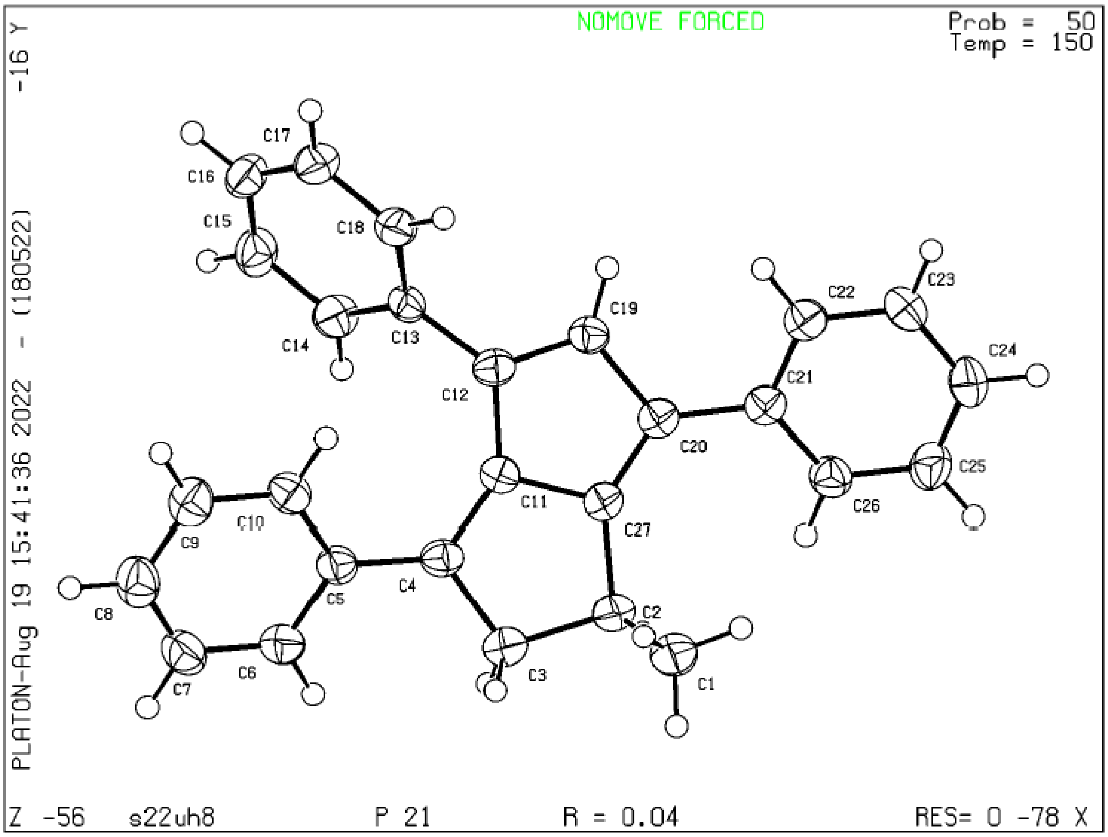

Figure S61: Ellipsoid plot for 1-methyl-3,4,6-triphenyl-1,2-dihydropentalene **12’**H_2.

8.3. Crystal data and structure refinement for 3-methyl-1,4,6-triphenyl-1,2-dihydropentalene (12H_2)

**CCDC** number	**2281043**	
Identification code	s22uh14	
Empirical formula	C_{27} H_{22}	
Formula weight	346.44	
Temperature	150.00(10) K	
Wavelength	1.54184 Å	
Crystal system	Monoclinic	
Space group	$P2_1/n$	
Unit cell dimensions	a = 11.01120(10) Å	α= 90°
	b = 8.76260(10) Å	β= 91.5730(10)°
	c = 19.1326(2) Å	γ = 90°
Volume	1845.35(3) $Å^3$	
Z	4	
Density (calculated)	1.247 Mg/m^3	
Absorption coefficient	0.529 mm^{-1}	
F(000)	736	
Crystal size	0.350 x 0.320 x 0.100 mm^3	
Theta range for data collection	4.580 to 72.923°.	
Index ranges	-13<=h<=13, -10<=k<=9, -23<=l<=23	
Reflections collected	31060	
Independent reflections	3662 [R(int) = 0.0293]	
Completeness to theta = 67.684°	99.8 %	
Absorption correction	Semi-empirical from equivalents	
Max. and min. transmission	1.00000 and 0.81980	
Refinement method	Full-matrix least-squares on F^2	
Data / restraints / parameters	3662 / 0 / 245	
Goodness-of-fit on F^2	1.053	
Final R indices [I>2sigma(I)]	R1 = 0.0360, wR2 = 0.0874	
R indices (all data)	R1 = 0.0391, wR2 = 0.0897	
Largest diff. peak and hole	0.182 and -0.210 e.$Å^{-3}$	

Datablock s22uh14 - ellipsoid plot

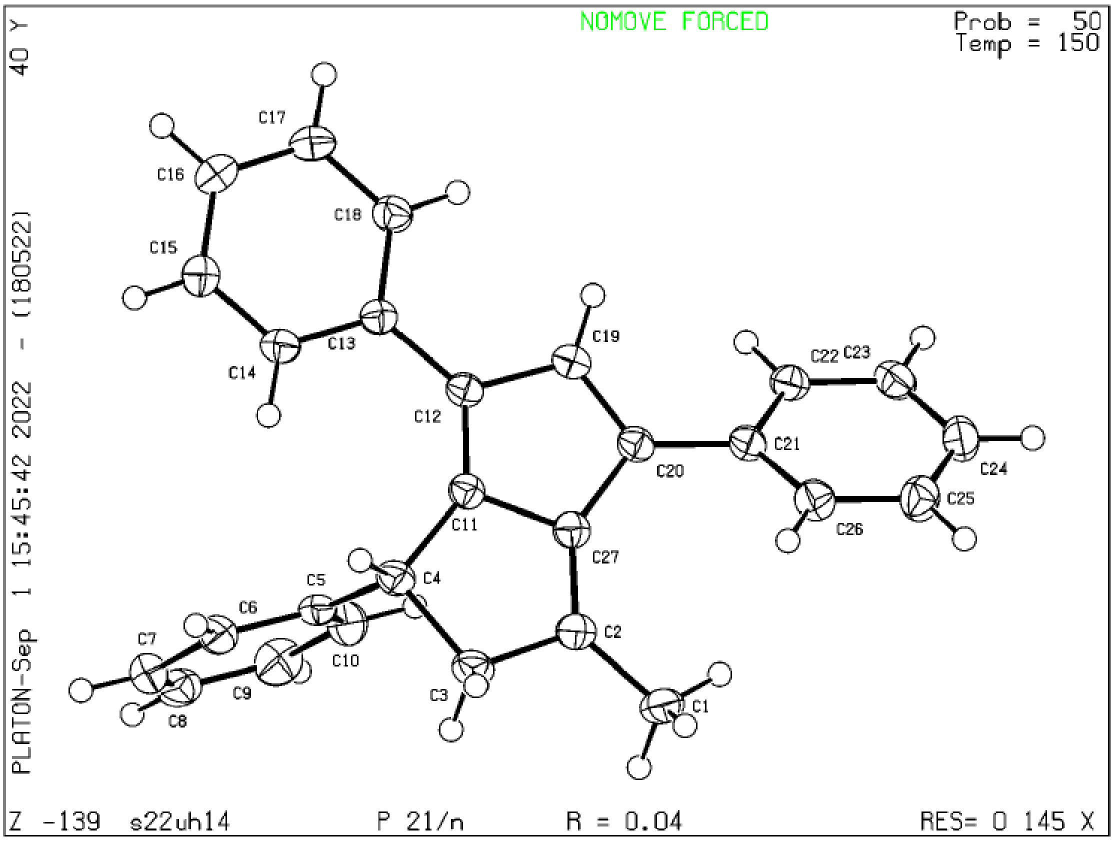

Figure S62: Ellipsoid plot for 3-methyl-1,4,6-triphenyl-1,2-dihydropentalene **12**H_2.

8.4. Crystal data and structure refinement for 1,3,8-triphenyl-4,5,6,7,7a,8-hexahydro-cyclopenta-[a]-indene (**11**H_2)

**CCDC** number	**2281044**	
Identification code	s22uh17	
Empirical formula	$C_{30}H_{26}$	
Formula weight	386.51	
Temperature	150.00(10) K	
Wavelength	1.54184 Å	
Crystal system	Monoclinic	
Space group	I2/a	
Unit cell dimensions	a = 24.0658(4) Å	α= 90°
	b = 5.97950(10) Å	β= 109.168(2)°
	c = 30.3634(4) Å	γ = 90°
Volume	4127.10(12) Å^3	
Z	8	
Density (calculated)	1.244 Mg/m^3	
Absorption coefficient	0.526 mm^{-1}	
F(000)	1648	
Crystal size	0.400 x 0.120 x 0.050 mm^3	
Theta range for data collection	3.889 to 73.028°.	
Index ranges	-29<=h<=29, -7<=k<=5, -37<=l<=37	
Reflections collected	40218	
Independent reflections	4115 [R(int) = 0.0479]	
Completeness to theta = 67.684°	100.0 %	
Absorption correction	Semi-empirical from equivalents	
Max. and min. transmission	1.00000 and 0.74502	
Refinement method	Full-matrix least-squares on F^2	
Data / restraints / parameters	4115 / 0 / 271	
Goodness-of-fit on F^2	1.088	
Final R indices [I>2sigma(I)]	R1 = 0.0414, wR2 = 0.0911	
R indices (all data)	R1 = 0.0484, wR2 = 0.0946	
Largest diff. peak and hole	0.212 and -0.202 e.Å^{-3}	

Datablock s22uh17 - ellipsoid plot

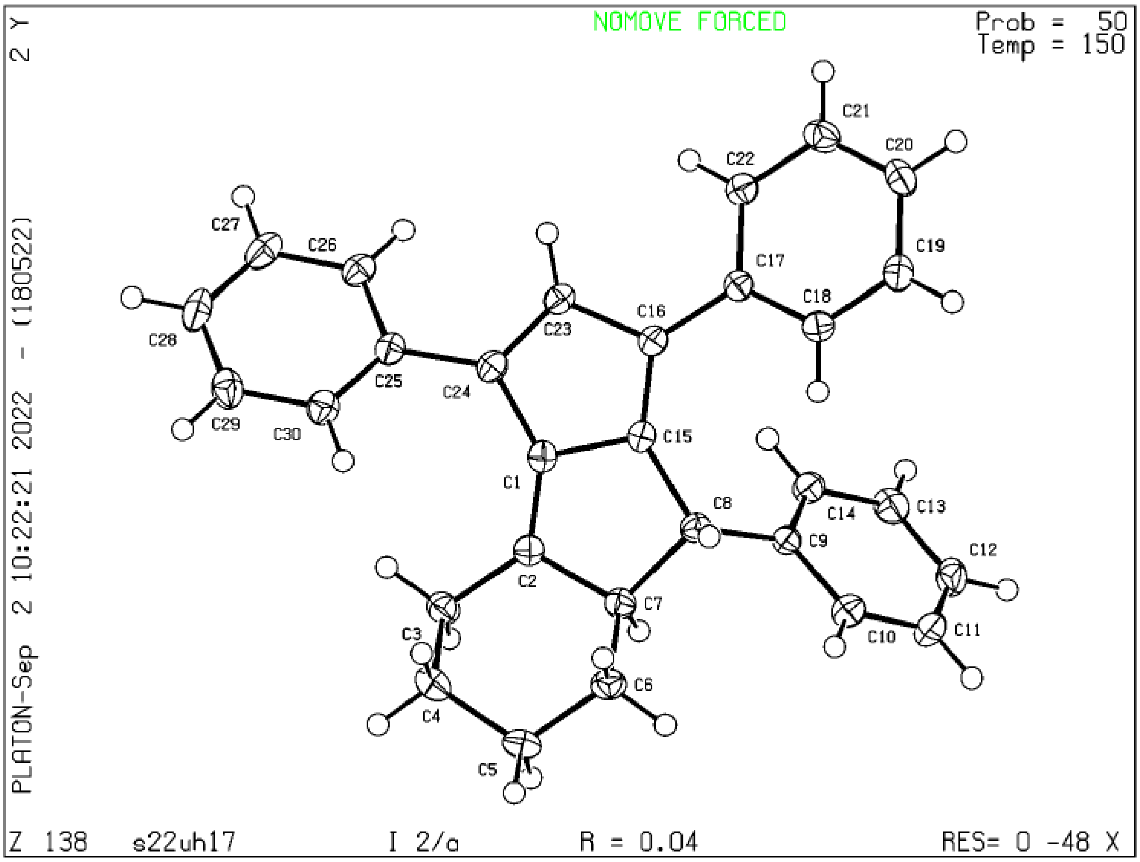

Figure S63: Ellipsoid plot for 1,3,8-triphenyl-4,5,6,7,7a,8-hexahydro-cyclopenta-[a]-indene (**11**H_2).

8.5. Crystal data and structure refinement for *anti*-bis(2,5-Norbornadiene)dirhodium 1,3-bis(4-methoxyphenyl)-4,6-diphenyl-pentalenide (Rh_2NBD_2[**7**])

**CCDC** number	**2348342**	
Identification code	s24uh10	
Empirical formula	$C_{56} H_{58} O_4 Rh_2$	
Formula weight	1000.84	
Temperature	150.00(10) K	
Wavelength	1.54184 Å	
Crystal system	Monoclinic	
Space group	$P2_1/c$	
Unit cell dimensions	a = 10.36315(7) Å	α = 90°
	b = 23.53932(17) Å	β = 100.8567(7)°
	c = 18.81353(15) Å	γ = 90°
Volume	4507.26(6) $Å^3$	
Z	4	
Density (calculated)	1.475 Mg/m^3	
Absorption coefficient	6.291 mm^{-1}	
F(000)	2064	
Crystal size	0.210 x 0.090 x 0.030 mm^3	
Theta range for data collection	3.756 to 72.853°.	
Index ranges	-10<=h<=12, -28<=k<=29, -23<=l<=23	
Reflections collected	44939	
Independent reflections	8939 [R(int) = 0.0383]	
Completeness to theta = 67.684°	100.0 %	
Absorption correction	Semi-empirical from equivalents	
Max. and min. transmission	1.00000 and 0.74321	
Refinement method	Full-matrix least-squares on F^2	
Data / restraints / parameters	8939 / 0 / 617	
Goodness-of-fit on F^2	1.105	
Final R indices [I>2sigma(I)]	R1 = 0.0306, wR2 = 0.0736	
R indices (all data)	R1 = 0.0335, wR2 = 0.0751	
Largest diff. peak and hole	0.937 and -0.464 e.$Å^{-3}$	

Datablock s24uh10 - ellipsoid plot

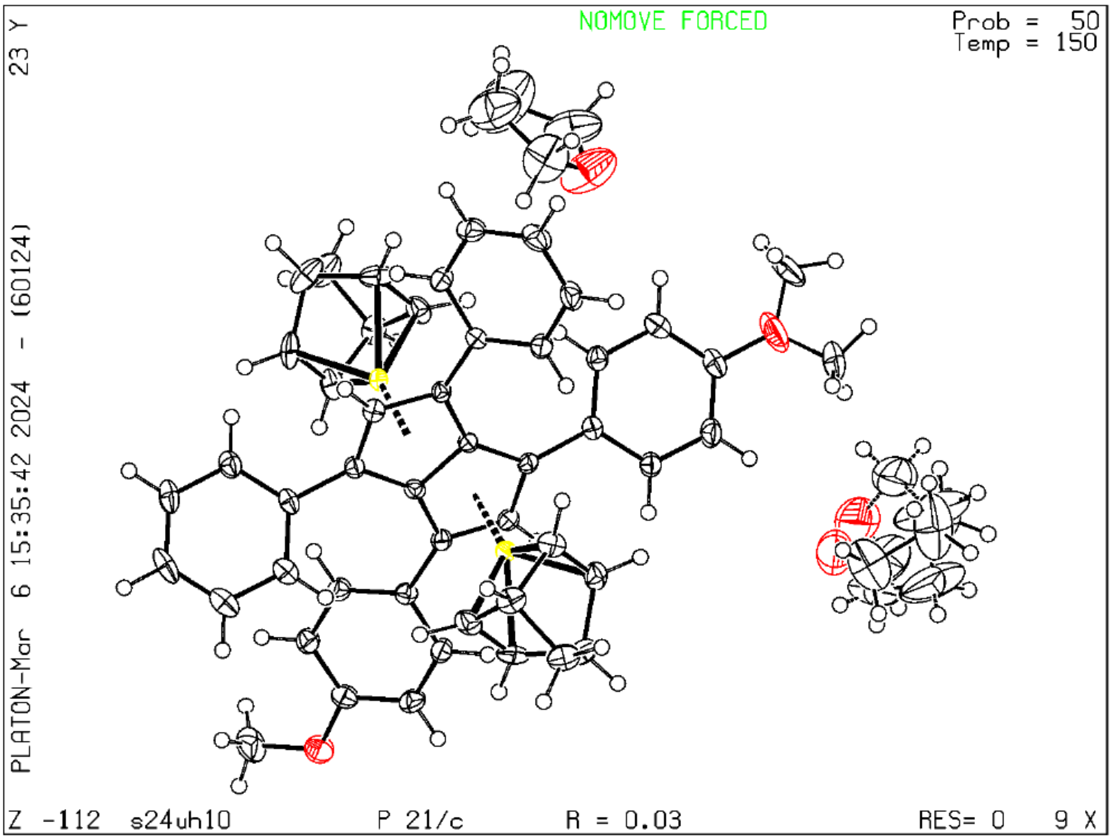

Figure S64: Ellipsoid plot for *anti*-bis(2,5-Norbornadiene)dirhodium 1,3-bis(4-methoxyphenyl)-4,6-diphenyl-pentalenide Rh_2NBD_2[**7**].

9. Calculations (Figures S65–S79)

Computational methods. Optimization, NICS Scan,[1] NICS XY-Scan[2] and ACID[3] calculations were carried out using the Gaussian 09 program package, revision D.01.[4] Becke's three parameter exchange-correlation hybrid functional B3LYP[5] was used in combination with the 6-311+G* basis set. The stationary points were characterized as minima by an analytical vibrational frequency calculation,[6] which revealed the absence of imaginary frequencies. NBO population analysis[7] was calculated with ωB97X-D from the group of Head-Gordon[8] and the aug-cc-pVTZ basis set of Kendall et al.[9] Calculations of HOMO-LUMO gaps were carried out with lithium counter ions in *anti* configuration above the pentalenide core in simulated THF using the Polarizable Continuum Model (PCM).[10] NICS Z scans were performed perpendicular to the respective ring, from its centre up to 4 Å above the plane, and the NICS X/Y scans at 1.7 Å above the respective subunits. All ACID calculations were plotted with an isovalue of 0.025. ESP-maps were plotted with Gausview and a colour range going from −0.36 (red) to 0.2 (blue) and a surface isovalue of 0.01.

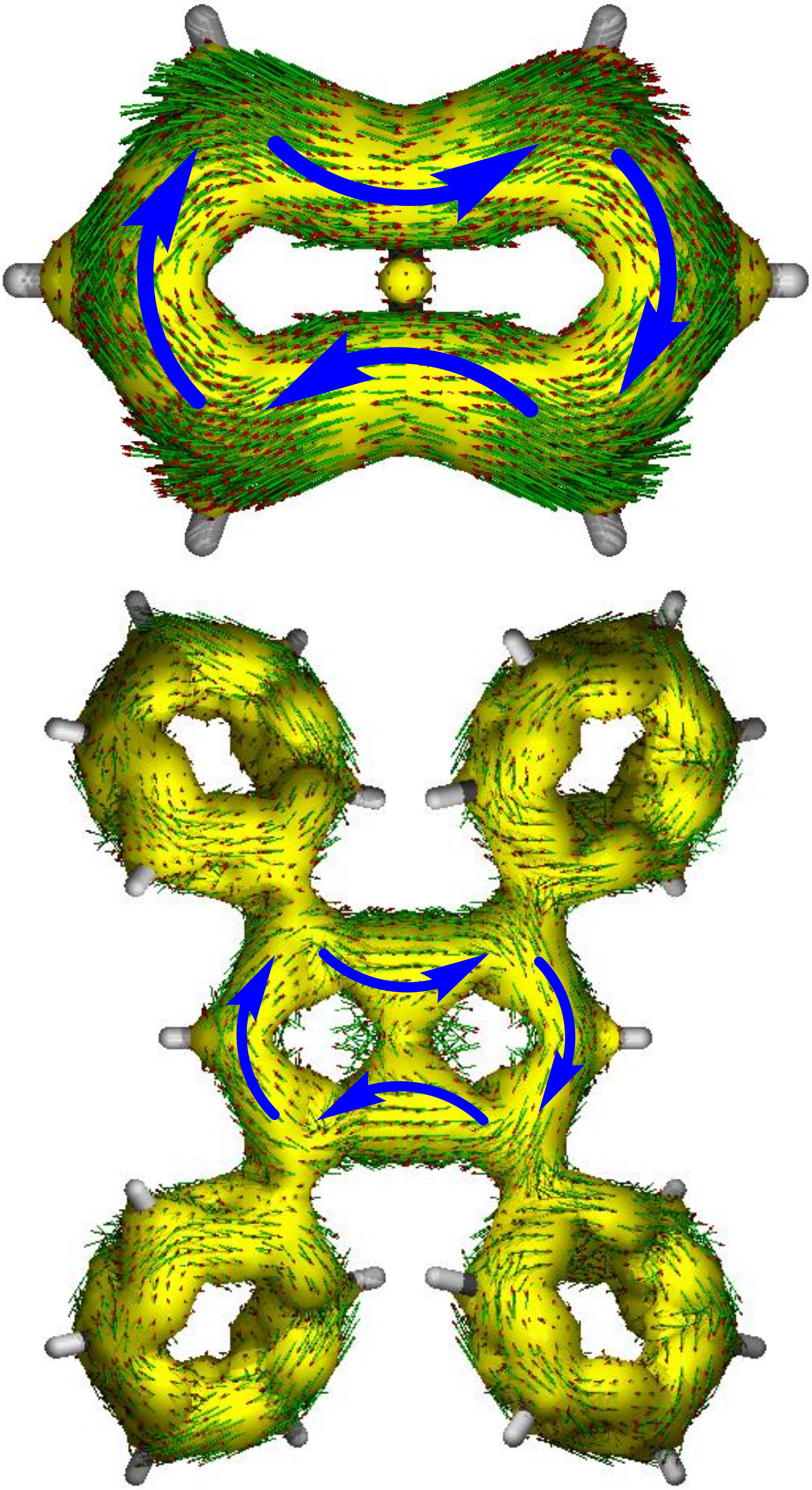

Figure S65: Comparison of ACID plots for **1** (top) and **2** (bottom).

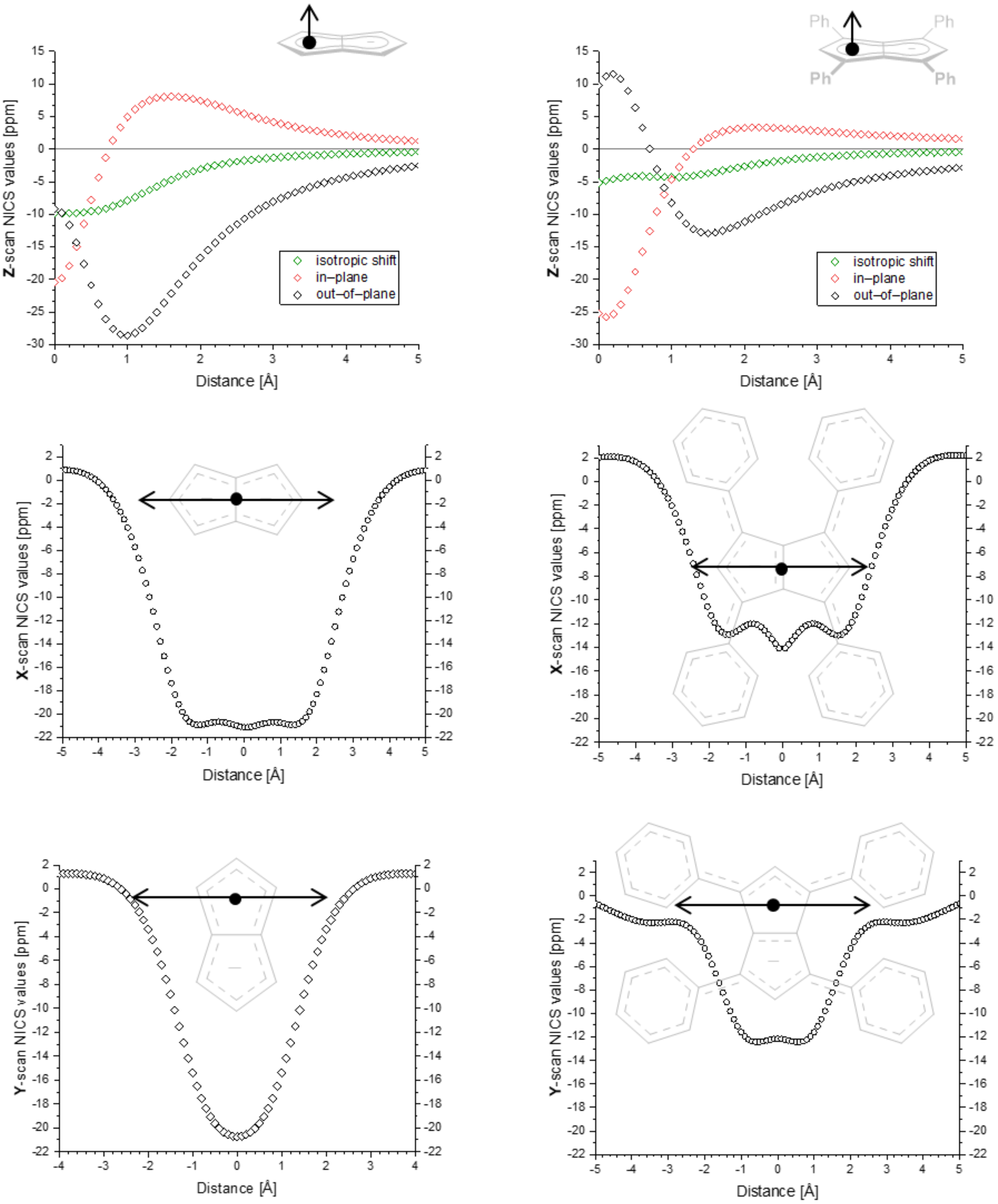

Figure S66: Comparison of NICS scan values and for **1** and **2**.

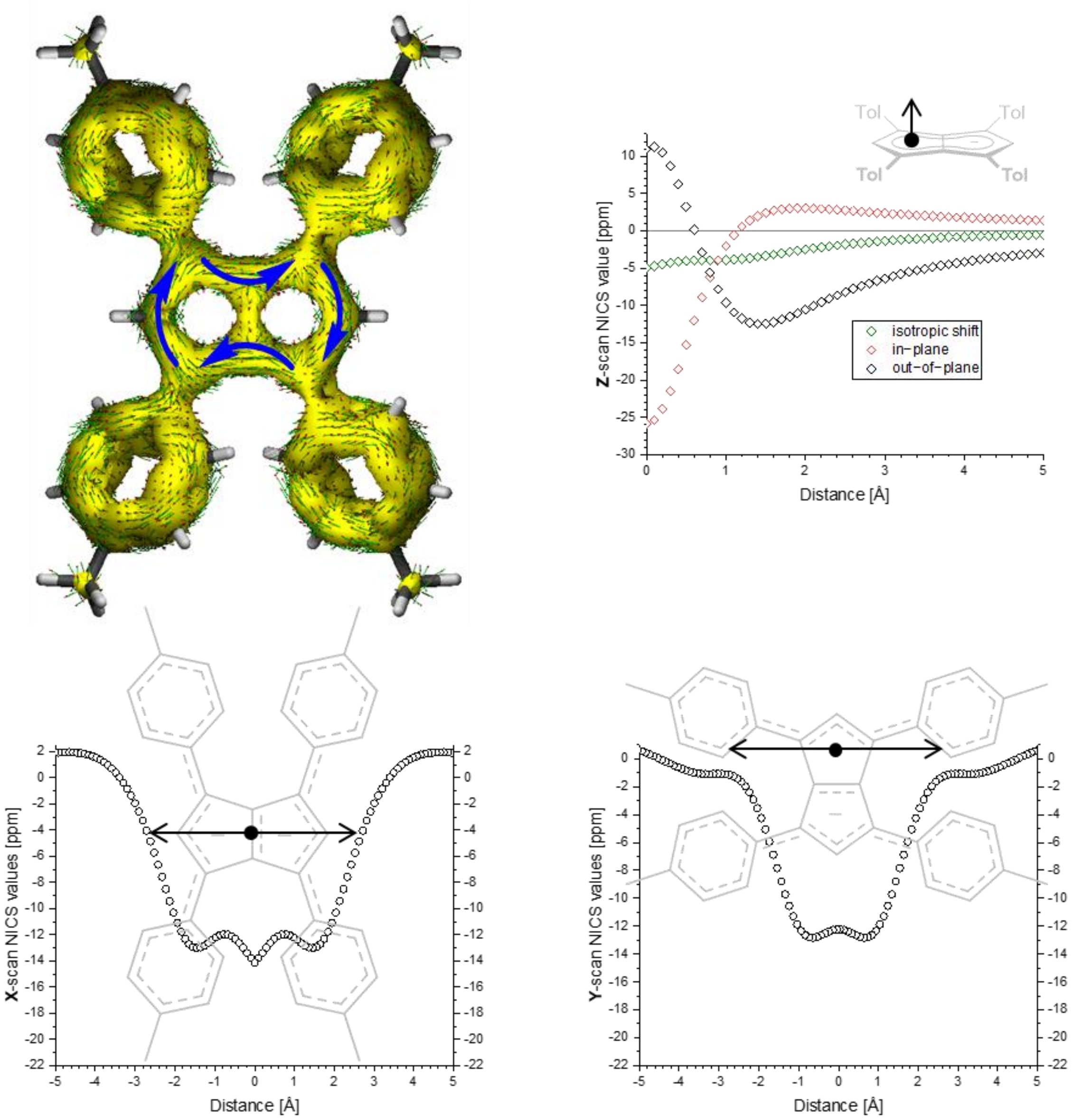

Figure S67: NICS scan values and ACID plot for **3**.

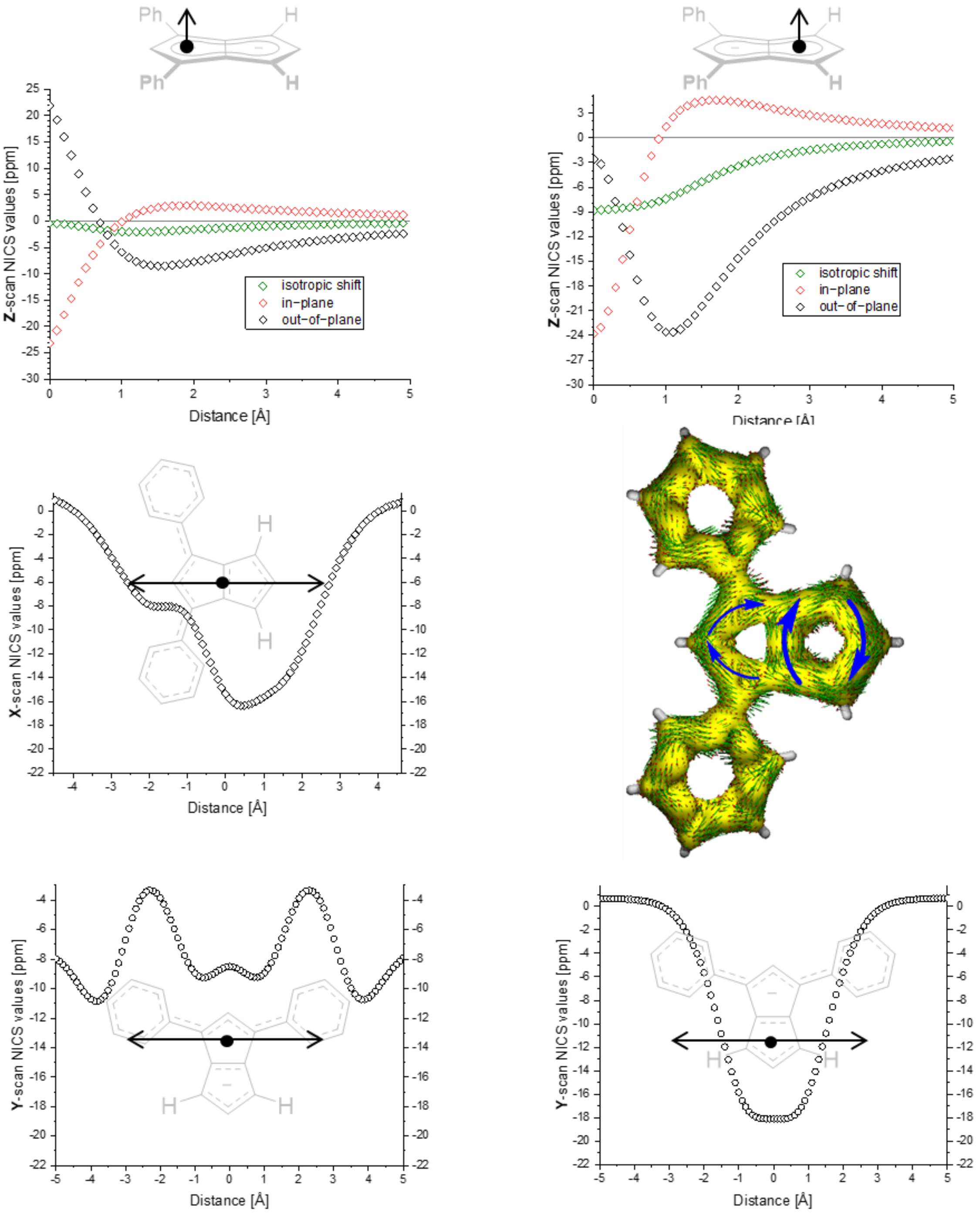

Figure S68: NICS scan values and ACID plot for **9**.

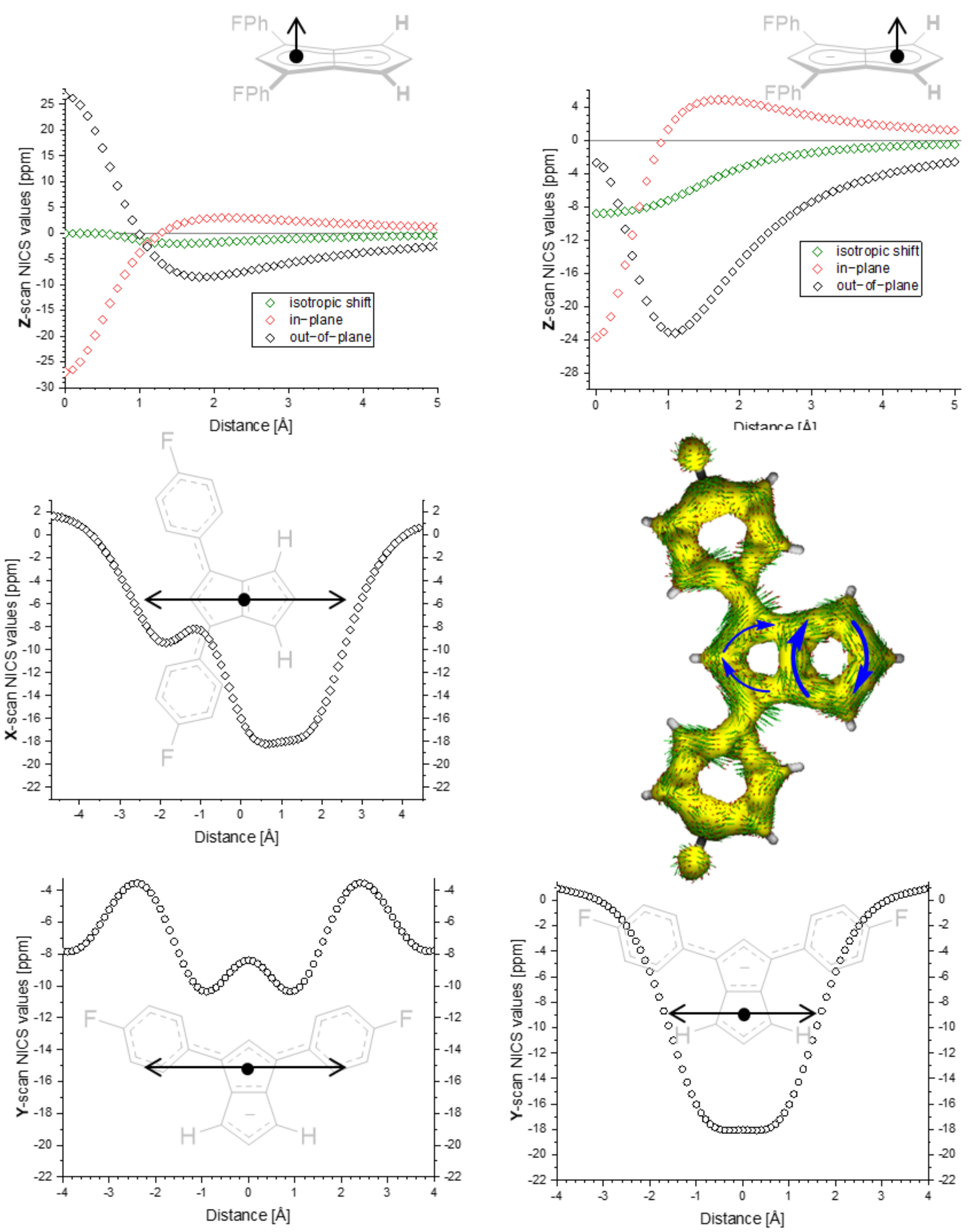

Figure S69: NICS scan values and ACID plot for **10**.

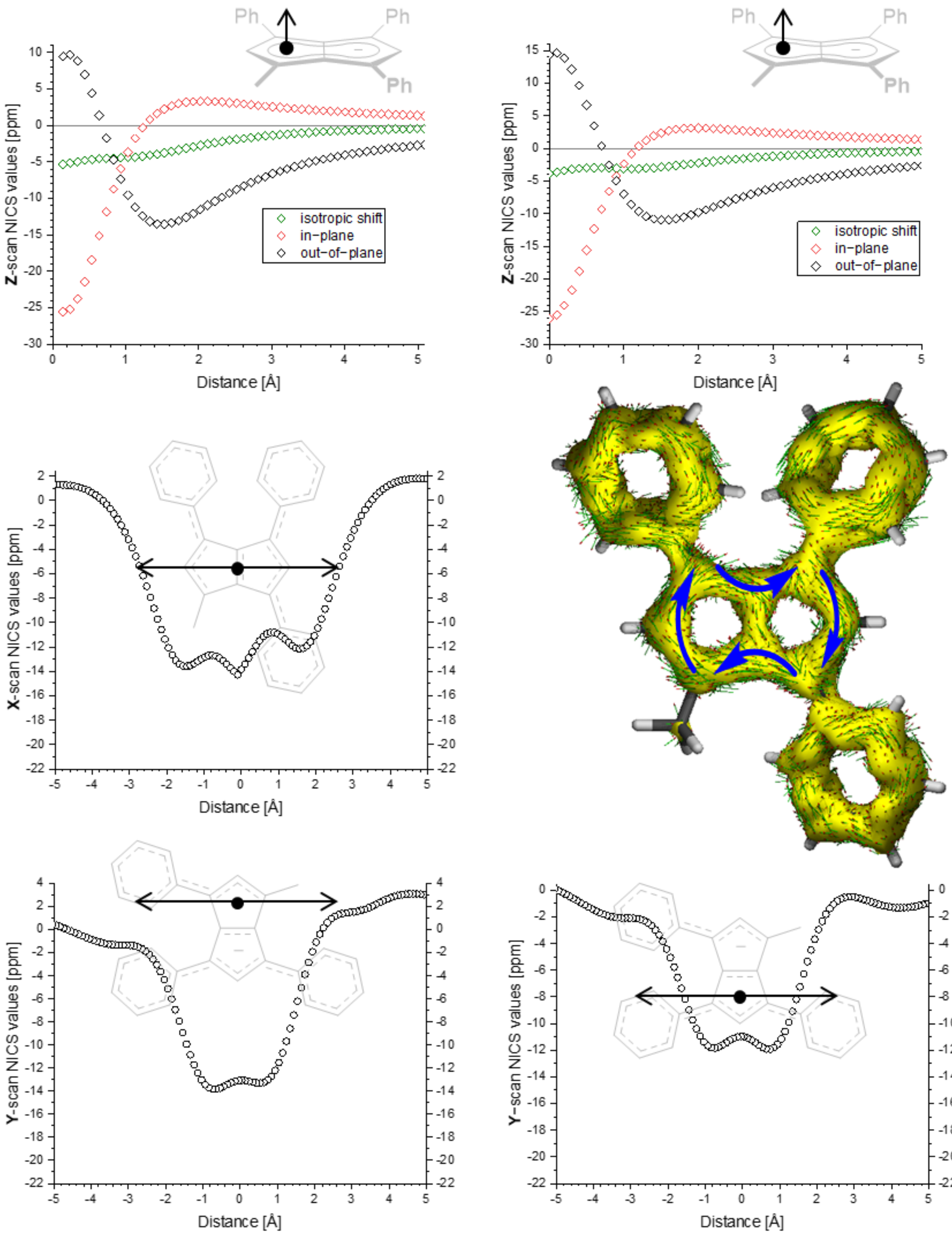

Figure S70: NICS scan values and ACID plot for **12**.

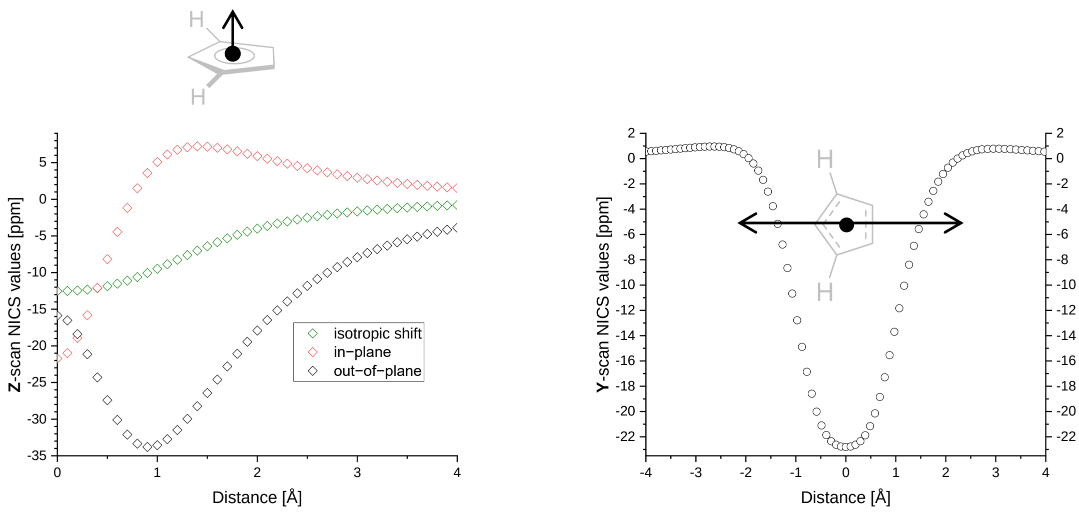

Figure S71: NICS scan values for cyclopentadienyl anion.

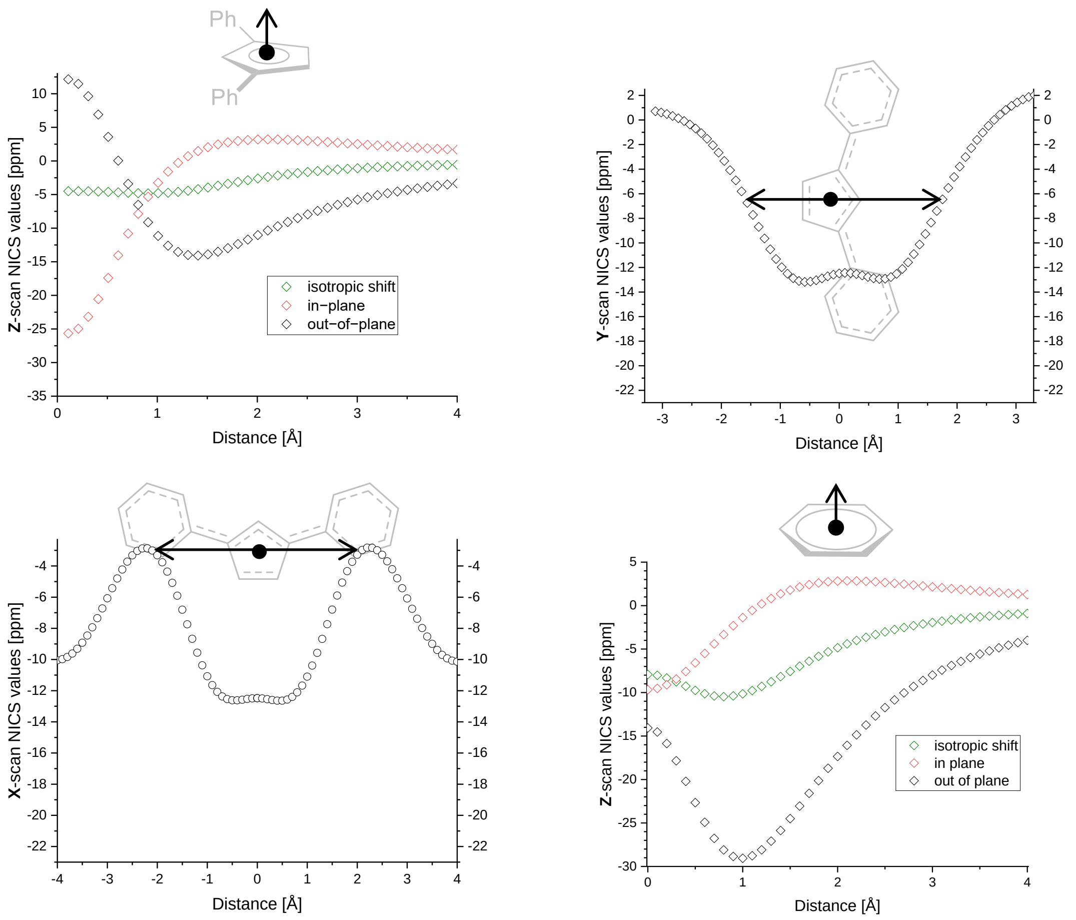

Figure S72: NICS scan values for 1,3-diphenyl-cyclopentadienyl anion and NICS Z scan of benzene.

Table S2: Overall comparison for the HOMO/LUMO-energies and the resulting gap in [eV], as well as the calculated sum of NBO charges per pentalenide-subunit compared to the experimentally obtained ^{1}H NMR shifts.

**Pn^{2-}**	**HOMO [eV]**	**LUMO [eV]**	**Gap [eV]**	**Sum of NBO-Charges Cp^1 [e]**	**Sum of NBO-Charges Cp^2 [e]**	**H^2 [ppm]**	**H^5 [ppm]**
**1**	−3.74	−0.32	3.42	2*(−1.29)		5.76 [11]	5.76 [11]
**2**	−3.94	−0.79	3.16	2*(−0.45		6.79 [12]	6.79 [12]
**3**	−3.82	−0.66	3.16	2*(−0.47)		6.66	6.66
**9**	−3.88	−0.82	3.06	−0.41	−1.14	7.05	6.06
**10**	−3.92	−0.84	3.08	−0.50	−1.16	6.92	6.06
**12**	−3.79	−0.69	3.11	−0.48	−0.48	6.19	6.67

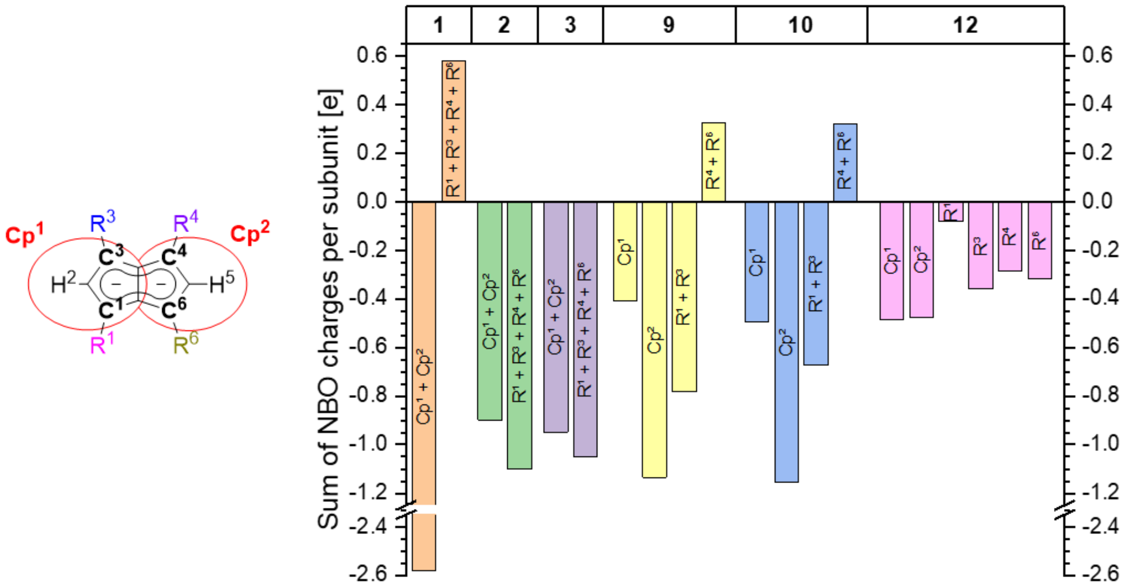

Figure S73: Left: Separation of the pentalenide core into two Cp subunits (indicated by red circles) and calculated sums of NBO charges for each subunit compared to the experimentally obtained ^{1}H NMR shifts. Right: Plot of sums of NBO charges for each part (charges on the shared $C^{3'}$ and $C^{6'}$ atoms were equally distributed between both subunits).

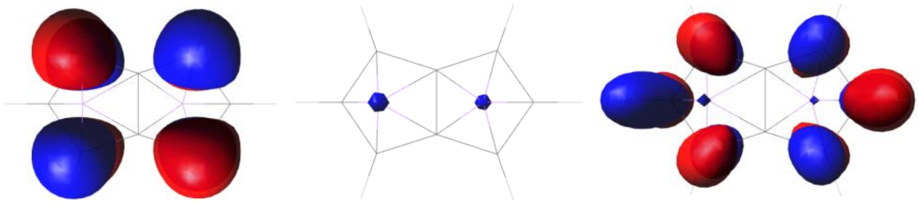

HOMO: −3.74 eV LUMO: −0.32 eV LUMO+12: 1.71 eV

Figure S74: Frontier orbitals of **Li$_2$[1]** (Iso-Value 0.035).

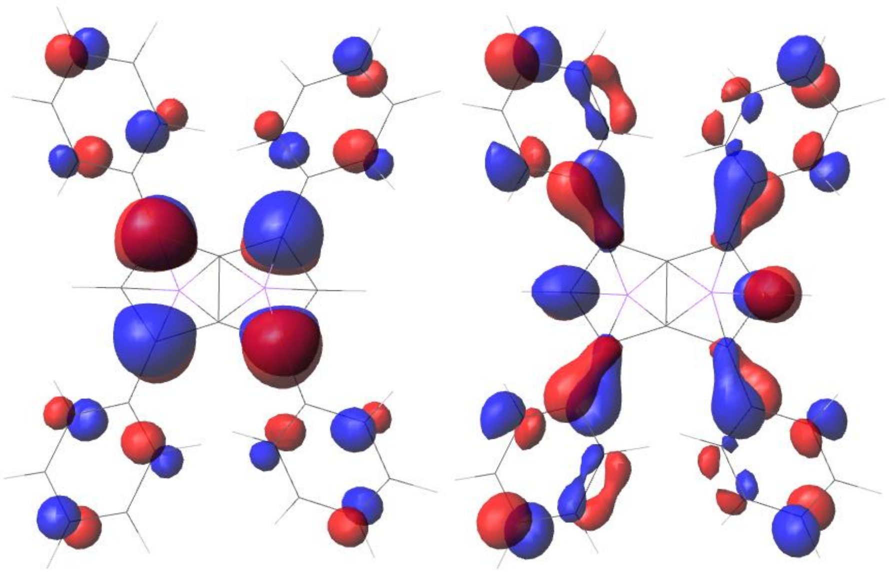

HOMO: −3.95 eV LUMO: −0.79 eV

Figure S75: Frontier orbitals of **Li$_2$[2]** (Iso-Value 0.035).

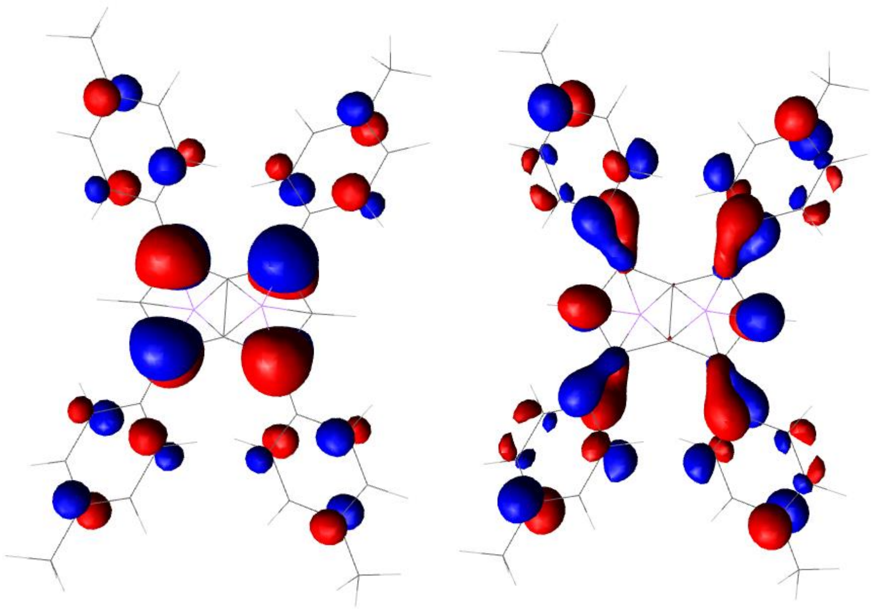

HOMO: −3.82 eV LUMO: −0.66 eV

Figure S76: Frontier orbitals of **Li₂[3]** (Iso-Value 0.035).

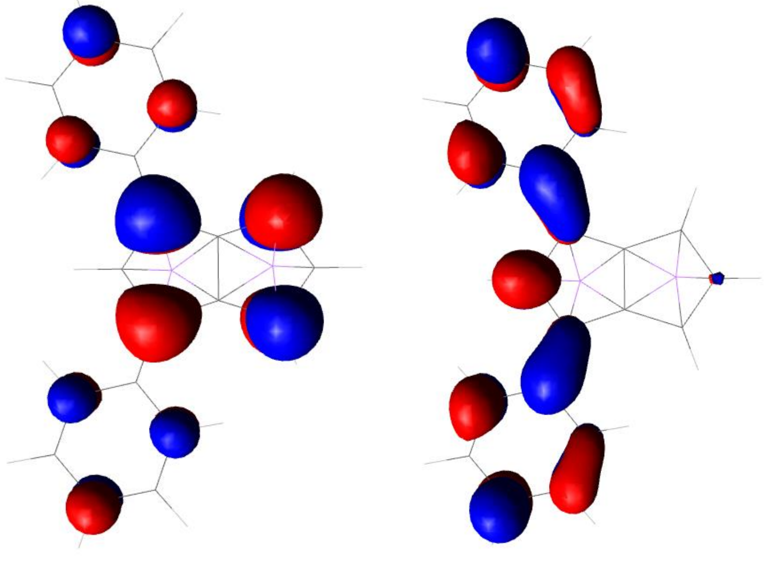

HOMO: −3.88 eV LUMO: −0.82 eV

Figure S77: Frontier orbitals of **Li₂[9]** (Iso-Value 0.035).

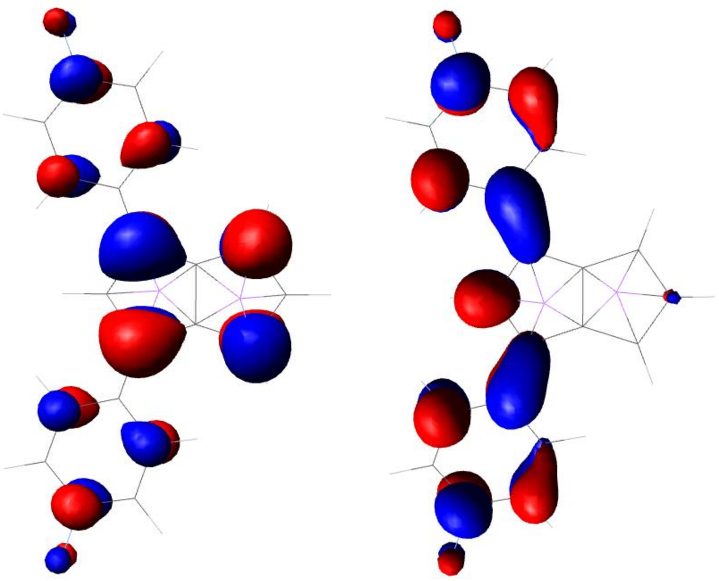

HOMO: −3.92 eV LUMO: −0.84 eV

Figure S78: Frontier orbitals of **Li_2[10]** (Iso-Value 0.035).

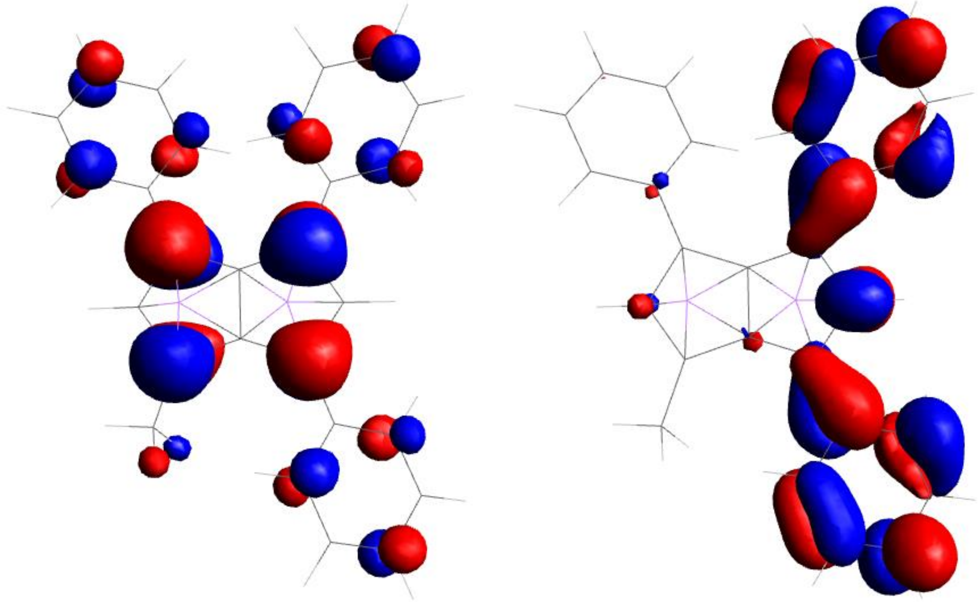

HOMO: −3.92 eV LUMO: −0.84 eV

Figure S79: Frontier orbitals of **Li_2[12]** (Iso-Value 0.035).

Table S3: Overall comparison of characteristic values extracted from the NICS scans. The NICS-X value was taken from 1.7 Å above the centre of the respective Cp^--subunit. NICS(1) constitutes the isotropic shift at a Z height of 1.0 Å. For **1–3** the differences between both subunits in the Z- and X-scan were in the margin of error, so only one value is listed here.

**Compd.**	**NICS-X values above the ring centre(s) [ppm]**	**NICS(1) [ppm]**	**Values of the out–of–plane component at the minima of NICS-Z-scan [ppm]**
**1**	−20.81	−7.89	−28.61
**2**	−12.12	−4.31	−12.92
**3**	−12.18	−3.88	−12.47
**9**	−8.85 (1,3-Ph)<br>−15.66 (4,6-H)	−2.01 (1,3-Ph)<br>−7.41 (4,6-H)	−8.53 (1,3-Ph)<br>−23.63 (4,6-H)
**10**	−8.31 (1,3-^{p}FPh)<br>−18.04 (4,6-H)	−1.37 (1,3-^{p}FPh)<br>−7.26 (4,6-H)	−8.49 (1,3-^{p}FPh)<br>−23.17 (4,6-H)
**12**	−12.80 (1-Me,3-Ph)<br>−10.91 (4,6-Ph)	−4.41 (1-Me,3-Ph)<br>−3.12 (4,6-Ph)	−13.55 (1-Me,3-Ph)<br>−11.00 (4,6-Ph)
**Cp^-**	−22.81	−9.49	−33.80
**1,3-Ph-Cp^-**	−12.78	−4.80	−14.08

Computational references:

[1] A. Stanger, *J. Org. Chem.* **2006**, *71*, 883–893.

[2] R. Gershoni-Poranne, A. Stanger, *Chem. Eur. J.* **2014**, *20*, 5673–5688.

[3] a) D. Geuenich, R. Herges, *J. Phys. Chem. A* **2001**, *105*, 3214-3220; b) D. Geuenich, K. Hess, F. Köhler, R. Herges, *Chem. Rev.* **2005**, *105*, 3758–3772.

[4] Gaussian 98 g16, Revision C.01, M. J. Frisch, G. W. Trucks, H. B. Schlegel, G. E. Scuseria, M. A. Robb, J. R. Cheeseman, G. Scalmani, V. Barone, G. A. Petersson, H. Nakatsuji, X. Li, M. Caricato, A. V. Marenich, J. Bloino, B. G. Janesko, R. Gomperts, B. Mennucci, H. P. Hratchian, J. V. Ortiz, A. F. Izmaylov, J. L. Sonnenberg, D. Williams-Young, F. Ding, F. Lipparini, F. Egidi, J. Goings, B. Peng, A. Petrone, T. Henderson, D. Ranasinghe, V. G. Zakrzewski, J. Gao, N. Rega, G. Zheng, W. Liang, M. Hada, M. Ehara, K. Toyota, R. Fukuda, J. Hasegawa, M. Ishida, T. Nakajima, Y. Honda, O. Kitao, H. Nakai, T. Vreven, K. Throssell, J. A. Montgomery, Jr., J. E. Peralta, F. Ogliaro, M. J. Bearpark, J. J. Heyd, E. N. Brothers, K. N. Kudin, V. N. Staroverov, T. A. Keith, R. Kobayashi, J. Normand, K. Raghavachari, A. P. Rendell, J. C. Burant, S. S. Iyengar, J. Tomasi, M. Cossi, J. M. Millam, M. Klene, C. Adamo, R. Cammi, J. W. Ochterski, R. L. Martin, K. Morokuma, O. Farkas, J. B. Foresman, and D. J. Fox, *Gaussian, Inc., Wallingford CT*, **2019**.

[5] a) P. A. M. Dirac, *Proc. R. Soc. London, Ser. A* **1929**, *123*, 714–733; b) J. C. Slater, *Phys. Rev.* **1951**, *81*, 385–390; c) A. D. Becke, *Phys. Rev. A.* **1988**, *38*, 3098–3100; d) C. Lee, W. Yang, R. G. Parr, *Phys. Rev. B* **1988**, *37*, 785–89; e) A. D. Becke, *J. Chem. Phys.* **1993**, *98*, 5648–5652.

[6] P. Deglmann, F. Furche, R. Ahlrichs, *Chem. Phys. Lett.* **2002**, *362*, 511–518; b) P. Deglmann, F. Furche, *J. Chem. Phys.* **2002**, *117*, 9535–9538.

[7] NBO 7.0. E. D. Glendening, J. K. Badenhoop, A. E. Reed, J. E. Carpenter, J. A. Bohmann, C. M. Morales, P. Karafiloglou, C. R. Landis, and F. Weinhold, *Theoretical Chemistry Institute*, University of Wisconsin, *Madison*, *WI*, **2018**.

[8] J.-D. Chai and M. Head-Gordon, *Phys. Chem. Chem. Phys*. **2008**, *10*, 6615–6620.

[9] R. A. Kendall, T. H. Dunning Jr., and R. J. Harrison, *J. Chem. Phys*. **1992**, *96*, 6796–806.

[10] J. Tomasi, B. Mennucci, and R. Cammi, *Chem. Rev*. **2005**, *105*, 2999–3093.

[11] F. G. N. Cloke, M. C. Kuchta, R. M. Harker, P. B. Hitchcock, and J. S. Parry, *Organometallics* **2000**, 19, 5795–5798.

[12] S. M. Boyt, N. A. Jenek, H. J. Sanderson, G. Kociok-Köhn, and U. Hintermair, *Organometallics* **2022**, 41, 211–225.
